# Smart Nanoformulations for Oncology: A Review on Overcoming Biological Barriers with Active Targeting, Stimuli-Responsive, and Controlled Release for Effective Drug Delivery

**DOI:** 10.3390/pharmaceutics18020196

**Published:** 2026-02-02

**Authors:** Srikanth Basety, Renuka Gudepu, Aditya Velidandi

**Affiliations:** 1 Tris Pharma, Monmouth Junction, NJ 08852, USA; srikanth_basety@yahoo.com; 2 Department of Microbiology, Pingle Government College for Women (A), Warangal 506001, India; renumanduva@gmail.com; 3 Department of Biotechnology, Vaagdevi Degree and P.G. College, Warangal 506001, India

**Keywords:** active targeting, controlled release, drug delivery, nanoformulations, oncology, stimuli-responsive

## Abstract

Effective drug delivery in oncology is challenged by a hierarchy of biological barriers—from abnormal vasculature and dense stroma to cellular immunosuppression and specialized interfaces like the blood–brain barrier. This review provides a contemporary analysis of smart nanoformulations through the lens of a rational, stage-gated design pipeline. We first deconstruct the solid tumor microenvironment as a multi-tiered obstacle (systemic, stromal, cellular), establishing a barrier-specific foundation for nanocarrier design. The core of the review articulates an architectural toolkit, detailing how intrinsic nanoparticle properties precondition in vivo identity via the protein corona, which in turn informs the selection of advanced ligands for cellular targeting and programmed intracellular trafficking. This integrated framework sets the stage for exploring sophisticated applications, including endogenous and externally triggered responsive systems, bio-orthogonal activation, immuno-nanoformulations, and combination strategies aimed at overcoming multidrug resistance. By synthesizing these components into a cohesive design philosophy, this review moves beyond a catalog of advances to offer a blueprint for engineering next-generation nanotherapeutics. We critically assess the translational landscape and contend that this hierarchical design approach is essential for developing more effective, personalized, and clinically viable cancer treatments.

## 1. Introduction

Nanotechnology, particularly in the form of nanomedicine (nanoformulations and nanoparticles), represents a transformative approach in the medical field, offering innovative solutions for diagnosis, treatment, and drug delivery [1]. Nanoparticles, due to their nanoscale size, unique properties, and ability to interact with biological systems, are central to these advancements [2]. They enable targeted drug delivery, improve imaging techniques, and offer new therapeutic possibilities, especially in challenging areas like cancer treatment. Nanoformulations can penetrate biological barriers and distribute across various organs, with their size playing a pivotal role in determining their permeability and distribution (Table 1) [3]. This size-dependent behavior influences both the therapeutic efficacy and potential toxicity of nanoformulations [3].

**Table 1:** Role of nanosizes in permeability of various organs [4].

Organ	Recommended Size *	Requirement(s)
Breast	<12 nm (<200 nm can also penetrate but at lower efficiency)	Particles should be able to across the BBB and exhibit a molecular weight of <400 Da
Pancreas	<150 nm	For rapid uptake 10 to 20 nm. However, particles ~150 nm can cross the fenestrae present in the liver endothelium
Colon	300 to 1000 nm	For long accumulation times, cationic nanoparticles are recommended
Lung	~100 nm	Polymeric nanoparticles and carbon nanotubes
Liver	20 to 100 nm	L-fucose-receptor-mediated delivery of nanoparticles was recommended to target fucosyltransferases
Brain	<150 nm	Albumin nanoparticles are recommended to diminish dose-limiting toxicities of extremely hydrophobic drug formulations

* In general, ~200 nm is the recommended size to avoid the reticuloendothelial system, however, the recommended size can vary by the tumor size that also influences the irregular vasculature gaps.

The development of smart nanoformulations has revolutionized drug delivery by enabling precise targeting, controlled release, and responsiveness to specific biological stimuli, thereby overcoming numerous biological barriers that traditionally hinder effective therapy [5,6]. The recent literature underscored the multifaceted strategies employed in designing these advanced nanocarriers, emphasizing active targeting, stimuli-responsiveness, and controlled release mechanisms to enhance therapeutic efficacy [7,8]. A significant focus within this domain is the ability of nanocarriers to navigate complex biological environments, such as tumor microenvironments (TMEs) and physiological barriers like the blood–brain barrier (BBB). Zhang et al. [9] highlighted the role of carbohydrate-based stimuli-responsive nanocarriers in cancer-targeted chemotherapy, which respond to intracellular stimuli such as pH, redox potential, and enzymatic activity. These nanocarriers facilitate on-demand drug activation and release, thereby improving specificity and reducing systemic toxicity. Similarly, Kumar et al. [10] emphasized that nanodrug delivery systems are engineered to breach biological barriers and deliver drugs precisely at target sites, which is crucial for effective therapy.

Stimuli-responsive nanomaterials are central to overcoming biological barriers. Singh et al. [11] categorized smart nanomaterials based on their responsiveness to various TME cues, including pH, enzymes, and redox conditions, which enable site-specific drug release. This responsiveness not only enhances therapeutic precision but also minimizes off-target effects [12]. Dong et al. [13] further elaborated on the development of self-assembled prodrugs that respond to multiple stimuli, offering a versatile platform for mono- or combination therapies. These systems are designed to adapt dynamically to the tumor milieu, ensuring that drugs are released only under specific conditions, thus maximizing efficacy.

The integration of external stimuli such as light, ultrasound, and magnetic fields has also gained prominence. Agiba et al. [14] reviewed light-responsive liposomes and dual-targeting systems that utilize external triggers for controlled drug release. Similarly, Kashkooli et al. [15] focused on ultrasound-mediated nano drug delivery, discussing how physical stimuli can enhance permeability and facilitate targeted therapy. Yazdan and Naghib [16] explored ultrasound-responsive polymers that can traverse challenging barriers like the BBB, demonstrating the potential of combining nanotechnology with external stimuli to achieve precise delivery. Hydrogels and nanogels have emerged as promising platforms for stimuli-responsive drug delivery. Attama et al. [17] reviewed the evolution of nanogels over the past decade, emphasizing their capacity for targeted delivery and controlled release in cancer therapy. Kakkar and Narula [18] discussed molecularly imprinted hydrogels that can provide feedback-regulated drug release, further enhancing specificity. Damiri et al. [19] focused on polysaccharide nanohydrogels that respond to various stimuli, offering a biocompatible and versatile approach for drug delivery.

Active targeting strategies are often combined with stimuli-responsiveness to improve selectivity. Zhang et al. [9] described nanocarriers that respond to intracellular cues, while Sahoo et al. [20] detail nanocarriers designed for colorectal cancer that exploit tumor-specific markers for active targeting, alongside stimuli-responsive release triggered by pH, redox, or enzymatic activity. These dual strategies ensure that drugs are delivered efficiently to tumor sites while minimizing effects on healthy tissues. The biological barriers such as the BBB pose significant challenges, which are being addressed through innovative nanotechnologies. Premchandani et al. [21] highlighted engineered exosomes as smart carriers capable of crossing such barriers, leveraging their natural biocompatibility and targeting capabilities. Furthermore, the safety, regulatory considerations, and translational potential of these nanocarriers are critical aspects discussed in recent reviews. Attama et al. [17] and Karnwal et al. [22] emphasized the importance of designing nanocarriers that are not only effective but also safe and scalable for clinical applications. The integration of stimuli-responsiveness with biocompatibility and targeted delivery is seen as a pathway toward personalized medicine, as highlighted by Premchandani et al. [21], who advocate for the use of engineered exosomes tailored to individual patient needs.

In summary, the current literature demonstrates that smart nanoformulations, through active targeting, stimuli-responsiveness, and controlled release mechanisms, are destined to overcome longstanding biological barriers in drug delivery. These systems leverage the unique features of the TME and external stimuli to achieve site-specific, efficient, and safe therapeutic outcomes. The ongoing advancements in nanotechnology, combined with a deeper understanding of biological barriers, are paving the way for next-generation nanomedicine capable of transforming cancer therapy and other biomedical applications.

## 2. Solid Tumor Microenvironment: A Multi-Tiered Barrier to Drug Delivery

The solid TME presents a formidable multi-tiered barrier to effective drug delivery (Table 2), significantly impeding therapeutic efficacy in cancer treatment. The recent literature underscores the complexity of these barriers, which include physical, biological, and immunological components, each contributing to the challenge of achieving sufficient drug concentrations within tumor tissues. One of the primary physical barriers is the abnormal vasculature characteristic of solid tumors. Despite the hyperpermeability of tumor blood vessels, the high density of stromal cells and extracellular matrix (ECM) proteins, coupled with elevated interstitial fluid pressure, restricts deep penetration of therapeutic agents. Haze et al. [23] highlighted that the dense stromal components and increased interstitial fluid pressure limit compound delivery to the tumor core, necessitating strategies to modulate the TME to enhance drug access. Similarly, Keller and Averkiou [24] discussed the use of focused ultrasound, with or without microbubbles, to modulate tumor interstitial fluid pressure, thereby improving drug delivery by mechanically affecting the ECM and vasculature. These approaches aim to normalize tumor vasculature and reduce physical barriers, facilitating better penetration of therapeutic agents.

**Table 2:** The multi-tiered barriers of the solid TME and corresponding nano-strategies.

Barrier	Specific Challenge	Impact on Drug Delivery	Nanoformulation Strategies	Ref.
Systemic and vascular	Abnormal, leaky vasculature	Heterogeneous and inefficient nanoparticle extravasation via the enhanced permeability and retention (EPR) effect	Vascular normalization agents (e.g., anti-angiogenics) to improve vessel function Size/charge-tuning: Optimal sizing (~20 to 150 nm) for EPR exploitation Ligand-mediated active targeting to enhance endothelial binding and uptake	[23,24]
	Elevated interstitial fluid pressure	Reduces convective transport, impeding deep penetration into the tumor core	Stroma-modifying agents (e.g., losartan) co-delivered with nanocarriers to reduce pressure External physical modulation (e.g., focused ultrasound) to transiently lower interstitial fluid pressure and enhance permeability	[24,25]
Stromal and ECM	Dense ECM (collagen, hyaluronan)	Physically blocks nanoparticle diffusion and promotes binding/retention	ECM-degrading enzymes (e.g., hyaluronidase, collagenase) delivered via or conjugated to nanocarriers Penetrating peptides (e.g., iRGD) to trigger trans-tissue transport pathways Charge-switchable nanoparticles that become positively charged in the TME for better diffusion	[26,27]
	Cancer-associated fibroblasts (CAFs)	Secrete ECM components and immunosuppressive signals; create a physical and biological barrier	CAF-targeting nanocarriers to deliver therapeutics that deplete or reprogram CAFs Biomimetic liposomes targeting fibroblast activation protein	[27,28]
Cellular and immunological	Immunosuppressive cells	Creates an immunologically ‘cold’ tumor, resistant to chemo- and immunotherapy	Immune-reprogramming nanoparticles to re-educate tumor associated macrophages from M2 to M1 phenotype Nano-immunotherapies delivering checkpoint inhibitors (anti-PD-1, etc.) directly to the TME Cell-based delivery systems (e.g., CAR-neutrophils) to navigate the immune landscape	[25,29]
Specialized barriers	Blood–brain barrier/blood–brain tumor barrier	Severely restricts access of most therapeutics to brain tumors like glioma	Surface-engineered liposomes and lipid nanoparticles with ligands for receptor-mediated transcytosis (e.g., transferrin) Transient barrier disruption using ultrasound-responsive nanoparticles Biomimetic nanoparticles coated with cell membranes to evade immune clearance and enhance crossing	[30,31]

The ECM itself acts as a physical barrier, often composed of dense collagen and hyaluronan, which can physically obstruct drug diffusion. Sun et al. [26] demonstrated that enzymatic degradation of ECM components, such as hyaluronidase, combined with nanotechnology, can effectively unwrapping the tumor’s physical barriers, thus optimizing photothermal and photodynamic therapies and enhancing immune responses. This indicates that targeted ECM modulation is a promising strategy to overcome the stromal barrier. Biological barriers within the TME further complicate drug delivery. The presence of CAFs and immunosuppressive cells creates a hostile environment that not only impedes drug penetration but also promotes tumor progression and resistance. Zhang et al. [27] described the use of biomimetic liposomes targeting CAFs in pancreatic ductal adenocarcinoma, which enhances drug sensitivity and perfusion by modulating the stromal components. Similarly, the immunosuppressive microenvironment in gliomas and other tumors can hinder immune-based therapies, necessitating innovative delivery systems that can remodel or bypass these barriers.

The BBB and blood–brain tumor barrier (BBTB) exemplify specialized biological barriers that restrict drug entry into brain tumors. Shaw and Paul [30] reviewed recent advances in liposome-based nanocarriers designed to traverse these barriers for glioma treatment. Liposomes, owing to their size and surface modification capabilities, have shown promise in crossing the BBB/BBTB, although challenges remain in achieving sufficient tumor-specific accumulation. Zhao et al. [31] further emphasized the potential of lipid-based nanoparticles, including liposomes, solid lipid nanoparticles, and nanostructured lipid carriers, which are tailored to overcome the BBB and target glioblastoma stem cells, thereby addressing a critical obstacle in brain tumor therapy. The immunosuppressive nature of the TME also hampers the efficacy of immunotherapies. Multifunctional liposomes capable of remodeling the tumor immune microenvironment (TIME) have been explored to enhance chemoimmunotherapy outcomes. Li et al. [25] reported on liposomes engineered to modulate immune components within the TME, thereby improving therapeutic responses. Additionally, innovative cell-based delivery systems, such as CAR-neutrophils engineered via CRISPR/Cas9, have been developed to deliver TME-responsive nanodrugs specifically to glioblastoma, circumventing immune barriers and reducing inflammation at tumor sites [32].

Nanotechnology-based strategies are central to overcoming these barriers. Ahmadzadeh et al. [33] discussed the development of bio-based, intelligent nanoformulations that are more biocompatible and controllable, capable of interacting with the TME to enhance drug delivery. Similarly, Sun et al. [26] described the use of metal–polyphenol nanoparticles to disassemble dynamically, releasing hyaluronidase precisely at the tumor site to degrade ECM components and improve immune responses. Targeted delivery systems such as antibody–drug conjugates face their own set of challenges, primarily related to tumor penetration. Buyukgolcigezli et al. [34] noted that antibody–drug conjugates limited ability to penetrate deep into the TME restricts their therapeutic potential. Strategies to improve penetration, including reducing toxicity and resistance, are ongoing, with the development of more sophisticated delivery systems being a key focus.

In summary, the solid TME constitutes a multi-layered barrier involving physical structures like the ECM and abnormal vasculature, biological components such as CAFs and immunosuppressive cells, and specialized barriers like the BBB/BBTB. Overcoming these barriers requires a multifaceted approach, integrating nanotechnology, enzymatic ECM degradation, vascular normalization, and immune modulation. Advances in nanocarrier design, such as liposomes, biomimetic nanoparticles, and cell-based delivery systems, show promise in addressing these challenges. Continued research into the dynamic interactions within the TME and the development of innovative strategies are essential for improving drug delivery efficacy and ultimately enhancing therapeutic outcomes in solid tumors.

## 3. Architectural Toolkit: Core Components of Smart Nanoformulations

Designing efficient smart nanoformulations begins with the selection and optimization of foundational materials and core properties. The size, shape, surface charge, and chemical composition of a nanoparticle dictate its initial fate in vivo: its circulation time, its passive accumulation via the EPR effect, and its initial interaction with plasma proteins. This section details these fundamental building blocks—which must be carefully chosen to provide stability, biocompatibility, and drug-loading capacity. Critically, these intrinsic properties directly precondition the formation of the protein corona (Section 4), which in turn influences all subsequent functional layers, including targeting and intracellular fate. Therefore, rational design starts here, with properties engineered not in isolation, but with foresight of the complex biological identity the nanoparticle will acquire. These components work synergistically to address the complex challenges of drug delivery in the TME.

### 3.1. Nanomaterials Properties

These materials, due to their nanoscale size and customizable surface properties, offer significant advantages over traditional chemotherapy, including improved targeting of tumor cells, reduced systemic toxicity, and the ability to overcome multidrug resistance. The integration of nanomaterials into drug delivery systems is transforming cancer therapy by enabling precise delivery of therapeutic agents directly to cancer cells, thereby minimizing damage to healthy tissues.

- Size: The size of nanoparticles is crucial for their biodistribution and clearance. Smaller nanoparticles can penetrate deeper into tumor tissues due to the EPR effect, while larger particles may be cleared more rapidly by the reticuloendothelial system [35,36].
- Shape: The shape of nanoparticles affects their circulation time and cellular uptake. Rod-shaped particles, for example, have been shown to have longer circulation times and better tumor penetration compared to spherical particles [37].
- Surface charge: The zeta potential of nanoparticles influences their interaction with biological membranes and proteins. Positively charged particles may have enhanced cellular uptake but can also lead to increased toxicity, whereas neutral or slightly negative charges are often preferred for prolonged circulation [38].
- Surface chemistry: Functionalization with polyethylene glycol (PEGylation) or targeting ligands can improve the stability and targeting specificity of nanoparticles (Table 3). PEGylation helps evade immune detection, prolonging circulation time, while ligands can direct nanoparticles to specific tumor markers [36,37].
- High drug loading capacity: Nanomaterials can encapsulate a wide range of therapeutic agents, including hydrophilic and hydrophobic drugs, due to their versatile structures such as liposomes, dendrimers, and polymeric nanoparticles [39].
- Controlled release: These materials can be engineered to release drugs in a controlled manner, either passively through diffusion or actively in response to specific stimuli such as pH, temperature, or enzymes present in the TME [40].

**Table 3:** Overview of surface modification strategies in nanoformulations [41].

Modification	Advantage(s)	Limitation(s)	Application(s)
PEGylation	Enhanced solubility Decreased immune recognition Increased circulation	Non-biodegradability Ligand shielding Anti-PEG antibody induction	Stealth liposomes Oncaspar^®^ Doxil^®^
Hydrophobicity tuning	Controlled tissue selectivity via lipid composition Enhanced drug encapsulation	Enhanced reticuloendothelial system uptake (excessive hydrophobicity) Reduced colloidal stability	Organ-targeted SORT systems mRNA lipid nanoparticle
Surface charge modulation	Tumor-triggered charge reversal potential Increased endocytosis (positive charge)	Limited uptake (negative charge) Rapid reticuloendothelial system clearance (positive charge)	Enzyme/pH-triggered charge reversal nanoparticles for tumor penetration
Targeting ligand optimization	Hierarchical targeting capability Increased cellular uptake Receptor-specific binding	Off-target binding in normal tissues Immunogenicity risk	LYTAC/PROTAC delivery FA/RGD dual-targeting systems HER2-targeted nanoparticles

### 3.2. Targeting Mechanisms

The primary mechanisms employed in these systems include passive and active targeting, which are further refined by stimuli-responsive release strategies. These advancements in nanotechnology have shown significant promise in clinical applications, although challenges such as scalability and biocompatibility remain.

#### 3.2.1. Passive Targeting

The use of passive targeting in oncology for targeted drug delivery through nanoformulations is a promising strategy to enhance the efficacy of cancer treatments while minimizing side effects. Passive targeting exploits the unique pathophysiological features of tumor tissues, such as the EPR effect, to deliver nanoparticles loaded with therapeutic agents directly to the tumor site. This approach allows for higher drug concentrations within the TME, reducing systemic exposure and toxicity to healthy tissues. The development of nanoparticle-based drug delivery systems has shown significant potential in improving the therapeutic index of anticancer drugs by enhancing their solubility, stability, and bioavailability.

- EPR effect: Tumors often have leaky vasculature and poor lymphatic drainage, which allows nanoparticles to accumulate more readily in tumor tissues compared to normal tissues. This phenomenon is the cornerstone of passive targeting strategies [42].
- The size, shape, and surface charge of nanoparticles are critical factors that influence their ability to exploit the EPR effect. Optimizing these parameters can enhance the accumulation and retention of nanoparticles in tumor tissues [43].
- Reduced systemic toxicity: By concentrating the drug delivery to the tumor site, passive targeting minimizes the exposure of healthy tissues to toxic chemotherapeutic agents, thereby reducing side effects [44].
- Improved drug efficacy: The increased concentration of drugs at the tumor site can lead to enhanced therapeutic outcomes, potentially improving patient survival rates and reducing the likelihood of cancer recurrence [42].
- Compatibility with various nanocarriers: Passive targeting can be applied to a range of nanocarriers, including liposomes, dendrimers, and polymeric nanoparticles, each offering unique benefits in terms of drug loading and release profiles [45].

#### 3.2.2. Active Targeting

Active targeting in oncology using nanoformulations represents a cutting-edge approach in cancer treatment, aiming to enhance the specificity and efficacy of drug delivery systems. This strategy leverages the unique properties of nanoparticles to deliver therapeutic agents directly to cancer cells, thereby minimizing systemic toxicity and improving therapeutic outcomes. Active targeting involves the use of ligands or antibodies that bind specifically to receptors overexpressed on cancer cells (Table 4), facilitating precise drug delivery. This approach is complemented by passive targeting mechanisms, such as the EPR effect, which allows nanoparticles to accumulate in tumor tissues due to their leaky vasculature. The integration of these targeting strategies into nanoformulations holds significant promise for advancing cancer therapy.

- Ligand conjugation: Active targeting often involves the conjugation of ligands or antibodies to nanoparticles, which bind to specific receptors on cancer cells. This enhances the selectivity of drug delivery, reducing off-target effects and improving drug accumulation in tumors [46].
- Stimuli-responsive systems: These systems release their therapeutic payload in response to specific environmental triggers, such as pH changes or enzymatic activity, which are characteristic of the TME. This ensures that drugs are released precisely at the site of action [47].
- Liposomes and dendrimers: These are commonly used nanocarriers that can be engineered for active targeting. They offer advantages such as improved drug solubility and stability, and can be modified with targeting ligands for enhanced specificity [46].
- Polymeric nanoparticles: These carriers are versatile and can be tailored to deliver a wide range of therapeutic agents. They are often used in conjunction with ligands for active targeting, enhancing drug delivery to cancer cells [47].

**Table 4:** Overview of receptors overexpressed on various cancer cells and their respective ligands [47].

Cancer	Ligand(s)	Receptor
Hepatocellular carcinoma	Galactose and lactobionic acid	Asialoglycoprotein receptor
Glioblastoma	Interleukin13 peptide	Interleukin 13
Lung cancer	Pep-1	Interleukin 4
Glioblastoma and colon	AP1 peptide	Interleukin 4
Pancreatic cancer	Anti-vascular endothelial growth factor mAb	Vascular endothelial growth factor
Prostatic cancer	A10 PSMA Apt, anti-PSMA	PSMA
Hepatocellular carcinoma, cervical cancer, lung, and breast	Biotin	Biotin
Breast cancer	Peptide	LHRH
Lymphoma, hepatocellular carcinoma, lung, and breast	Peptide T22, peptide R, anti-CXCR4 mAb, and LFC131 peptide	Chemokine (CXCR4)
Breast cancer	Tamoxifen, 17 β-estradiol, and estrone	Estrogen
Breast cancer	Trastuzumab Breast anti-HER2 scFv neu peptide (FCDGFYACYADV) KCCYSL (P6.1 peptide)	HER2
Glioblastoma and breast cancer	Transferrin and TfR ligand	Transferrin
Breast, melanoma, lung, glioma, and endothelial	RGD peptide	αvβ3 integrin
Melanoma and breast	Chondroitin sulfate and hyaluronic acid	CD44
Lymphoma	Anti-CD22 mAb	CD22
Prostatic cancer	Anti-CD14 mAb	CD14
Hepatocellular carcinoma, cervical, lung, and breast	Folic acid	Folate

### 3.3. Stimuli-Responsive Materials

**Figure 1:**
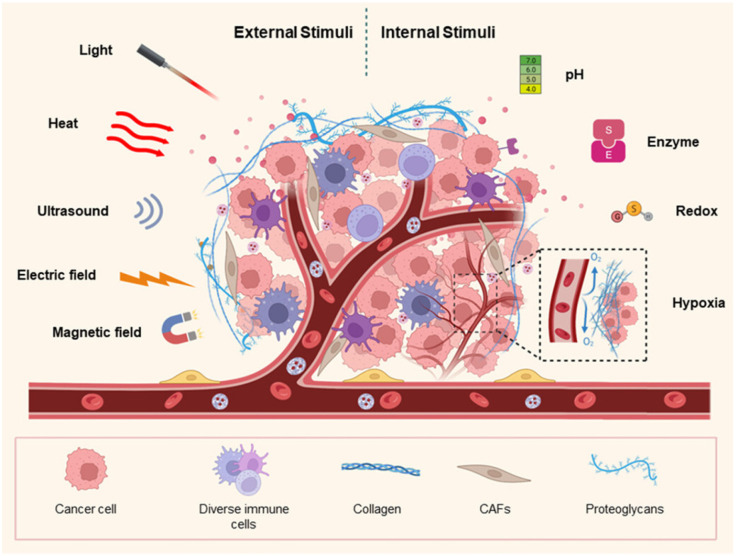
Overview of various stimuli (external and internal) in the formulation of nanocarriers for cancer therapy. Reproduced from [12].

**Figure 2:**
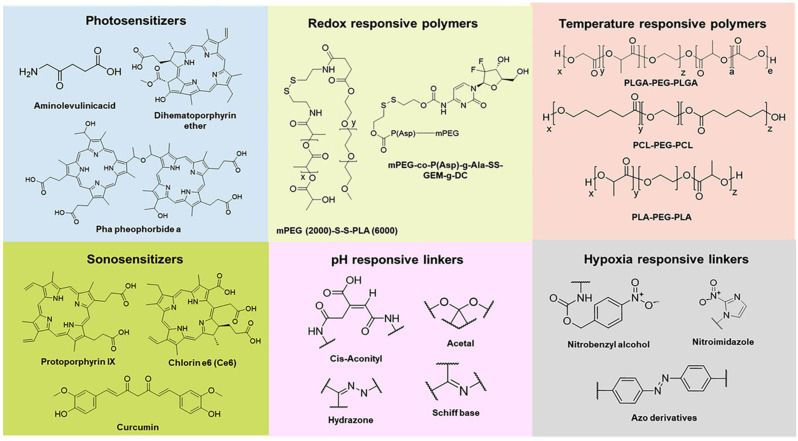
Chemical structure of various materials employed in nanoformulations to provide stimuli responsiveness. Reproduced from [53].

Nanoassemblies and nanoparticles are designed to navigate the complex TME, enhancing drug accumulation at the tumor site and reducing off-target effects [48]. Smart polymers, hydrogels, micelles, dendrimers, and nanogels are some of the forms these materials can take, each offering unique advantages in drug delivery. For instance, hydrogels can swell or shrink in response to environmental changes, while micelles can encapsulate hydrophobic drugs and release them in response to specific stimuli [49]. Smart nanoformulations often utilize the unique characteristics of the TME, such as acidic pH, high levels of glutathione, and overexpressed enzymes, to trigger drug release specifically at the tumor site [50,51]. External stimuli like temperature, light, and magnetic fields can also be employed to control drug release, providing an additional layer of specificity and control [52]. Figure 1 presents various stimuli (external and internal) in the formulation of nanocarriers for cancer therapy. Figure 2 presents chemical structure of various materials employed to provide stimuli responsiveness in nanoformulations. Table 5 presents overview of stimuli-response for targeted drug delivery. Table 6 presents overview of few examples of stimuli-responsive nanoformulations reported for targeted drug delivery.

**Table 5:** Comparative overview of stimuli-response for targeted drug delivery [52,54,55,56,57].

Stimuli	Key Materials	Advantages	Limitations
pH	Hydrazone/imine linkers, DNA i-motifs, histidine rich peptides, Poly(acrylic acid), and chitosan	Autonomous activation broad applicability (endosomes, tumors)	Potential off-target activation in other acidic sites (inflammation) Limited pH gradient between tumor/normal tissue
Redox	Disulfide-conjugated polymers/drugs and disulfide cross-linkers	High specificity due to large extra- and intra-cellular glutathione gradient	Less effective for extracellular release Primarily an intracellular trigger
Enzyme	Ester bonds, polysaccharides (hyaluronic acid), and peptide sequences (MMP substrates)	Catalytic amplification Very high specificity and biological relevance	Potential for immunogenicity of peptide substrates Enzyme levels can be heterogeneous
Reactive oxygen species	Polymers with thioketal/thioether linkers and bilirubin nanoparticles	Can have dual therapeutic effect (drug delivery + reactive oxygen species scavenging) Targets oxidative stress characteristic of inflammation and cancer	Reactive oxygen species levels can be heterogeneous and transient
Hypoxia	Azobenzene, nitroimidazole derivatives, quinone-based systems	Targets a universal feature of solid tumors Allows specific activation in resistant, hypoxic regions	Hypoxic regions are often poorly perfused, limiting nanocarrier delivery The reductive environment can be slow to trigger release
Temperature	Poly(N-isopropylacrylamide) and elastin-like polypeptides	Can be triggered non-invasively with localized hyperthermia Sharp and tunable response	Potential for damage to healthy tissue if heating is not precise Requires external heating equipment
Light	Carbon nanotubes, gold nanoparticles, o-nitrobenzyl groups, and azobenzene	Non-invasive Unparalleled spatiotemporal control (on/off switching)	Potential phototoxicity Limited tissue penetration depth, especially for ultraviolet/visible light
Magnetic field	Superparamagnetic iron oxide nanoparticles embedded in carriers, cobalt ferrite, manganese ferrite, zinc substituted magnetite, and silver–iron oxide composite	Enables dual imaging and therapy Deep tissue penetration	Guidance is limited to accessible sites Potential for non-specific heating Requires specialized equipment
Ultrasound	Nanoemulsions, micelles, liposomes, and tetrahydropyranyl acetal group	Can enhance uptake via sonoporation Deep tissue penetration Non-invasive	Potential for off-target tissue damage if not focused correctly Complex dose–response relationship
Electric field	Conductive polymers, electro-responsive hydrogels, piezoelectric materials	High spatiotemporal precision Can enhance drug permeation (electroporation) Non-thermal, controllable	Limited to superficial or accessible tumors Requires electrode placement or specialized setups Potential tissue damage at high voltages

**Table 6:** Examples for stimuli-responsive nanoformulations for targeted drug delivery.

Stimuli	Ligand/Functional Material	Delivery Material	Nanoformulation	Model/System	Outcome(s)	Ref.
pH	Polyethylenimine modified nanoparticles	Paclitaxel	TPP@paclitaxel-CuTCPP	4 T1 tumor-bearing BALB/c mice	Decrease in drug efflux and eliminated stroma multidrug resistance	[58]
Folate receptor	Folic acid	DNA	lipo-polymersomes	HeLa tumor-bearing nude mice	Enhanced endo/lysosome stroma tumor internalization	[59]
Matrix metalloproteinases (MMP-1 and -2)	Nitric oxide	Doxorubicin	PEGylated mesoporous silica nanoparticles	4 T1 tumor-bearing mice	Improved antitumor efficacy via ECM remodeling	[60]
pH	Hydrophobic interactions	Extremely small iron oxide nanoparticles	pH-sensitive magnetic nanogrenades	HCT116 tumor-bearing mice	Improved theranostic effect for heterogeneous tumors	[61]
Signal regulatory protein-alpha	CD-47	Doxorubicin and gefitinib	Cationized mannan-modified extracellular vesicles	A549 tumor-bearing mice	Extended circulation time and reduced mononuclear phagocyte system clearance	[62]
Membrane type 1-matrix metalloproteinase	cRGDfK	Phosphorylated calcipotriol and doxorubicin	Liposome-linked cRGDfK prodrug	BxPC-3 and HPaSteC cells-bearing mice	Improved hemoperfusion and intravascular tumor delivery	[63]
Glutathione	Disulfide linker	Indocyanine green and methylene blue	Mesoporous silica-iron oxide-gold-P^D^PPA-1	EMT-6 cells-bearing BALB/c mice	Improved r2 relaxation time, eliminated immunosuppressive TME	[64]
Legumain	Programmed death-ligand 1	Doxorubicin	Antiangiogenic peptide (FSEC) and an immune checkpoint blocking peptide (^D^PPA)	4 T1 tumor-bearing BALB/c mice	Decreased vessel density and permeability	[65]
pH	Tumor-penetrating peptide (iRGD)	Paclitaxel	Poly lactic-co-glycolic acid nanoparticles	LS174T xenografts bearing BALB/c mice	Improved delivery across endothelial cell barrier	[66]
Vascular endothelial growth factor receptor 1	Anti-Flt1 peptide	Anti-Flt1 peptide	anti-Flt1 peptide@gold nanoclusters	CAL-27 tumor-bearing BALB/c mice	Enhanced migration inhibitory accumulation effect	[67]
Caspase 3	Ultrasmall superparamagnetic iron oxide nanoparticles	Ac-Asp-Glu-Val-Asp-Cys(StBu)-Lys-CBT substrates	Ultrasmall superparamagnetic iron oxide nanoparticles	HepG2 tumor-bearing mice	Reduced T2 relaxation time and enhanced imaging	[68]
Legumain	Tetrapeptide linker (Ala-Ala-Asn-Leu)	Doxorubicin	Poly(ethylene glycol) micelle	MDA-MB-435 tumor-bearing mice	Enhanced antitumor efficacy without systemic toxicity	[69]
Matrix metalloproteinase-2	mPEG-PVGLIG-IDOi	PEGylated indoleamine-2,3-dioxygenase inhibitor (Epacadostat) and Indocyanine green	mPEG-Pep-IDOi	B16-F10 tumor-bearing BALB/c mice	Enhanced internalization and in with size changeable nanoplatform	[70]
Matrix metalloproteinase-2	Membrane surface P-selectin	Evans blue, paclitaxel and doxorubicin	TM33 peptide-gelatin/oleic acid-tanshinone IIA	BxPC-3 cells-bearing mice	Enhanced tumor permeation by degrading endothelial barriers	[71]
Enzyme (NAD(P)H:quinone oxidoreductase-1)	Quinone linker	Doxorubicin	Trimethyl-locked quinone propionic acid- polycaprolactone-poly(ethylene glycol) micelle	A549-bearing BALB/c nude mice	Improved tumor regression and accumulation	[72]
pH	i-motif, tyrosine kinase 7	Cy5 and doxorubicin	DNA- PEGylated-graphene oxide	T cell line, human acute lymphoblastic leukemia cells-bearing nude mice	Single platform fluorescent imaging and in vivo therapy	[73]
pH	Amine linker	Doxorubicin	graphene oxide nanoparticle with dimethylmaleic anhydride-modified chitosan	In vitro HepG2 cells	Tunable release profile for enhanced uptake	[74]
Lactic acid	Lactate oxidase	Lactate oxidase	Ce-benzenetricarboxylic acid metal–organic framework	HepG2 tumor-bearing BALB/c nude mice	Induced hydrogen peroxide release and production of hydroxyl free radicals	[75]
Adenosine triphosphate (ATP)	Fibers disassembly	Porphyrin	Porphyrin–ATP nanofibers	MCF7 tumor-bearing BALB/c mice	Easier preparation and excellent cellular uptake rate	[76]
ATP	Poly(Asp-Lys)-*b*-Asp	Doxorubicin and camptothecin	Polypeptide-wrapped mesoporous-silica-coated multicolor upconversion nanoparticle	In vitro HeLa cells and MCF-7 cells	Monitoring of real time entrapped drug release	[77]
ATP	Protein-based theranostic agents	Gd(III)/CuS-coloaded BSA	Protein-based theranostic agents/ethylenediamine dextrin	4 T1 tumor-bearing mice	Enhanced imaging resolution and photo thermal efficacy	[78]
ATP	Protamine and DNA scaffold	Doxorubicin	Peptide-fusogenic liposome	MCF-7 tumor-bearing nude mice	Direct delivery and transport of drug to cytosol	[79]
Glutathione	Reduction of Cu(II) to Cu(I)	S-nitrosothiol and cyanine	F127-copper-phloroglucinol metal–organic framework	4 T1 tumor-bearing mice	Enhanced T1 relaxation rate	[80]
Glutathione	Folate receptor and disulfide linker	Doxorubicin and superparamagnetic iron oxide nanoparticles	Folic acid-*β*-cyclodextrin-poly (ε-caprolactone)-dextran star polymer	In vitro HepG2 cells	Enhanced T2 relaxation rate and tumor targeting capacity	[81]
Glutathione	In situ generation of Mn(II)	Bleomycin	Poly(ethylene glycol)-hollow mesoporous manganese dioxide nanoparticles	4 T1 tumor-bearing BALB/c mice	Enhanced T1 relaxation rate, reduced pulmonary toxicity	[82]
Glutathione	Disulfide linker	Extremely small iron oxide nanoparticles and cyclo[Arg-Gly-Asp-d-Tyr-Lys]	Poly(carboxybetaine methacrylate)	U87-MG tumor-bearing mice	Improved targeting specificity and contrasting effect	[83]
Reactive oxygen species	Triphenyl phosphonium	Liposome	Inhibitor of glutamate dehydrogenase 1 (R162) and hydrophobic sonosensitizer (IR780)	4 T1 tumor-bearing mice	Inhibited immune response and tumor immunotherapy	[84]
Reactive oxygen species	1,8-naphthalimide-based fluorescent monomer	Doxorubicin and naphthalimide	Poly(ethylene glycol) micelle	In vitro A549 cells	Showed concentration-dependent cytotoxicity	[85]
Hypoxia	Azo-4,4′ - dicarboxylic acid	(CRISPR) protein 9	Gold nanorods	A549 tumor-bearing nude mice	Milder temperature photothermal therapy	[86]
Hypoxia	Azo linker	Oxaliplatin	Human serum albumin based nanosystem	4T1 tumor-bearing mice	Extended blood circulation and improved accumulation	[87]
Hypoxia	6-(2-nitroimidazole)hexylamine moieties	Doxorubicin and indocyanine green	PEG-b-PPA-NIDH	4 T1 tumor-bearing BALB/c mice	Removed tumor recurrence and repressed distant tumor	[88]

In summary, while the integration of these components offers significant advancements in targeted drug delivery, challenges remain. The complexity of the TME and the need for precise control over drug release and targeting require ongoing research and development. Additionally, issues related to manufacturing scalability, regulatory approval, and long-term biocompatibility must be addressed to translate these innovations from the laboratory to clinical practice.

## 4. Protein Corona in Oncology: Re-Evaluating ‘Stealth’ and Targeting in a Complex Biological Milieu

Upon entering the bloodstream, the engineered nanoparticle surface described in Section 3 is immediately transformed by the adsorption of proteins, forming a dynamic ‘protein corona.’ This corona is not a mere artifact; it becomes the de facto biological interface, overriding the original synthetic design. It determines immune recognition, biodistribution, and circulation half-life. A core translational challenge is that this corona can obscure targeting ligands and alter cellular uptake pathways. Therefore, the ligand engineering strategies developed in Section 5 must be designed with corona formation in mind.

The protein corona has emerged as a pivotal factor influencing the biological identity and targeting efficacy of nanoparticles in oncology (Table 7). Its formation, composition, and subsequent impact on nanoparticle behavior in complex biological milieus have been extensively studied, revealing both challenges and opportunities for precision medicine. The protein corona forms in 2 distinct layers with different biological implications: (i) Soft corona: Consists of low-affinity, rapidly exchanging proteins. This layer is more dynamic and can be displaced during cellular interactions, potentially exposing underlying targeting ligands. (ii) Hard corona: Comprises high-affinity proteins that bind tightly to the nanoparticle surface, creating a relatively stable interface that defines the nanoparticle’s biological identity. This layer is more persistent and primarily determines cellular recognition and fate [89,90].

**Table 7:** Examples for application of protein corona coating on nanoformulations for targeted drug delivery.

Nanoformulation	Protein Corona	Delivery Material	Application	Outcome(s)	Ref.
LinTT1-peptide functionalized liposomes	Human plasma	Doxorubicin and sorafenib	Targeted breast cancer therapy	LinTT1-targeted liposomes co-loaded with doxorubicin and sorafenib enhance anti-metastatic triple negative breast cancer therapy via M2 macrophage uptake	[91]
Molecularly imprinted polymer nanogels	Albumin	Albumin	Refractory cancer cell lines and immune cells	Regulation of cellular uptake by molecularly imprinted polymer nanogels through phosphatidylcholine control	[92]
Bio-inspired liposome (SP-sLip)	Peptide Aβ_1–42_	Doxorubicin	Glioma cells	Enhanced tumor targeting through controlled phosphatidylcholine properties	[93]
Liposome	Human plasma	Liposome–protein complexes	Pancreatic ductal adenocarcinoma	Promoted the cellular internalization of nanoparticles in cancer cells	[94]
Hollow mesoporous silica nanoparticles	Transferrin	Doxorubicin	Redox-controlled and targeted chemotherapy of tumor	A dual-action mechanism: promoting apoptosis while suppressing inflammation	[95]
Gold nanorods	Mouse serum	Chlorin e6 (photosensitizer)	In vitro release of a Chlorin e6 for simultaneous photodynamic and photothermal therapy	Laser irradiation effectively suppressed tumor cell growth over a 19-day period	[96]
Polymeric nanoparticles with carboxyl groups	Anti-CD63-f	Anti-CD63-f	To promote drug delivery	Antibody-functionalized nanoparticles enable targeted delivery to monocyte-derived dendritic cells	[97]
Small interfering RNA-loaded cell-penetrating peptide with IR-780 (NIR dye)	Human serum albumin	Small interfering RNA-loaded cell-penetrating peptide	Synergistic effect of photothermal and RNA interference therapy to prevent lung metastases of breast cancer	Dramatically improves the deep penetration capacity in tumor mass	[98]
Silicon dioxide	Biotin-cystein	Streptavidin	A549 cells	Increase in the circulation time and cellular uptake	[99]
polystyrene nanocarriers modified with Poly(ethylene glycol) or Poly(ethyl ethylene phosphate)	Clusterin proteins (apolipoprotein J)	-	Decrease of non-specific cellular internalization	A specific protein is essential to prevent non-specific cellular binding	[100]
Magnetic nanoparticles	Human serum	-	To enhance internalization and uptake by C4-2B and Panc-1 cancer cells	Improved drug delivery and cell trafficking through the endocytic mechanism	[101]
Silica particles	Human serum	Human serum albumin	To study anticancer effects against HER2 receptor positive SKOV-3 human ovarian cancer cells	Increased the specific association of functionalized particles to SK-OV-3 human ovary cancer cells (to ∼90%)	[102]
Silica nanoparticles	Serum	Doxorubicin and meloxicam	To investigate dual drug loading and release	Antiproliferative effects were reported for silica nanoparticles containing both drugs	[103]
Poly(methacrylic acid) capsules	Human serum	Humanized A33 monoclonal antibody	Targeting ability toward human colon cancer cells	Protein corona increased the interaction with cancer cell receptors	[104]

Surface engineering of nanoparticles is central to modulating the protein corona to enhance targeting performance. According to a recent review on surface engineering strategies, the biological identity of nanoparticles is significantly altered by the formation of a protein corona, which can impede or facilitate targeting depending on its composition [41]. The corona’s formation is an inevitable consequence of nanoparticle exposure to biological fluids, where plasma proteins adsorb onto the nanoparticle surface, creating a new interface that can obscure or modify the intended targeting ligands. This phenomenon complicates the design of ligand-mediated active targeting strategies, as the corona can mask surface functionalities, thereby reducing targeting specificity and efficiency [41].

Corona composition is not static but evolves through a process termed the ‘Vroman effect’ [105]: (i) Initial adsorption: Rapid binding of abundant, high-mobility proteins (e.g., albumin, fibrinogen), (ii) Competitive displacement: These proteins are gradually replaced by higher-affinity but less abundant proteins (e.g., immunoglobulins, complement factors, apolipoproteins), and lastly (iii) Tissue-specific remodeling: Corona composition further adapts as nanoparticles traverse different biological compartments (blood → tissue → cellular interfaces). This temporal evolution means that the corona presented at the target site may differ significantly from that formed immediately upon intravenous administration, complicating targeting predictability [89].

Lipid nanoparticles, widely used in gene delivery and cancer therapy, exemplify the dual role of the protein corona. Structuring lipid nanoparticles to incorporate stealth features, such as PEGylation, aims to reduce protein adsorption and prolong circulation time. However, the corona’s formation can still influence the biodistribution and cellular uptake of these particles, especially in oncological contexts [106]. The complex interplay between nanoparticle design and corona composition underscores the necessity of understanding how corona formation affects targeting in cancer treatment. Progress in nanotechnology has focused on strategies to improve targeted cancer therapy by manipulating the protein corona. Nanoscale biological entities, such as protein-coated nanoparticles, can be engineered to either evade immune recognition or exploit corona components for targeted delivery [107]. For instance, the corona can be tailored to favor the adsorption of specific proteins that facilitate tumor targeting, thereby transforming the corona from a hindrance into an advantage. Nonetheless, the dynamic nature of the corona in vivo presents challenges, as its composition can change over time, affecting the stability and targeting capability of nanomedicines [107].

One significant hurdle presented by the protein corona is its impact on transcytosis across biological barriers, such as the BBB. Studies have shown that corona formation can hinder the transcytosis of transferrin-modified nanoparticles designed for brain tumor targeting [108]. The corona can mask targeting ligands like transferrin, reducing their ability to interact with receptors on endothelial cells, thus impairing delivery to brain tumors. This highlights the importance of designing corona-resistant or corona-structured nanoparticles to maintain targeting functionality in complex biological environments [108]. The influence of the protein corona extends to the ultimate fate of nanomedicines, affecting their circulation, cellular uptake, and clearance. Akhter et al. [109] emphasized that biomolecular interactions with nanomaterials significantly alter their biological identity, impacting biodistribution and therapeutic outcomes. The corona’s composition can lead to opsonization, promoting clearance by the mononuclear phagocyte system, or it can be engineered to avoid such recognition. Strategies to mitigate undesired corona effects include surface modifications that resist protein adsorption or selectively adsorb beneficial proteins [109].

Efforts to avoid or control the inhibitory effects of the protein corona have led to innovative approaches, such as designing corona-inhibiting surface coatings or utilizing specific protein interactions to enhance targeting. For example, creating an opsonin-deficient corona can improve nanoparticle stealth and prolong circulation, thereby increasing tumor accumulation [110]. These approaches aim to preserve the targeting ligands’ accessibility and functionality, which are often compromised by nonspecific protein adsorption. In the context of cancer therapy, artificial engineering of the protein corona has been proposed to improve targeting specificity. By manipulating the bio-nano interface, researchers aim to create a corona that favors tumor-specific interactions while minimizing off-target effects [111]. Such bioengineering strategies involve tailoring the nanoparticle surface to control corona composition, thereby enhancing the therapeutic index of nanomedicines. Furthermore, the analysis and isolation of the protein corona are critical for understanding its composition and functional implications. Advanced analytical techniques enable the characterization of corona proteins, providing insights into how they influence nanoparticle behavior in vitro and in vivo [112]. These insights are essential for designing next-generation nanocarriers with predictable and optimized targeting capabilities.

To control rather than merely mitigate corona effects, innovative engineering approaches include: (i) Pre-formed coronas: Incubating nanoparticles with selected proteins (e.g., human serum albumin, transferrin) prior to administration to create a predetermined, functional corona that enhances targeting or stealth properties. (ii) Artificial coronas: Synthetically designing nanoparticle surfaces with biomimetic polymers or peptides that mimic natural dysopsonins or present ‘self’ markers (e.g., CD47 mimetics) to evade immune recognition. (iii) Corona–ligand integration: Designing targeting ligands that either remain accessible despite corona formation or are activated specifically within the TME (e.g., by enzymatic cleavage of a protective corona) [113,114].

Corona formation often masks conjugated targeting ligands, reducing or abolishing their specificity. Strategies to preserve ligand function include: (i) Topographical engineering: Positioning ligands on polymeric brushes or spacers that extend beyond the corona layer. (ii) Stimuli-responsive shielding: Using labile linkers or cloak polymers that detach in response to TME cues (e.g., pH, enzymes), exposing ligands only at the tumor site. (iii) Ligand–corona synergy: Selecting ligands that cooperate with adsorbed proteins to enhance targeting (e.g., transferrin-conjugated nanoparticles benefiting from endogenous transferrin corona) [115,116].

Protein corona formation directly impacts clinical translation by altering: (i) Pharmacokinetics: Corona composition influences circulation time, clearance routes, and organ accumulation. (ii) Targeting fidelity: Ligand masking can reduce tumor specificity, increasing off-target effects. (iii) Batch-to-batch variability: Differences in corona formation due to variations in nanoparticle synthesis or patient serum composition can affect therapeutic consistency. Personalized approaches, such as pre-screening patient serum to predict corona formation and tailoring nanoparticle design accordingly, may enhance clinical predictability [117].

In summary, the protein corona plays a complex and dual role in oncology nanomedicine. While it can hinder targeted delivery by masking functional ligands and promoting clearance, strategic surface modifications and bioengineering approaches can harness or mitigate its effects. The evolving understanding of corona formation and its biological implications is vital for re-evaluating the concept of ‘stealth’ nanoparticles and developing more effective, targeted nanotherapies for cancer. Future research focusing on precise control and characterization of the corona will be instrumental in translating nanomedicine from bench to bedside, ensuring that nanoparticle-based therapies achieve their full potential in complex biological environments.

## 5. Ligand Engineering for Cancer Cell Targeting: Beyond Monovalent Binding

Once a nanoparticle’s systemic behavior and interfacial identity are managed (previously discussed in Section 3 and Section 4), precision delivery requires active molecular recognition. Ligand engineering conjugates targeting moieties (e.g., antibodies, peptides, aptamers) to the nanoparticle surface to bind overexpressed receptors on cancer cells. However, this is not a simple conjugation step. The ligands must be selected and presented to remain accessible and functional within the context of the protein corona (Section 4). Furthermore, mere binding is insufficient; the choice and architecture of the ligand (monovalent, multivalent, bispecific) directly influence the mechanism and efficiency of cellular internalization (Section 6), setting the stage for the critical next phase: intracellular trafficking. This section explores advanced ligand designs that provide robust, specific binding in vivo, creating the necessary molecular handshake to initiate the final delivery process.

Ligand engineering for cancer cell targeting has evolved significantly beyond the traditional monovalent binding strategies, incorporating multivalent and bispecific approaches to enhance specificity, affinity, and therapeutic efficacy (Table 8). The recent literature underscored a shift towards sophisticated design principles that leverage multivalency, bispecificity, and structural optimization to overcome limitations associated with monovalent ligands, such as insufficient binding avidity and off-target effects [118,119].

Monovalent ligands are ideal when targeting a highly overexpressed, homogeneous receptor population with high intrinsic affinity. They minimize steric hindrance and are suitable for well-characterized, stable antigen–ligand pairs [118,119]. Multivalent ligands are preferred when: Receptor expression is moderate or heterogeneous, increased binding avidity is needed to enhance cellular uptake and retention, and lastly spatial clustering of receptors (e.g., lipid rafts) can be exploited for stronger adhesion and signaling [120,121].

**Table 8:** Evolution of ligand engineering strategies for cancer targeting.

Targeting Strategy	Key Principle	Example Ligands and Constructs	Advantages	Limitations	Ref.
Monovalent binding	Single ligand–receptor interaction (‘key-and-lock’)	Monoclonal antibodies (e.g., trastuzumab) Small molecules (e.g., folic acid) Short peptides (e.g., RGD)	High specificity for a single epitope Well-established conjugation chemistry	Limited binding avidity Susceptible to receptor heterogeneity Lower cellular internalization efficiency	[118,119]
Multivalent binding	Simultaneous engagement of multiple receptors to increase binding strength (avidity)	Nanoparticles with high-density ligand coatings Spatially patterned ligand arrays Aptamer clusters	Enhanced cellular uptake and retention Overcomes moderate receptor affinity More robust against single receptor downregulation	Optimal ligand density is target-specific Risk of non-specific binding if overused Complex synthesis and characterization	[120,121]
Bispecific/multispecific targeting	Engages 2 or more different tumor-associated antigens or immune receptors simultaneously	Bispecific T-cell engagers Trispecific antibodies (e.g., targeting EGFR, PD-L1) Dual-targeted nanoparticles (e.g., RGD + folic acid)	Overcomes tumor heterogeneity Synergistic signaling blockade Recruits immune effector cells	Increased complexity and potential immunogenicity Balancing affinity for different targets is challenging More difficult manufacturing and regulatory path	[122,123,124]
Advanced modalities and architectures	Utilizes non-antibody scaffolds or novel conjugation methods for improved properties	Aptamers (e.g., AS1411) Affibodies and DARPins Antibody–drug conjugates on cell surfaces (e.g., CAR-natural killer) Programmable oligonucleotide constructs (e.g., PROTACs)	Small size for improved tumor penetration High stability and potential for chemical synthesis Enables novel mechanisms of action (e.g., protein degradation)	Potential for rapid renal clearance Immature regulatory framework for some modalities Off-target effects need careful evaluation	[125,126,127]

One prominent strategy involves the development of multivalent ligands that can engage multiple receptors or epitopes simultaneously, thereby increasing binding strength and cellular internalization efficiency. Makhani et al. [120] highlighted the importance of parameters such as nanoparticle size, shape, ligand density, and receptor density in modulating multivalent adhesion and internalization. They emphasized that experimental and computational studies should be integrated to optimize these parameters, ultimately leading to more potent nanoparticle-based therapeutics. Similarly, Xu et al. [121] demonstrated that spatially patterned multivalent ligand arrays can significantly enhance targeting capabilities, particularly when designed to patch to receptor clusters like epithelial cell adhesion molecules. There findings suggested that precise spatial arrangement and valency control are critical for maximizing bioavailability and cell growth modulation.

Optimal ligand density varies by target and nanoparticle size. Excessive density may cause steric hindrance, reduce binding efficiency, or induce non-specific aggregation [128]. Controlled inter-ligand spacing (e.g., via spacers like PEG) can improve accessibility to receptors and mimic natural ligand–receptor interactions. Site-specific conjugation (e.g., via engineered cysteine residues or click chemistry) preserves ligand orientation and functionality, enhancing binding specificity and avidity [129].

Further, high-affinity or high-density ligand systems may saturate target receptors, leading to reduced internalization and potential feedback downregulation. Off-target binding can occur in tissues expressing low levels of the target antigen, necessitating careful in vivo validation and the use of activatable or masked ligands [130].

Beyond simple multivalency, the design of bispecific and trispecific antibodies has gained traction as a means to simultaneously target multiple tumor-associated antigens or immune checkpoints. Li et al. [122] described the construction of a bispecific antibody, PT886, targeting both CLDN18.2 and CD47, which employs innovative platforms such as PACbody™ and SPECpair™ to achieve robust anti-tumor activity. This dual targeting not only enhances tumor cell recognition but also engages immune effector mechanisms like macrophage-mediated phagocytosis. Similarly, Bogen et al. [123] reported the generation of trispecific antibodies based on a common light chain architecture, capable of engaging epidermal growth factor receptor (EGFR), programmed death-ligand 1 (PD-L1), and other targets, demonstrating synergistic effects that surpass monovalent or bivalent counterparts.

The concept of multivalency extends to immune cell engagement as well. Boje et al. [124] explored how antibody architecture and paratope valency influence natural killer cell-mediated cytotoxicity. Their work showed that bivalent natural killer ell engagers targeting EGFR and NKp30 outperform monovalent constructs, emphasizing that valency and architecture are crucial for effective immune activation. Similarly, Lim et al. [127] introduced a surface-engineered natural killer platform that incorporates antibody–drug conjugates onto immune cells, enhancing tumor targeting while reducing off-target effects. These studies collectively demonstrate that increasing valency and optimizing antibody architecture can significantly improve therapeutic outcomes.

In addition to protein-based ligands, alternative modalities such as aptamers, antibody mimetics, and engineered peptides are being explored for their multivalent potential. Shao et al. [125] introduced programmable oligonucleotide PROTACs capable of targeting DNA-binding proteins like lymphoid enhancer-binding factor 1 and erythroblast transformation-specific-related gene, illustrating the versatility of nucleic acid-based multivalent constructs in degrading oncogenic transcription factors. Similarly, Zamani et al. [126] develop polymer-based PD-L1 mimetics (iBodies) that function as potent immune checkpoint inhibitors, leveraging multivalent polymer conjugation to enhance binding avidity.

Structural and computational approaches also play a vital role in ligand engineering. Cao et al. [131] utilized in silico methods, including molecular docking and dynamics simulations, to identify inhibitors targeting the DNA-binding domain of estrogen receptor alpha, providing a framework for rational design of high-affinity ligands. Such approaches facilitate the optimization of ligand-receptor interactions, especially when combined with multivalent design principles. Furthermore, innovative strategies such as ligand conjugation to extracellular vesicles are gaining attention. Pan et al. [132] developed quantitative methodologies to evaluate ligand conjugation efficiency on extracellular vesicles, aiming to enhance their targeting capabilities through multivalent ligand display. This approach underscores the importance of ligand density and spatial arrangement in achieving effective targeting. In the context of immune checkpoint blockade, the development of bispecific and multivalent agents has shown promise. Zamani et al. [126] reported polymeric PD-L1 mimetics that exhibit high potency, illustrating how multivalent display can improve immune evasion strategies. Similarly, the work by Zhao et al. [133] on lysosomal degradation of PD-L1 via a meso peptide highlights the potential of modular, ligand-based strategies to modulate immune checkpoints effectively.

In summary, the existing research indicated that ligand engineering for cancer targeting is transitioning from monovalent to multivalent and multispecific constructs. These advances leverage structural optimization, spatial arrangement, and combinatorial targeting to enhance binding avidity, specificity, and therapeutic efficacy. The integration of experimental and computational methodologies continues to drive innovation, enabling the rational design of next-generation ligands that can effectively navigate the complex TME and immune landscape. This evolution beyond monovalent binding underscores a paradigm shift towards more sophisticated, multivalent strategies that hold promise for improved cancer diagnostics and therapeutics.

## 6. Intracellular Trafficking and Organelle-Specific Targeting in Cancer Therapy

Successful ligand-mediated binding (Section 5) triggers cellular internalization, but this often leads to entrapment and degradation in the endolysosomal system—a major cause of therapeutic failure. Therefore, the final, critical tier of nanoformulation design is programming the intracellular journey. Building upon the targeting specificity achieved in the previous stage, this section focuses on engineering nanoformulations to escape endosomes, evade efflux pumps, and navigate to specific subcellular organelles (e.g., mitochondria, nucleus, endoplasmic reticulum). This ‘third level’ of targeting ensures the therapeutic payload is delivered intact to its site of action, maximizing efficacy against drug-resistant cells and enabling novel mechanisms like DNA damage or disruption of metabolic pathways. The integration of stimuli-responsive materials (from the core toolkit) with trafficking peptides represents the culmination of the design pipeline, translating extracellular targeting into intracellular therapeutic action.

Intracellular trafficking and organelle-specific targeting represent critical frontiers in the development of advanced cancer therapies, aiming to enhance drug efficacy and overcome challenges associated with traditional treatments like surgical resection, chemotherapy, and radiation therapy [134]. By precisely delivering therapeutic agents to specific subcellular locations, researchers can maximize their impact while minimizing off-target effects (Table 9).

**Table 9:** Intracellular trafficking pathways and organelle-specific targeting strategies.

Target/Process	Challenge	Nanoformulation Strategy	Mechanism of Action/Functional Component	Therapeutic Outcome	Ref.
Endosomal escape	Entrapment and degradation within endolysosomal compartments	Proton-sponge polymers (e.g., PEI) Fusogenic peptides Photo-induced disruption (e.g., with photosensitizers)	Buffers endosomal pH, causing osmotic swelling and rupture Fuses with endosomal membrane Generates reactive oxygen species or heat to disrupt membrane	Enhanced cytosolic delivery of nucleic acids (siRNA, DNA) and drugs Increasing bioavailability	[135]
Lysosomes	Degradative environment deactivates many therapeutics	Lysosome-targeting nanoparticles Lysosomotropic agents	Selective drug release within lysosomes to exploit acidic pH and enzymes Disrupts lysosomal membrane to induce cell death (lysosomal membrane permeabilization)	Induction of lysosome-dependent cell death Effective for enzyme-activated prodrugs	[136]
Mitochondria	Low natural accumulation of nanocarriers; requires crossing multiple membranes	Triphenylphosphonium conjugation Mitochondria-penetrating peptides DEKK peptide	Utilizes the strong negative mitochondrial membrane potential for targeting Enables direct translocation across mitochondrial membranes	Induction of apoptosis via cytochrome c release Disruption of energy metabolism Enhanced photodynamic therapy	[137,138]
Nucleus	The nuclear envelope and pore complexes restrict access	Nuclear localization signal peptides (e.g., from SV40 T-antigen) Nuclear localization signa conjugated nanocarriers	Binds to importin proteins, facilitating active transport through the nuclear pore complex	Efficient delivery of gene therapies (DNA, CRISPR/Cas9) and radiosensitizers directly to the genetic material	[138]
Golgi apparatus	Targeting a central hub for trafficking and signaling without complete cellular shutdown	Cycling molecular assemblies Small molecule inhibitors (e.g., targeting GOLGA7)	Targets organelle-specific enzyme activities Disrupts specific protein trafficking pathways (e.g., NRAS to plasma membrane)	Interference with critical pro-tumorigenic signaling and protein secretion	[139,140]
Endoplasmic reticulum	Inducing endoplasmic reticulum stress for apoptosis	Endoplasmic reticulum targeting peptides (e.g., ER-12) Nanoparticles that perturb protein folding	Accumulates in the endoplasmic reticulum lumen, disrupting calcium homeostasis and protein folding	Potent induction of endoplasmic reticulum stress and subsequent apoptosis, particularly effective in secretory cells	[141]
General intracellular trafficking	Insufficient cellular uptake and poor subcellular distribution	Cell-penetrating peptides (e.g., hendeca-arginine R11) Bioelectronic approaches (alternating current)	Promotes energy-independent cellular uptake via various endocytic pathways Overcomes endosomal entrapment in drug-resistant cells	Improved overall intracellular bioavailability of therapeutics and overcoming of resistance mechanisms	[142,143]

One fundamental aspect of intracellular targeting is the uptake and subsequent trafficking of therapeutic agents within cancer cells. Nanomedicines are frequently utilized for this purpose, with their entry primarily occurring through endocytosis pathways, including clathrin- and caveolae-mediated mechanisms [143]. These pathways involve specific effector molecules, whose expression differences between normal and tumor cells can be exploited for targeted delivery. Understanding the intracellular trafficking route of endocytosis vesicles is crucial for elucidating anti-tumor mechanisms and designing highly efficacious, cancer-targeted nanomedicines [143]. For instance, hendeca-Arginine (R11) nanocarriers have been developed for targeted gene delivery to bladder cancer, promoting DNA accumulation via the clathrin-independent endocytosis pathway and directing intracellular trafficking of delivered DNA to nonlysosome-localized regions, even enabling intercellular transport [142].

The concept of ‘third level drug targeting’ directly within intracellular locations is emerging as a strategy to significantly enhance treatment efficiency [137]. This approach involves using small molecules, peptides, pH-sensitive liposomes, and other nanoformulations [137]. Targeted liposomes, such as ultra-high field magnetic resonance imaging activatable thermosensitive liposomes, can specifically increase the intracellular accumulation of cytotoxic drugs like doxorubicin in cells, as demonstrated in targeting transmembrane metalloprotease-disintegrin (ADAM8) in triple-negative breast cancer cells [144]. Dendrimer-based nanoconjugates also offer potential in cancer photodynamic therapy, with research focusing on their chemical design, mechanism of action, and intracellular trafficking within cancer cells [145].

Organelle-specific targeting is a powerful strategy to interfere with critical cellular functions in cancer. The Golgi apparatus, a vital organelle for intracellular trafficking and signaling, orchestrating protein and lipid sorting, has garnered attention as a therapeutic target [140,146]. Abnormal Golgi genes and proteins are implicated in carcinogenesis, making it a promising, albeit challenging, target [146]. Novel therapeutic strategies, such as cycling molecular assemblies, show potential for selective Golgi disruption by targeting organelle-specific enzyme activities [140]. Furthermore, the protein GOLGA7 has been identified as essential for NRAS trafficking from the Golgi to the plasma membrane, and its depletion can block NRAS translocation, highlighting a specific trafficking pathway within the Golgi network that could be therapeutically exploited [139].

Lysosomes are another critical component of the inner membrane system, involved in macromolecular degradation, antigen presentation, and even regulating hematopoietic stem cells in acute myeloid leukemia progression, making them a potential therapeutic target [147]. However, for many nanomedicines, lysosomal escape is crucial. Studies have developed nanoparticles, such as folic acid@triphenylamino-phenylaniline zinc phthalocyanine@upconversion nanoparticles, that utilize lysosomal escape mechanisms in conjunction with mitochondria targeting to enhance antitumor efficacy, enabling real-time visualization of their subcellular localization and intracellular trafficking via upconversion luminescence imaging. Mitochondria targeting is increasingly recognized for boosting therapeutic outcomes [138].

Addressing challenges such as chemotherapy resistance and endosomal entrapment, which are controlled by intracellular trafficking processes, is paramount for treatment success [136]. Bioelectronic approaches, involving the application of alternating current, have shown promise in tackling these issues by killing drug-resistant cancer cell lines and overcoming endosomal entrapment [136]. Insufficient cellular uptake and poor targeting abilities remain significant hurdles for nanotechnology in nucleic acid-based cancer therapy, despite its transformative potential [148]. Beyond organelle targeting, modulating specific protein trafficking and cellular signaling pathways offers additional therapeutic avenues. Cyclophilin A, for instance, plays critical roles in protein folding, trafficking, assembly, and cell signaling, presenting a promising target in cancer therapy [141]. EGFR signaling pathways and their intracellular trafficking are also intensely studied, providing insights into mechanisms of inhibitor resistance and guiding the development of novel EGFR-targeted cancer therapies [149].

While adoptive cell transfer, including genetically engineered T cells like CAR T cells and TCR-modified T cells, has shown clinical benefits in treating malignant tumors [134], their effectiveness can be hampered by factors like impaired trafficking of immune cells to solid tumors and tumor evasion mechanisms [150]. Targeting specific protein isoforms, such as Class I, II, and III PI3Ks, also offers potential combination treatment strategies due to their roles in tumor biology [151]. The nucleolin-binding G-quadruplex AS1411 aptamer, when linked to DNA nanostructures, can divert its traffic inside cancer cells, thereby improving its therapeutic efficacy through selective cancer-targeting and anti-tumor activity [135]. Furthermore, the inhibition of exosome biogenesis, release, and uptake, which involves intricate intracellular processing, represents a potential anticancer approach by modulating extracellular vesicle communication [152]. New molecular targets are continuously being identified, such as epithelial membrane protein 2 for lung cancer, paving the way for targeted antibody–drug conjugates [153].

In summary, leveraging the intricate mechanisms of intracellular trafficking and organelle-specific targeting is fundamental to developing more effective cancer therapies. By understanding and manipulating endocytosis pathways, navigating subcellular destinations like lysosomes, Golgi, and mitochondria, and employing sophisticated nanocarriers and bioelectronic strategies, researchers are poised to overcome drug resistance and enhance treatment precision, ultimately improving patient outcomes.

## 7. Overcoming the Stromal Barrier: Nano-Strategies for Tumor Penetration

Overcoming the stromal barrier remains a critical challenge in enhancing the efficacy of cancer therapies, particularly in desmoplastic tumors such as pancreatic and breast cancers. The dense ECM and the complex TME serve as formidable physical and immunological barriers that impede drug delivery, hinder immune cell infiltration, and contribute to therapeutic resistance. Recent advances in nanotechnology and nano-strategies have shown promising potential in addressing these obstacles by facilitating deeper tumor penetration and modulating the tumor stroma (Table 10).

**Table 10:** Nanoformulation strategies to overcome the stromal barrier in solid tumors.

Strategy	Approach	Mechanism of Action	Example(s)	Outcome(s)	Ref.
ECM degradation and remodeling	Enzymatic degradation	Directly breaks down dense ECM components (e.g., hyaluronan, collagen) to reduce physical barriers	Hyaluronidase-modified nanoparticles Collagenase-loaded nanocarriers	Enhanced nanoparticle and drug penetration Reduced interstitial fluid pressure Improved immune cell infiltration	[26,154]
	Pharmacologic modulation	Uses drugs to inhibit ECM production or induce its degradation	Losartan-loaded responsive nanosystems Captopril-containing nanoparticles	Depletion of stromal collagen Improved perfusion and drug delivery	[155,156]
Stromal cell targeting	Targeting cancer-associated fibroblasts (CAFs)	Depletes or reprograms pro-tumorigenic CAFs to a quiescent or anti-tumorigenic state	Biomimetic liposomes targeting FAP Dendritic polymers for metabolic targeting of CAFs	Reduced ECM deposition Disruption of tumor-stroma crosstalk Enhanced chemotherapy sensitivity	[28,157]
Physical disruption	Sonodynamic therapy	Uses ultrasound to activate nano-sonosensitizers, generating reactive oxygen species that disrupt stromal components	Tin monosulfide nano-sonosensitizers	Transient disruption of stromal architecture Synergistic antitumor effect with immune activation	[158]
	Photothermal therapy	Localized heat from nanoparticles (e.g., gold, polydopamine) causes thermal ablation of stroma	Biomimetic mesoporous polydopamine nanoparticles Mitochondria-targeting gold nanoparticles	Physical loosening of the ECM Induces immunogenic cell death	[159,160]
	Magnetic hypothermia	Localized heat from superparamagnetic nanoparticles under an alternating magnetic field causes thermal ablation of stroma and modulates tumor vasculature	Superparamagnetic iron oxide nanoparticles (e.g., for glioma, breast cancer) Carbothermal treated iron oxide Galladium doped maghemite	Physical loosening of the ECM Induced immunogenic cell death Enhanced perfusion and drug penetration	[57]
Multimodal and biomimetic approaches	Combined stromal and immune modulation	Simultaneously targets ECM and reprograms the immunosuppressive microenvironment	Dual-engine nanodisruptors (e.g., Losartan + Radix Hedysari polysaccharide) Macrophage membrane-coated nanoplatforms	Enhanced nanoparticle penetration Increased T-cell infiltration Synergy with immunotherapy	[155,156]
	Ultrasound-triggered gas release	Uses ultrasound to trigger nitric oxide release from nanocarriers, inducing vasodilation and modulating stroma	Ultrasound-responsive nitric oxide releasing nanoparticles	Improved tumor perfusion Enhanced delivery of co-administered therapeutics	[161]

A significant focus has been on understanding the composition and role of the tumor stroma in impeding therapeutic efficacy. Mortezaee [29] highlighted that in pancreatic cancer, the dense stroma not only acts as a physical barrier but also fosters an immunosuppressive TME, leading to ‘cold’ tumors that respond poorly to immunotherapy. The interplay between cancer stem cells and the stromal components further complicates treatment, emphasizing the need for strategies that can disrupt this barrier to improve immune responses. One promising approach involves enzymatic degradation of ECM components to facilitate drug and immune cell penetration. He et al. [154] demonstrated the use of hyaluronidase-modified nanoparticles combined with photothermal therapy delivered via dissolving microneedles, effectively degrading hyaluronic acid in the tumor stroma. This cascade degradation not only enhances the penetration of therapeutic agents but also synergizes with immune activation, providing a platform for melanoma treatment and potentially applicable to other desmoplastic tumors.

Similarly, Chen et al. [155] explored the use of captopril, an angiotensin-converting enzyme inhibitor, to deplete the ECM in pancreatic tumors. Their study showed that targeting the overexpressed ECM components could significantly improve drug penetration, addressing the desmoplastic nature of pancreatic cancer. This approach underscored the importance of stromal remodeling in overcoming physical barriers to therapy. Beyond enzymatic degradation, nanomedicine strategies have been designed to actively target and modulate stromal components. Zhang et al. [157] developed dendritic polymer-based nanomedicines that remodel the tumor stroma, reducing ECM density and enhancing immune cell infiltration. Their work demonstrated that alleviating the ECM barrier could synergize with immune checkpoint blockade, such as anti-PD-1 therapy, leading to reduced tumor burden and metastasis. This indicates that nanomedicines capable of metabolic targeting of CAFs and ECM components can effectively overcome stromal barriers and potentiate immunotherapy.

In addition to ECM degradation, strategies that induce stromal disruption through physical or chemical means have been explored. Li et al. [158] utilized tin monosulfide nanoparticles as nano-sonosensitizers to generate reactive oxygen species via sonodynamic therapy. Their denaturation-and-penetration strategy effectively overcame the stromal barrier in triple-negative breast cancer, simultaneously enhancing sonodynamic therapy efficacy and antitumor immunity. This approach exemplifies how physical stimuli, such as ultrasound, can be harnessed to transiently disrupt stromal barriers, facilitating deeper therapeutic penetration [158]. Photothermal therapy has also been employed to modulate the tumor microenvironment. Huang et al. [159] reported the use of biomimetic mesoporous polydopamine nanoparticles that combine tumor targeting, photothermal therapy, and autophagy blocking. Their work highlighted the challenge of synergistically manipulating autophagy and stromal barriers to improve cancer cell killing. Similarly, Meng et al. [160] developed mitochondria-targeting gold nanoparticles that, upon near-infrared irradiation, induce localized chemo-photothermal effects, disrupting stromal components and enhancing treatment efficacy in pancreatic cancer models.

Targeting CAFs and their secreted ECM components has gained considerable attention. López-Estévez et al. [28] emphasized the importance of personalized nanomedicine strategies that can access and modulate intracellular and stromal barriers. Zhang et al. [157] and Yang et al. [156] further demonstrated that nanomedicines designed to metabolically target CAFs or deliver ECM-degrading drugs like losartan can significantly improve tumor penetration. Yang et al. [156] fabricated a responsive nanosystem that releases losartan in the acidic TME, effectively breaking down ECM barriers and enhancing immunotherapeutic delivery.

Innovative biomimetic nanoplatforms have been developed to address stromal barriers while simultaneously modulating immune responses. Chen et al. [162] created a biomimetic nanoplatform combining photothermal therapy with CAF modulation, revealing that residual tumor cells could secrete tumor growth factor-β-rich exosomes, promoting ECM deposition and resistance. To counteract this, Yang et al. [163] designed a dual-engine nanodisruptor encapsulating losartan and Radix Hedysari polysaccharide within a macrophage membrane-coated framework, effectively overcoming stromal and immune resistance in hepatocellular carcinoma. Emerging physical modalities such as ultrasound-mediated piezocatalysis have also been employed to enhance stromal disruption. Song et al. [161] demonstrated that ultrasound-triggered nitric oxide release could augment targeted immunotherapy in pancreatic cancer, providing a non-invasive method to transiently modulate the tumor microenvironment and improve drug delivery.

Overall, these studies underscored that overcoming the stromal barrier requires a multifaceted approach combining enzymatic degradation, physical disruption, targeted stromal modulation, and nanomedicine-based delivery systems. The integration of these strategies aims to facilitate deeper tumor penetration, reprogram the immunosuppressive TME, and ultimately improve therapeutic outcomes. As López-Estévez et al. [28] and Jang et al. [164] suggested, future directions should focus on personalized nanomedicine platforms and innovative delivery systems capable of navigating complex biological barriers, including the ECM and BBB, to realize the full potential of nanotechnology in overcoming stromal barriers in cancer therapy. These approaches not only enhance drug and immune cell penetration but also remodel the TME to favor therapeutic efficacy, paving the way for more effective treatments against traditionally resistant desmoplastic tumors.

## 8. Endogenous Stimuli-Responsive Systems: Engineering Specificity Within the Tumor Microenvironment

Endogenous stimuli-responsive nanoformulations are engineered to exploit the unique characteristics of the TME for targeted drug delivery and enhanced therapeutic efficacy. These nanoformulations are designed to respond to specific internal stimuli such as pH, redox potential, enzymes, reactive oxygen species, and hypoxia present in the TME, allowing for precise drug release and minimizing damage to healthy tissues (Table 11). The following sections delve into the engineering of specificity within the TME using endogenous stimuli-responsive nanosystems. This section serves as case-study expansion of Section 3.

**Table 11:** Overview of endogenous stimuli-responsive nanoformulation systems.

Stimulus	Advantages	Limitations	Responsive Biomaterials/Moieties	Example(s)	Ref.
pH	Autonomous, ‘self-triggered’ activation Broad applicability (acidic TME, endosomes) Simple chemical design for acid-labile linkers	Limited pH gradient between tumor and normal tissue Potential off-target activation in other acidic sites (e.g., inflammation, stomach) Heterogeneous intratumoral pH distribution	Poly(acrylic acid), chitosan Hydrazone, acetal, vinyl ether linkers Histidine-rich peptides	T12-RBCM@CM nanoparticles (pH-triggered MTX release) PEPCA@SPA/Fe(III) nanoassemblies	[165,166]
Redox (glutathione)	High specificity due to large intracellular/extracellular glutathione gradient (100 to 1000:1) Effective for intracellular targeting and release	Primarily an intracellular trigger; less effective for extracellular drug action Glutathione levels can vary between cancer types Potential interference with the cell’s native redox balance	Disulfide bonds (-S-S-) as cross-linkers or conjugated to drugs Selenium-containing polymers	RPSSD@IR780/Docetaxel nanoparticles (Redox-triggered docetaxel release)	[167]
Enzymes (e.g., MMPs, Cathepsins)	Very high biological specificity and relevance Catalytic amplification (one enzyme cleaves many bonds) Can be designed for tissue-specific enzyme profiles	Enzyme expression levels are highly heterogeneous between and within tumors Potential for immunogenicity of peptide substrates Enzyme activity can be inhibited in the TME	Peptide substrates (e.g., GPLGVRG for MMPs) Ester bonds (for esterases) Polysaccharides (e.g., hyaluronic acid for hyaluronidase)	Methacrylated hyaluronic acid hydrogel (enzyme-triggered cisplatin/PRMT5-short hairpin RNA release) Vitamin D-based micelles (esterase-triggered paclitaxel release)	[168,169]
Reactive oxygen species	Targets a hallmark of cancer (oxidative stress) Can be combined with therapies that further increase reactive oxygen species (e.g., radiotherapy) Some materials offer dual therapeutic effect (drug delivery + reactive oxygen species scavenging)	Reactive oxygen species levels can be transient and heterogeneous High reactive oxygen species can lead to undesired pro-tumor signaling May require combination with other triggers for sufficient specificity	Thioketal linkers Boronic ester/acid derivatives Selenium/tellurium-containing polymers	Chitooligosaccharide nanoparticles (reactive oxygen species triggered curcumin release) Curcumin@biotin/PE nanoformulation	[170,171]
Hypoxia	Targets a universal feature of most solid tumors Allows for specific activation in the most resistant, hard-to-treat tumor regions	Hypoxic regions are often poorly perfused, limiting nanocarrier delivery The reductive environment can be slow to trigger release	Azobenzene groups Nitroimidazole derivatives Quinone-based systems	Poly(ethylene glycol)-phthalic acid-nitroimidazole nanomicelles Azo-linker-based nanoparticles (RP/CA/PHNPs)	[172,173]

### 8.1. pH

Tumor tissues often exhibit an acidic microenvironment compared to normal tissues. pH-sensitive nanoparticles are designed to remain stable at physiological pH but release their payload in the acidic conditions of the TME, enhancing drug delivery specificity [174,175]. These systems can improve the retention and accumulation of anticancer drugs in tumor tissues, thereby increasing therapeutic efficacy [174,176].

**Figure 3:**
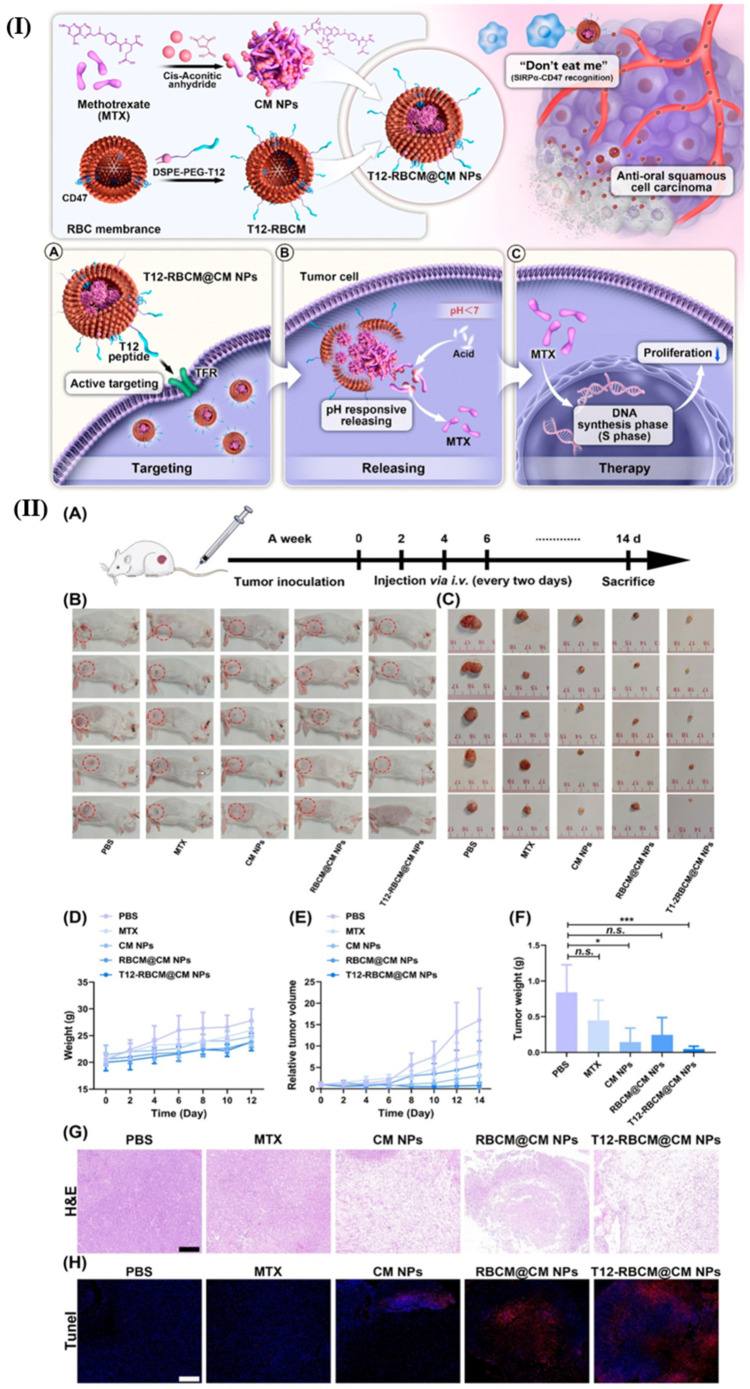
(**I**) Schematic of a pH-responsive methotrexate nanoprodrug (T12-RBCM@CM nanoparticles), engineered with a T12 peptide-decorated red blood cell membrane for targeted treatment of oral squamous cell carcinoma via DNA synthesis inhibition. (**II**) In vivo anti-tumor efficacy in an SCC-7 xenograft model. (**A**) Experimental treatment timeline. (**B**) Photographs of tumor-bearing mice from each group. (**C**) Images of excised tumors at the study endpoint. (**D**) Body weight changes in mice during the treatment period. (**E**) Tumor growth curves. (**F**) Final tumor weights. Representative (**G**) H&E and (**H**) TUNEL-stained tumor sections, showing necrosis and apoptosis, respectively. Bar = 200 μm, * *p* < 0.05, *** *p* < 0.001, n.s., not significant. Reproduced from [165].

Yang et al. [165] reported the in vitro drug release study assessed the pH-responsive behavior of T12- red-blood-cell membrane (RBCM)@CM nanoparticles (Figure 3). It was found that substantial methotrexate release occurred at pH 5.5 and 6.0, while only a small amount was released in a neutral medium. Specifically, after 3 h, nearly 100% of methotrexate was released in pH 5.5 phosphate-buffered solution. In pH 6.0 phosphate-buffered solution, complete release took approximately 24 h. When T12-RBCM@CM@DiD was co-cultured with SCC-7 cells for 2 h, the red fluorescence signal significantly increased, being about 4 times higher than that of the RBCM@CM@DiD group. Pretreatment of SCC-7 cells with transferrin receptor antibody significantly reduced the red signal of T12-RBCM@CM@DiD, indicating that the T12 peptide primarily enhances nanoparticle uptake through interaction with the transferrin receptor. The relative expression of transferrin receptor in SCC-7 cells was approximately 30-fold higher compared to RAW 264.7 and TR146 cells, as confirmed by flow cytometry assay. Compared to free methotrexatev, cell membrane nanoparticles significantly reduced the IC_50_ value from 31.74 ± 6.48 µg/mL to 9.32 ± 1.68 µg/mL. The T12-RBCM@CM nanoparticles further decreased the IC_50_ value to 4.68 ± 1.15 µg/mL, representing a nearly 7-fold decrease compared to free methotrexate. T12-RBCM@CM nanoparticles showed the highest activity in inducing apoptosis, with Caspase-3 expression almost twice as high as that of free methotrexate. The T12-RBCM@CM nanoparticles treatment group showed the highest percentage of dead cells (67.5% ± 3.69%). T12-RBCM@CM nanoparticles induced a substantial increase in S-phase arrest, reaching 66.02% ± 4.91%. The half-life of free DiD was less than 1 h, while CM@DiD and T12-RBCM@CM@DiD had half-lives of approximately 1.7 h and 7.5 h, respectively. Blocking the CD47 protein on T12-RBCM@CM@DiD with CD47 antibody decreased its half-life to approximately 1.6 h, similar to CM@DiD.

The tumor fluorescence signals of T12-RBCM@CM@DiD were approximately 1.8- and 11.5-fold higher than those of RBCM@CM@DiD and CM@DiD, respectively, 24 h post-injection. CM nanoparticles, RBCM@CM nanoparticles, and T12-RBCM@CM nanoparticles significantly inhibited tumor tissue growth. The T12-RBCM@CM nanoparticles treatment group exhibited the most pronounced inhibition of OSCC cell growth. TUNEL staining confirmed significant apoptosis in tumor cells treated with RBCM@CM nanoparticles and T12-RBCM@CM nanoparticles, accompanied by elevated Caspase-3 expression levels. A significant reduction in SCCA- and Ki-67-positive cells was observed in the T12-RBCM@CM nanoparticles treated group. The body weight of mice was not significantly affected by any treatment group, indicating negligible in vivo toxicity. Liver function indices and renal function parameters in the T12-RBCM@CM nanoparticles treated group remained within normal ranges, indicating no hepatotoxicity or kidney impairment. No significant changes were observed in the counts of different blood cells. In summary, the statistical results consistently demonstrate that the T12-RBCM@CM nanoplatform significantly enhanced cellular uptake, showed superior in vitro tumor inhibition, prolonged circulation time, and effectively suppressed tumor growth in vivo, all while maintaining a favorable biosafety profile [165].

Sun et al. [166] reported pH-sensitive (PEPCA@SPA/Fe(III)) nanoformulation were successfully synthesized, encapsulating sparfloxacin-ferric (Fe(III)) ion nanoparticles within an amphiphilic Poly(ethylene glycol)-poly(cinnamyl aldehyde) (PEPCA) copolymer assembly. This nanoformulation demonstrated dual functions: antibacterial activity and induction of tumor ferroptosis. The PEPCA@SPA/Fe(III) exhibited pH-sensitive drug release, with approximately 80% cumulative release of cinnamyl aldehyde in acidic endosomal/lysosomal environments (pH 5.0), significantly higher than in physiological conditions. Sparfloxacin release showed a similar pH-sensitive trend. In vitro studies showed that PEPCA@CM (coumarin-loaded) were efficiently endocytosed by 4T1 cells, with stronger green signals observed in the treated group compared to naked coumarin. In vivo analysis in orthotopic breast cancer models demonstrated that PEPCA@rhodamine B-loaded accumulated primarily in the liver, with secondary enrichment in tumor tissues, and maintained higher fluorescence intensity at tumor sites compared to free rhodamine B. PEPCA@ sparfloxacin/Fe(III) effectively induced ferroptosis in 4T1 tumor cells by disrupting the glutathione/glutathione peroxidase 4 axis. Treatment with sparfloxacin/Fe(III) and PEPCA@ sparfloxacin/Fe(III) led to the highest levels of reactive oxygen species production and lipid peroxidation, key hallmarks of ferroptosis. These nanoparticles significantly scavenged glutathione and downregulated glutathione peroxidase 4, consistently with their role in inducing ferroptosis. Ultrastructural analysis revealed profound mitochondrial abnormalities, including fragmentation and disrupted cristae morphology, in cells treated with sparfloxacin/Fe(III) or PEPCA@ sparfloxacin/Fe(III). PEPCA@sparfloxacin/Fe(III) significantly suppressed tumor growth and reduced lung metastasis in orthotopic breast cancer models, outperforming other treatment groups. The nanoformulation remodeled the intratumoral microbiota, contributing to the reduction in lung metastasis. In combination with anti-CD47 immunotherapy, PEPCA@sparfloxacin/Fe(III) activated antitumor immunity by promoting dendritic cell maturation, inducing M1 polarization of tumor-associated macrophages, down-regulating regulatory T cells, and enhancing cytotoxic T cells. This combined strategy achieved a tumor inhibition efficiency of up to 76% and prolonged survival. In summary, the study successfully developed pH-responsive PEPCA@sparfloxacin/Fe(III) that effectively eliminate intratumoral bacteria and induce ferroptosis in breast cancer cells. This nanoplatform, especially when combined with anti-CD47 immunotherapy, significantly enhances antitumor immunity and suppresses tumor progression and metastasis by remodeling the TME.

### 8.2. Redox

The TME is characterized by high levels of glutathione, which can be exploited by redox-sensitive nanoformulations. These systems are engineered to release drugs in response to the elevated glutathione levels, ensuring targeted delivery [174,177]. Redox-sensitive systems can also respond to reactive oxygen species, which are overproduced in tumor cells, providing another mechanism for site-specific drug release [176,178].

Yu et al. [167] reported a redox-responsive nanoformulation (RPSSD@IR780/docetaxel) designed for photo-chemotherapy of triple-negative breast cancer and evaluated its efficacy and safety both in vitro and in vivo. The particle size of RPSSD significantly decreased from 185.5 ± 1.86 nm to 119.8 ± 5.29 nm after incubation with 10 mM DTT, confirming its redox-sensitive nature due to disulfide bonds. This property enables drug release in the reductive TME. The cumulative drug release of docetaxel from RPSSD@IR780/docetaxel reached 76.2% within 72 h in a simulated TME (pH 5.5 + 10 mM DTT), significantly higher than in physiological conditions (pH 7.4 or pH 5.5 alone). Near-infrared light irradiation further enhanced drug release by disrupting the nanostructure. IR780 within the nanoparticles effectively generated reactive oxygen species upon near-infrared light irradiation, leading to a significant reduction in DPBF absorbance. Photothermal effects were also robust, with a temperature increase of 10.5 °C within 6 min, sufficient to kill tumor cells. RPSSD@IR780/docetaxel showed significantly higher intracellular green fluorescence in MDA-MB-231 cells compared to free IR780 or nanoparticles without RGD modification (PSSD), indicating enhanced cellular uptake due to RGD-mediated active targeting. This also led to the strongest intracellular reactive oxygen species accumulation, enhancing photodynamic therapy efficacy. RPSSD@IR780/docetaxel, especially with near-infrared light irradiation, demonstrated superior tumor cell inhibitory effects on MDA-MB-231 cells in a dose-dependent manner, leading to a significant increase in dead cells compared to other treatment groups. RPSSD@IR780/docetaxel exhibited the most robust photothermal effect in vivo, with a temperature increase of 16.2 °C upon near-infrared light irradiation, attributed to RGD-mediated tumor targeting and aggregation. The RPSSD@IR780/docetaxel + Laser group showed significantly smaller tumor volumes and weights, with approximately 92% tumor suppression. This treatment also drastically improved the survival rate, with 33.3% of mice surviving up to 50 days, compared to all mice in the saline or free IR780/DOC groups dying before day 26. H&E staining revealed significant tumor tissue damage, nuclear fragmentation, and cellular necrosis in the RPSSD@IR780/docetaxel + Laser group. Ki-67 staining showed the lowest expression in this group, indicating substantial retardation of cell proliferation. Unlike free IR780/docetaxel, which caused a significant decrease in body weight and potential myelosuppression (reduced WBC and NEU levels), RPSSD@IR780/docetaxel groups showed a slight increase in body weight and no significant alterations in liver, kidney, or cardiac function markers. Histopathological examination of major organs also revealed no abnormalities, confirming the high biosafety of the nanoparticle. In conclusion, the RPSSD@IR780/docetaxel nanoparticle demonstrated superior efficacy in inhibiting tumor growth and extending survival in triple negative breast cancer models, while also exhibiting excellent biocompatibility and biosafety. This is attributed to its redox-responsive, RGD-targeted design, which facilitates targeted drug delivery and synergistic photo-chemotherapy, overcoming limitations of traditional treatments.

### 8.3. Enzyme

Overexpressed enzymes in the TME, such as matrix metalloproteinases, can be targeted by enzyme-sensitive nanoformulations. These systems are designed to degrade or change conformation in the presence of specific enzymes, triggering drug release [174,177]. This approach allows for the selective targeting of tumor cells, minimizing the impact on healthy tissues [175,179].

Liu et al. [168] successfully developed an enzyme-responsive hydrogel system for co-delivery of cisplatin and protein arginine methyltransferase 5 (PRMT5)-short hairpin RNA. This system effectively overcame chemotherapy resistance in non-small cell lung cancer neuroendocrine differentiation (NED)-like cells by enhancing drug uptake, promoting targeted gene silencing, and inducing significant apoptosis, while showing minimal toxicity to normal cells. The methacryloylated hyaluronic acid + cisplatin@mesoporous silica nanoparticles-polyethyleneimine/PRMT5-short hairpin RNA group showed significantly stronger cytotoxicity against PC9-NED and A549-NED cells compared to cisplatin alone. This indicates that PRMT5 inhibition enhanced the sensitivity of drug-resistant cells to cisplatin. RT-qPCR results confirmed effective silencing of PRMT5 and downregulation of drug resistance-related genes MDR1 and BCRP following treatment with the hydrogel. The hydrogel exhibited reduced cytotoxicity towards normal cells, suggesting preferential uptake by lung cancer cells. Flow cytometry showed that the hydrogel formulation loaded with therapeutic agents exhibited the most pronounced pro-apoptotic effect on NED-like cells, leading to a significant increase in early and late apoptotic cells

**Figure 4:**
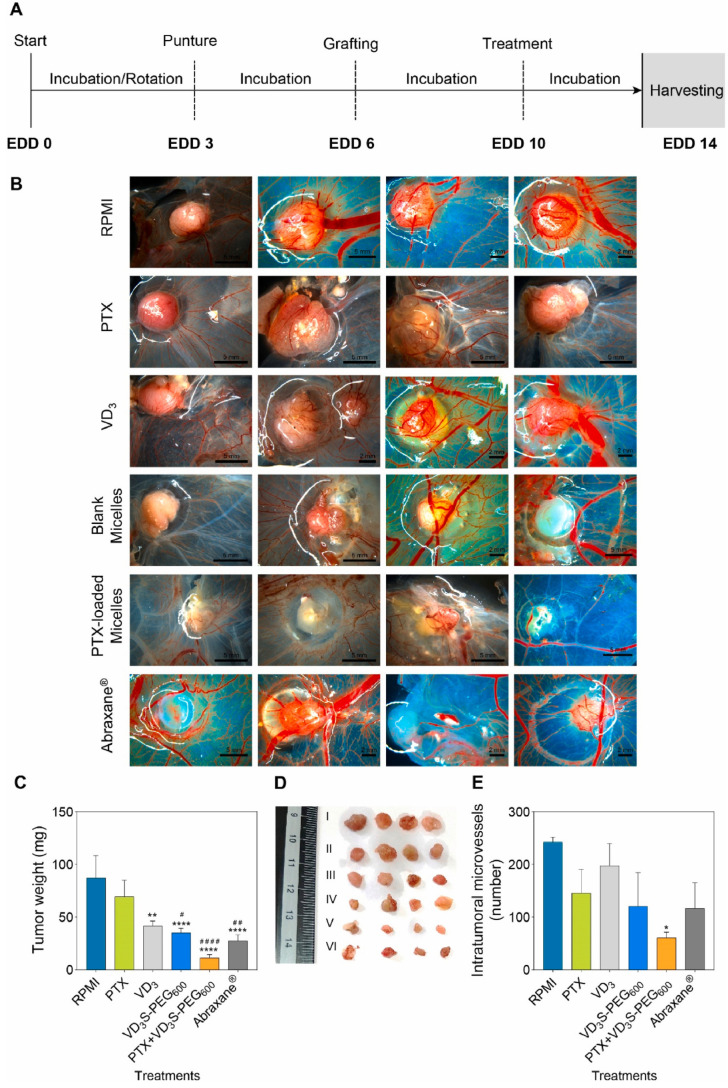
The anti-tumor and anti-angiogenic efficacy was evaluated using an in ovo chorioallantoic membrane model grafted with BxPC-3 cells (**A**). Visual and quantitative analysis demonstrated that treatment with paclitaxel + vitamin cholecalciferol succinate-poly(ethylene glycol) 600 micelles resulted in the most pronounced suppression of tumor growth, as shown by photographs of the grafts (**B**), significantly reduced final tumor weight (**C**), images of excised tumors (**D**), and a marked reduction in intratumoral vascularization (**E**), outperforming both individual agents and the clinical benchmark Abraxane^®^. Results are reported as mean ± standard deviation (*n* = 4). Results are expressed as mean ± standard deviation, * *p* < 0.05, ** *p* < 0.005, and **** *p* < 0.0001. Asterisk (*) was used to compare the antitumoral activity with the untreated group and number sign (#) was use was used to compare the antitumoral activity with free PTX. Reproduced from [169].

Peixoto et al. [169] successfully developed and characterized enzyme-responsive vitamin D-based nanoformulation for the controlled delivery of paclitaxel and demonstrated their potential for synergistic pancreatic cancer therapy (Figure 4). The study highlights the synthesis, stability, drug release characteristics, cellular uptake, cytotoxicity, and in vivo antitumor efficacy. The micelles demonstrated enzyme-triggered disassembly, with accelerated paclitaxel release (86% at pH 6.5 and 62% at pH 7.4 after 72 h) in the presence of porcine liver esterase, mimicking the pancreatic TME. This enzyme-responsive characteristic is crucial for targeted drug delivery to pancreatic ductal adenocarcinoma cells. In 2D cell culture models, paclitaxel + vitamin cholecalciferol succinate-poly(ethylene glycol) 600 micelles showed a strong cytotoxic effect against BxPC-3 cells, with lower IC_50_ values compared to free paclitaxel, free vitamin cholecalciferol, or blank micelles. A combination index of 0.86 indicated a synergistic therapeutic effect of paclitaxel and vitamin cholecalciferol. In 3D cell-laden hydrogel models, micelles significantly inhibited BxPC-3 cell growth, outperforming free paclitaxel, free vitamin cholecalciferol, and blank micelles, corroborated by live/dead assays. In an in ovo tumor model, micelles significantly inhibited tumor growth and reduced intratumoral microvessel formation compared to free paclitaxel and the approved clinical nanoformulation (Abraxane^®^). This enhanced efficacy is attributed to the synergistic anticancer effect of paclitaxel and vitamin cholecalciferol and the optimal particle size for tumor penetration.

### 8.4. Reactive Oxygen Species

Reactive oxygen species responsive nanoformulations represent a promising approach in oncology, particularly for enhancing the delivery and efficacy of anticancer drugs. These formulations leverage the elevated reactive oxygen species levels characteristic of cancer cells to trigger drug release, thereby improving therapeutic outcomes while minimizing side effects [180].

Sun et al. [170] reported the dual cross-linked chitooligosaccharide nanoparticles synthesis loaded with curcumin. The chitooligosaccharide nanoparticles exhibited reactive oxygen species responsive characteristics. Under hydrogen peroxide stimulation, the micellar structure disassembled, leading to a significant increase in particle size (from ~130 nm to ~800 nm), confirming their reactive oxygen species responsive nature. Electron microscope images further revealed structural fragmentation and irregular aggregates upon reactive oxygen species stimulation. In a 4T1 tumor-bearing mouse model, chitooligosaccharide nanoparticles significantly reduced tumor growth compared to phosphate-buffered solution, chitooligosaccharides/4-carboxyphenylboronic acid derivatives-thioketal acid, curcumin, and curcumin-glucose oxidase groups. The tumor inhibition rate reached 71.2%, outperforming previously reported curcumin nanoformulations. This efficacy was attributed to the depletion of intracellular glucose and continuous hydrogen peroxidase generation, leading to sustained curcumin release and cell death. The body weights of the mice remained stable throughout the treatment period, suggesting high biosafety and excellent biodegradability of the natural polysaccharide-based drug carriers. Serum biochemical indicators showed no significant changes, indicating no apparent toxic effects on liver or kidney function. Histopathological examination of tumor tissues and major organs confirmed no pathological damage to normal organs, while chitooligosaccharide nanoparticles treated tumor tissues showed significant cell disruption and apoptosis [170].

**Figure 5:**
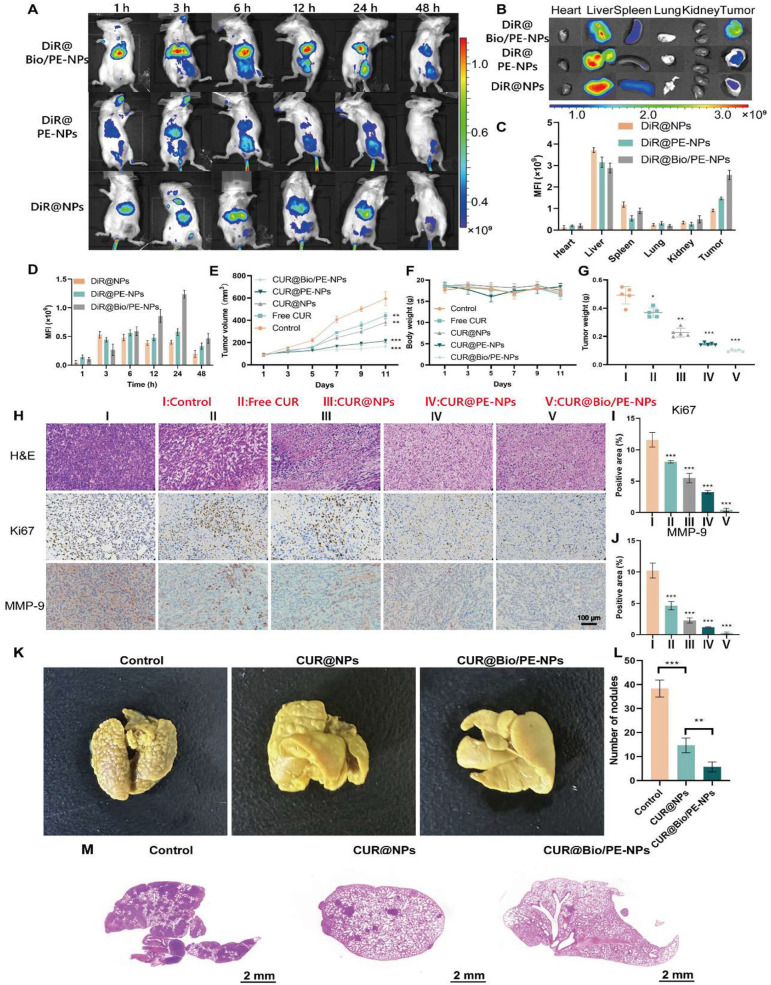
A comprehensive evaluation of biodistribution, antitumor efficacy, and anti-metastatic activity was conducted. The curcumin@biotin/PE nanoparticles showed superior tumor accumulation in 4T1-bearing mice, as confirmed by in vivo and ex vivo imaging (**A**–**D**). This targeted delivery correlated with the most significant inhibition of primary tumor growth, evidenced by reduced tumor volume/weight (**E**,**G**), and downregulation of Ki67 and MMP-9 proliferation/invasion markers (**H**–**J**), without inducing significant body weight loss (**F**). Crucially, the formulation also dramatically suppressed lung metastasis, as indicated by a reduction in visible nodules and confirmed by H&E staining (**K**–**M**). Compared to control group. *n* = 3. * *p* < 0.05, ** *p* < 0.01 and *** *p* < 0.001. Reproduced from [171].

Li et al. [171] focused on developing and evaluating a reactive oxygen species responsive nanocarrier, curcumin@biotin/PE nanoformulation, for efficient curcumin delivery to inhibit triple-negative breast cancer cell growth and migration (Figure 5). In vitro studies revealed that nanoformulation effectively induced reactive oxygen species generation within cancer cells. The targeted nanoparticles showed significantly enhanced cellular uptake, approximately 1.30-fold in MDA-MB-231 cells and 1.36-fold in 4T1 cells, compared to non-targeted nanoparticles. Nanoformulation suppressed the proliferation of triple-negative breast cancer cells, with IC_50_ values of 3.277 μg/mL for MDA-MB-231 and 5.259 μg/mL for 4T1 cells. The nanoformulation led to increased apoptosis and cell cycle arrest in cancer cells. Importantly, nanoformulation prevented cell migration and invasion, critical aspects of cancer progression. In a 4T1 tumor xenografted mice model, the nanoformulation successfully prolonged curcumin accumulation at tumor sites. It modulated the TIME, suggesting a broader therapeutic impact beyond direct cytotoxicity. The treatment prevented tumor growth and lung metastases in the animal model. The nanoformulation achieved these therapeutic benefits without significant toxicity. In summary, the in vitro and in vivo results collectively suggest that nanoformulation represent a promising strategy for the therapy of triple-negative breast cancer, demonstrating effective drug delivery, potent anti-cancer activity, and a favorable safety profile [171].

### 8.5. Hypoxia

Hypoxia-responsive nanoformulations represent a promising advancement in oncology, particularly for targeting the unique microenvironment of solid tumors characterized by low oxygen levels. These innovative systems enhance drug delivery and therapeutic efficacy by releasing their payloads specifically in hypoxic conditions, thereby improving treatment outcomes [180].

Zhu et al. [172] successfully developed and evaluated a hypoxia-responsive nanoplatform for targeted cancer treatment. The key results highlighted the efficacy of the developed nanomicelles in drug delivery, redox modulation, and enhanced therapeutic outcomes under hypoxic conditions. An amphiphilic polymer, Poly(ethylene glycol)-phthalic acid-nitroimidazole, was synthesized by linking hydrophilic Poly(ethylene glycol) to nitroimidazole acyl via an amidation reaction. This polymer was then used to create hypoxia-responsive nanoscale micelles using the organic solvent volatilization technique. The chemotherapeutic drug doxorubicin was successfully loaded into these nanoscale micelles, forming hypoxia-responsive nanoformulation. In the hypoxic and reducing TME, the hydrophobic 2-nitroimidazole component of the nanomicelles undergoes conversion into its hydrophilic counterpart, 2-aminoimidazole. This conversion is catalyzed by the overexpressed nitroreductase enzyme, which relies on the cofactor-reduced nicotinamide adenine dinucleotide phosphate. The transformation from hydrophobic to hydrophilic leads to the disruption of the micelle structure and accelerates the release of the loaded doxorubicin. The conversion of 2-nitroimidazole to 2-aminoimidazole also actively disrupts the redox balance within cancer cells. This disruption in redox balance facilitates tumor cell apoptosis. Experiments measuring changes in oxidized nicotinamide adenine dinucleotide phosphate hydrogen and glutathione content in tumor tissues of mice demonstrated that hypoxia-responsive nanoformulation could disrupt the redox balance within tumor cells under hypoxic conditions.

**Figure 6:**
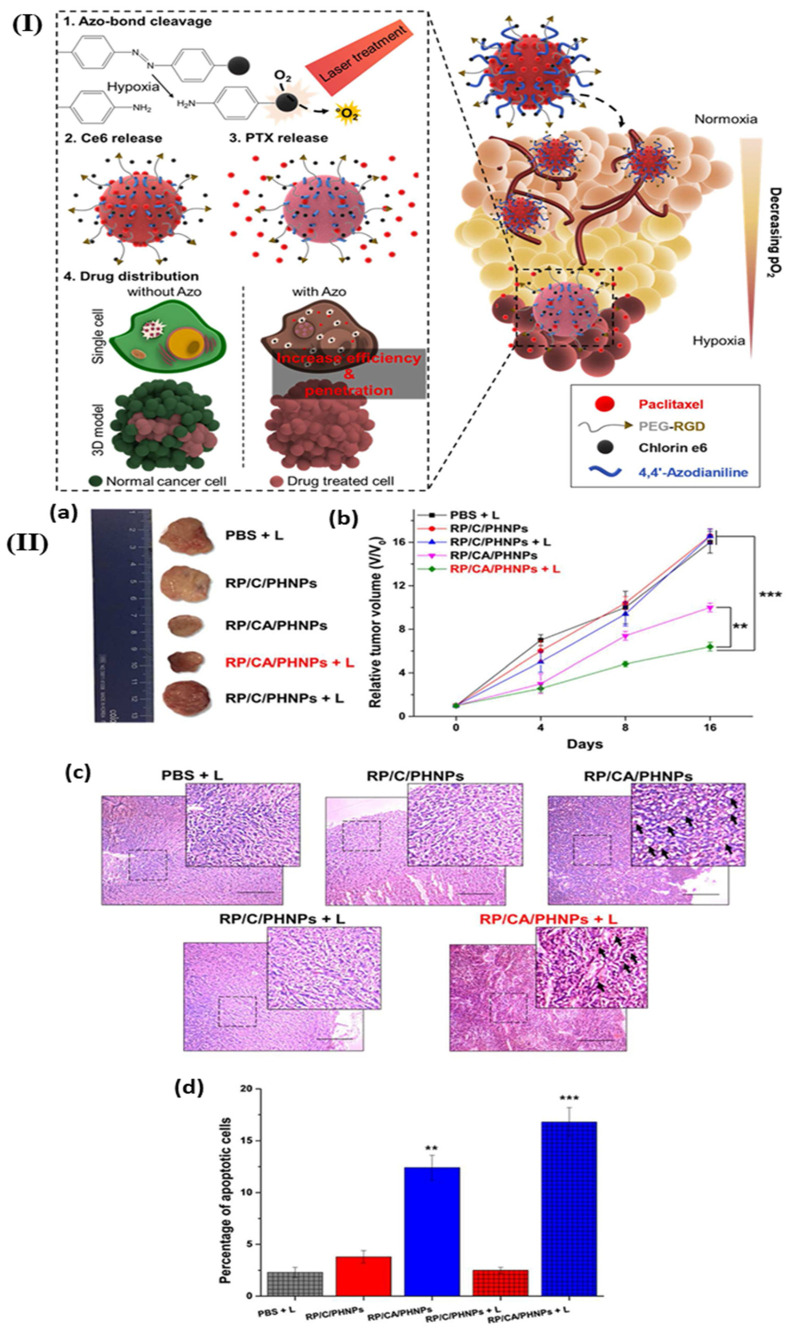
(**I**) Anticancer strategy for RP/CA/PHNPs. (**II**) The antitumor efficacy of the synthesized nanoparticles was evaluated in a HeLa xenograft mouse model. The treatment significantly inhibited tumor growth (**a**), as visually confirmed by the size of excised tumors (**b**). Histological analysis via H&E staining revealed extensive tumor cell apoptosis in the treated groups (**c**), which was quantitatively validated, showing a statistically significant increase in apoptotic cells compared to the control (**d**). ** *p* < 0.01, *** *p* < 0.001. Scale bar is 100 μm. Reproduced from [173].

Lee et al. [173] successfully developed hypoxia-responsive nanoparticles that selectively release therapeutic agents, leading to improved drug distribution and significant antitumor effects in both in vitro and in vivo models, thereby offering a promising strategy to overcome resistance in hypoxic solid tumors (Figure 6). The nanoformulation (RP/CA/PHNPs) was engineered with 4,4’-azodianiline (Azo) as a linker. Under hypoxic conditions, the azo group (-N=N-) in Azo was reductively cleaved, leading to the selective release of chlorin e6 and paclitaxel. The release of chlorin e6 triggered by azo cleavage under hypoxia resulted in a uniform distribution of chlorin e6 within HeLa cells and spheroids. This enhanced distribution of chlorin e6 significantly improved antitumor activity, even within a hypoxic environment. The nanoformulation demonstrated excellent antitumor effects in a HeLa cell xenograft mouse model. This strategy, which controls drug distribution within a hypoxic TME, represents a potentially very effective approach for treating solid tumors with a hypoxic TME. The developed nanomedicine strategy aims to improve the efficiency of both photodynamic therapy and chemotherapy by targeting the hypoxic conditions prevalent in tumors [173].

In summary, while endogenous stimuli-responsive nanoformulations offer significant advantages in targeting the TME, it is important to consider the complexity and variability of the TME across different tumor types. The heterogeneity of the TME can affect the performance of these nanoformulations, necessitating personalized approaches to cancer treatment. Additionally, the integration of endogenous and exogenous stimuli-responsive strategies may provide a more comprehensive solution to overcoming the barriers posed by the TME, further enhancing the specificity and efficacy of cancer therapies.

## 9. Externally Stimuli-Responsive Systems: Spatiotemporal Precision in Oncology

External responsive nanoformulations represent a significant advancement in oncology, particularly in enhancing the efficacy of cancer therapies while minimizing side effects. These formulations utilize various external stimuli, such as light, ultrasound, and magnetic fields, to trigger the release of therapeutic agents specifically at tumor sites (Table 12). This targeted approach not only improves drug accumulation but also addresses challenges posed by the TME. This section serves as case-study expansion of Section 3.

**Table 12:** Overview of externally activated stimuli-responsive nanoformulation systems.

Stimulus	Advantages	Limitations	Responsive Mechanisms/Materials	Example(s)	Ref.
Light	Unparalleled spatiotemporal control (on/off switching) Non-invasive Can enable combined photothermal and photodynamic therapy	Limited tissue penetration depth, especially for ultraviolet/visible light Potential phototoxicity to healthy tissues Requires specialized, precise equipment for application	Photothermal effect: Gold nanoparticles, carbon nanotubes, polydopamine Photocleavage: o-nitrobenzyl groups Photoisomerization: Azobenzene	cRGD-PTer N25/CPT nanoparticles (near-infrared triggered camptothecin release and photothermal therapy) Redox-responsive microneedles + laser for pyroptosis induction	[181,182]
Ultrasound	Deep tissue penetration Non-invasive and widely available in clinics Can enhance uptake via sonoporation (microbubble cavitation)	Complex dose–response relationship Potential for off-target tissue damage if not correctly focused Can require co-administration of cavitation agents	Mechanical or thermal disruption of nanocarriers (e.g., liposomes, micelles) Sonosensitizer activation (e.g., for sonodynamic therapy)	IMDQ-N_3_ nanoprodrugs (ultrasound-triggered TLR7/8 agonist activation) IMP@CM-PEP20 nanoplatform (ultrasound enhanced ferroptosis and immunotherapy)	[183,184]
Magnetic field	Deep tissue penetration Enables dual imaging and therapy (magnetic hyperthermia) Remote control for targeted accumulation and triggered release	Requires specialized, high-cost equipment Potential for non-specific heating in non-target tissues Guidance is limited to anatomically accessible sites	Magnetic hyperthermia: superparamagnetic iron oxide nanoparticles Magnetic field-guided targeting	Nanoferrogels (magnetic field-triggered paclitaxel release) PFH-HIONs (magneto-thermal droplet vaporization for therapy and imaging)	[185,186]
Heat (Temperature)	Can be triggered non-invasively with localized hyperthermia Sharp, tunable response with thermosensitive polymers Hyperthermia itself can have therapeutic effects and enhance drug perfusion	Requires external heating equipment (e.g., lasers, radiofrequency) Potential for damage to healthy tissue if heating is not precise and localized Heat distribution within tumors can be heterogeneous	Polymer phase transition: Poly(N-isopropylacrylamide), elastin-like polypeptides Liposome membrane permeabilization	Gold nanogels (near-infrared light induced heat for doxorubicin release)	[187]
Electric field	High spatiotemporal precision and control Can enhance drug permeation via electroporation Non-thermal and controllable, suitable for sensitive payloads	Limited to superficial or surgically accessible tumors Requires electrode placement or specialized setups Potential for tissue damage or irritation at high voltages/intensities	Electro-responsive polymers/hydrogels Electroporation of cell membranes Conductive or piezoelectric nanomaterials	Cytoplasmic accumulation of barium titanate nanoparticles in MCF-7/BT-549 cells via tumor-treating fields Electro-responsive micelles or hydrogels for on-demand release	[188,189]

### 9.1. Light

Light-responsive nanoformulations utilize specific wavelengths to trigger drug release. This method allows for precise spatial and temporal control over drug delivery. Photothermal and photodynamic therapies are common applications, where light exposure leads to localized heating or reactive oxygen species generation, respectively, causing drug release and tumor cell death [190]. These systems are particularly advantageous due to their non-invasive nature and the ability to focus light on specific tumor sites, minimizing damage to surrounding healthy tissues [191].

**Figure 7:**
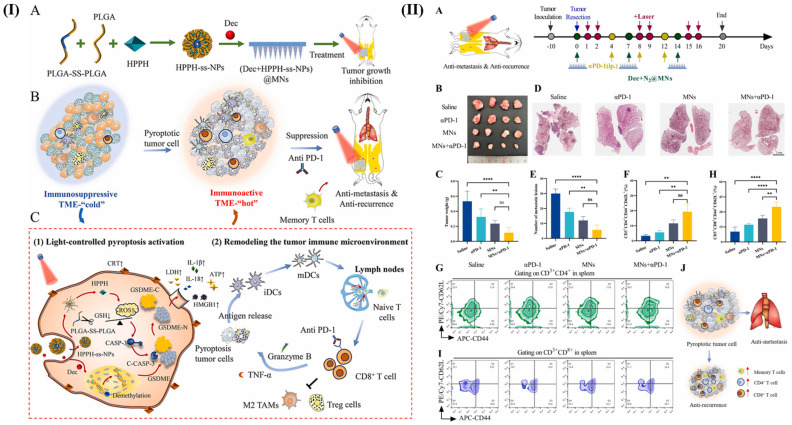
(**I**) Schematic representation of laser-triggered proptosis microneedle nanoformulation. (**II**) The efficacy of the HPPH-ss-N2/DEC-loaded microneedle patches against tumor recurrence and lung metastasis was assessed in a 4T1 breast cancer model (**A**). Treatment with the combination microneedle significantly suppressed the growth of in situ recurrent tumors (**B**,**C**) and reduced lung metastatic burden (**D**,**E**). Furthermore, flow cytometric analysis of splenocytes revealed an increased proportion of central memory T cells (CD44+CD62L+) among both CD4+ and CD8+ T cell populations (**F**–**I**), suggesting the induction of long-term immunological memory. A proposed mechanism for this combined anti-recurrence and anti-metastatic effect is illustrated in panel (**J**). ** *p* < 0.01, and **** *p* < 0.0001, respectively. Reproduced from [182].

Yu et al. [182] demonstrated light-controlled pyroptosis via redox-responsive microneedles, particularly when combined with decitabine and anti-PD-1 therapy, significantly enhanced breast cancer immunotherapy by promoting immunogenic cell death, dendritic cell maturation, and systemic anti-tumor immunity, while also preventing recurrence and metastasis (Figure 7). In orthotopic 4T1 tumor-bearing mice, the combinatorial decitabine + nitrogen + laser therapy achieved maximal tumor suppression (310.06 ± 32.30 mm^3^ final volume, 74.76% inhibition by weight), outperforming monotherapies. Histopathological analysis showed extensive tumor necrosis and reduced Ki67+ proliferative cells in treated groups. TUNEL assays indicated that combinatorial therapy induced 13.40 ± 2.25% apoptosis, paralleled by increased cleaved caspase-3 and higher GSDME-N expression. Microneedle delivery achieved preferential tumor enrichment with minimal off-target distribution, demonstrating reduced systemic toxicity and potent therapeutic outcomes. In a post-surgical breast cancer model, the therapeutic regimen combining microneedle-mediated nanoformulation with systemic PD-1 blockade achieved 77% suppression of local tumor regrowth and nearly eliminated lung metastases. This approach established long-term immunological memory, evidenced by the expansion of central effector memory T cells in splenic reservoirs, with elevated CD4+ central effector memory T cells (19.30 ± 4.95%) and CD8+ central effector memory T cells (35.03 ± 4.65%) populations.

**Figure 8:**
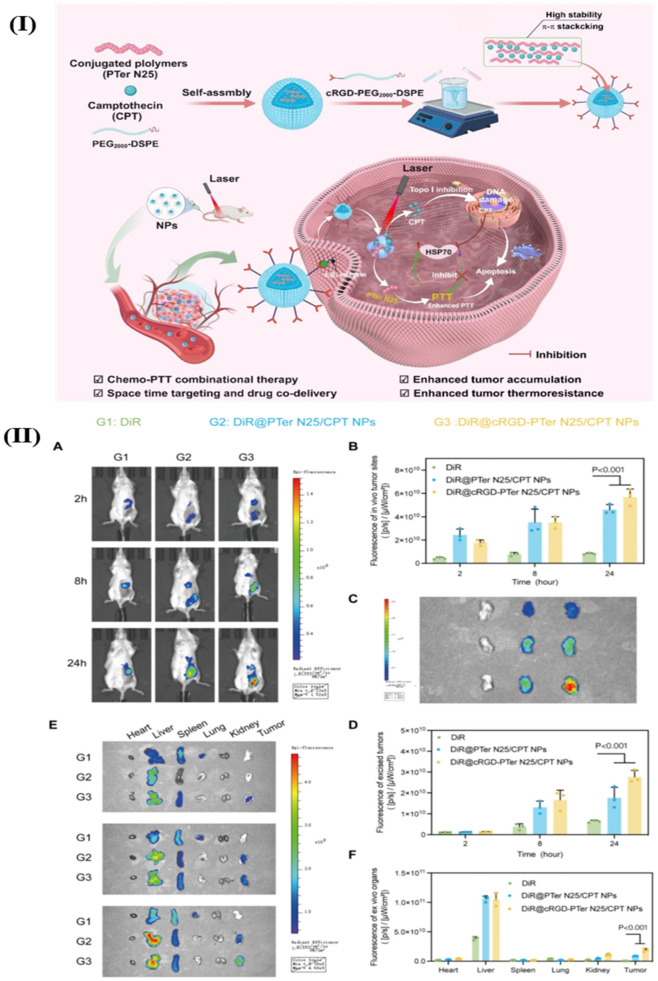
(**I**) Schematic representation of cRGD-PTer N25/camptothecin nanoformulation. (**II**) The tumor-targeting ability of cRGD-PTer N25/camptothecin nanoformulation was evaluated in vivo. IVIS imaging of mice administered with DiR-labeled nanoparticles showed significantly higher fluorescence signal at the tumor site over time for the cRGD-targeted group compared to controls (**A**,**B**). This was further confirmed by ex vivo imaging, which revealed the strongest fluorescence signal in excised tumors (**C**,**D**) and a preferential accumulation in tumors over major organs (**E**,**F**). Reproduced from [181].

Zeng et al. [181] introduced a novel nano-system, cRGD-PTer N25/camptothecin nanoformulation, designed for spatiotemporal targeted photothermal therapy and chemotherapy in breast cancer (Figure 8). The system utilizes light-responsive conjugated polymer nanoparticles for controlled camptothecin release via π-π stacking, aiming to improve combinatorial therapy outcomes. In vitro studies on 4T1 breast cancer cells showed that nanoformulation combined with laser treatment achieved the highest effectiveness in eliminating cancer cells, with an IC_50_ value of 3.35 µg/mL. This represents a 3.15-fold decrease compared to the IC_50_ without laser irradiation, highlighting the synergistic effect of photothermal therapy and chemotherapy. The combination therapy significantly increased cell apoptosis rates, reaching 64.30% ± 0.21% in the nanoformulation + laser group. The system effectively downregulates heat shock protein 70, which reduces cancer cell thermoresistance and enhances combined treatment efficacy. Camptothecin’s ability to inhibit heat shock protein 70 expression is crucial, especially under photothermal induction, where it counters the upregulation of heat shock protein 70 that typically occurs in response to thermal stress, thereby improving tumor sensitivity to photothermal therapy. In mouse models, nanoformulation group achieved a tumor growth inhibition rate of 93.6%, nearly eradicating tumor presence, while control groups showed only partial inhibition. The tumor temperature for this group increased to 60 °C under laser irradiation, surpassing other groups and demonstrating superior photothermal therapeutic effects. The study observed minimal cardiotoxicity and metastatic side effects. The nanoformulation mitigated adverse effects on major organs and reduced lung metastasis, which is typically associated with camptothecin monotherapy.

### 9.2. Ultrasound

Ultrasound-responsive nanoformulations represent a promising frontier in oncology, offering enhanced precision in drug delivery and reduced systemic toxicity. These systems leverage the mechanical and thermal effects of ultrasound to trigger the release of therapeutic agents directly at tumor sites, thereby improving the efficacy of cancer treatments. The integration of ultrasound with nanotechnology allows for the development of smart drug delivery systems that can overcome the limitations of conventional chemotherapy, such as poor bioavailability and non-specific targeting. This approach is particularly beneficial in treating resistant cancer types, such as triple-negative breast cancer and non-small cell lung cancer, by enhancing drug accumulation in tumors and minimizing off-target effects [191,192].

**Figure 9:**
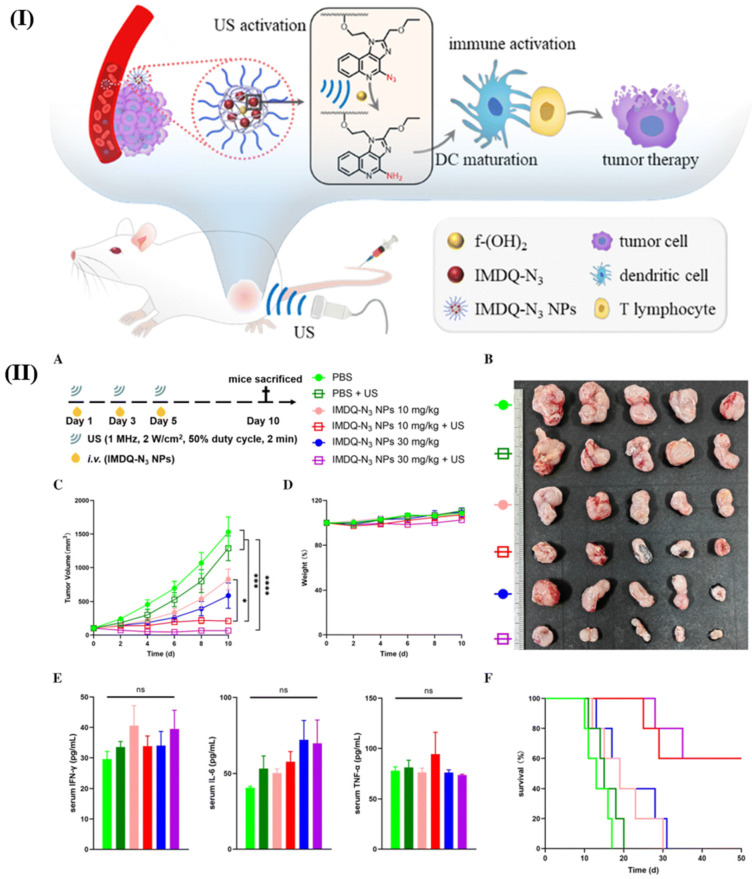
(**I**) Schematic representation of azide-modified IMDQ nano-prodrug. (**II**) The in vivo therapeutic efficacy and safety of IMDQ-N_3_ nanoparticles were systematically evaluated. Following the treatment protocol (**A**), the IMDQ-N_3_ group exhibited the most significant tumor growth inhibition, as shown by images of excised tumors (**B**) and tumor volume curves (**C**), without causing significant body weight loss (**D**). This potent anti-tumor effect was accompanied by a modulated immune response, as indicated by serum cytokine levels (**E**), and resulted in a markedly improved survival rate (**F**). P values were determined by one-way analysis of variance (ANOVA) followed by Tukey’s Honestly Significant Difference test (ns, no significant difference, * *p* < 0.05, *** *p* < 0.001 and **** *p* < 0.0001). Reproduced from [183].

Luo et al. [183] successfully developed ultrasound-responsive azide nano-prodrugs (azide modified imidazoquinolines (IMDQ-N_3_)) that enable spatiotemporally controlled activation of TLR7/8 agonists for tumor therapy (Figure 9). This nanoformulation demonstrated enhanced drug safety, superior tumor targeting, and potent antitumor efficacy through localized immune activation, leading to significant tumor suppression and improved survival rates in murine models without notable systemic toxicity. The riboflavin component was critical for efficient ultrasonic reduction, highlighting a promising platform for precision cancer immunotherapy. The nanoformulation were successfully synthesized by esterification, combining IMDQ-N_3_, riboflavin (Rf-(OH)_2_), and methoxy Poly(ethylene glycol) with Poly(L-glutamic acid). Ultrasound treatment of IMDQ-N_3_ aqueous solution led to the reduction of azide groups to amines, with a 2.0 W/cm^2^ power density being optimal. Compared to non-ultrasound conditions, 5 min of ultrasound treatment resulted in a 9.95% formation rate of active IMDQ. The presence of riboflavin in the nanoparticles was crucial for ultrasonic reduction, as IMDQ-N_3_ showed a 12.2-fold higher IMDQ formation rate (13.7%) compared to non-riboflavin-conjugated nanoparticles (1.1%) under ultrasound irradiation. In vitro studies on bone marrow-derived dendritic cells showed that ultrasound-activated IMDQ-N_3_ significantly enhanced dendritic cells maturation, with CD11c+CD80+ and CD11c+MHCII+ dendritic cells increasing to 48.3% and 37.3%, respectively. IMDQ-N_3_ significantly reduced systemic toxicity compared to free IMDQ. Mice treated with IMDQ-N_3_ did not experience weight loss, unlike those treated with free IMDQ. Serum levels of inflammatory cytokines (IL-6 and IFN-γ) were significantly lower in mice treated with IMDQ-N_3_ (maximum 1.3-fold and 3.3-fold increase, respectively) compared to free IMDQ (maximum 10.7-fold and 114.5-fold increase, respectively). In CT26 tumor-bearing mice, ultrasound-treated IMDQ-N_3_ showed significantly higher concentrations of active IMDQ in tumors. At 4 h post-injection, activated IMDQ concentrations increased by 35.2-fold, 15.3-fold, and 4.7-fold compared to various control groups. The ultrasound-activated IMDQ-N_3_ demonstrated exceptional tumor selectivity, with tumor-to-normal organ ratios reaching up to 196.3:1 (heart). Ultrasound-activated IMDQ-N_3_ significantly inhibited tumor growth, achieving tumor suppression rates of 86.2% and 95.7% at 10 mg/kg and 30 mg/kg doses, respectively, compared to the control group. The combination of IMDQ-N_3_ and ultrasound showed synergistic therapeutic effects, with Q values of 1.59 (10.0 mg/kg) and 1.41 (30.0 mg/kg). The survival rate for mice treated with ultrasound-activated IMDQ-N_3_ was 60% after 50 days, whereas all other groups succumbed within 31 days. Ultrasound-activated IMDQ-N_3_ enhanced the TIME by significantly increasing activated dendritic cells (CD11c+CD80+ and CD11c+MHCII+), T cells (CD3+CD4+ and CD3+CD8+), and M1 macrophages (CD11b+F4/80+CD80+) in the tumor. Furthermore, the treatment effectively produced lasting immunity, as evidenced by an increase in immune memory cells (CD3+CD4+CD44+ and CD3+CD8+CD44+) in the spleen.

**Figure 10:**
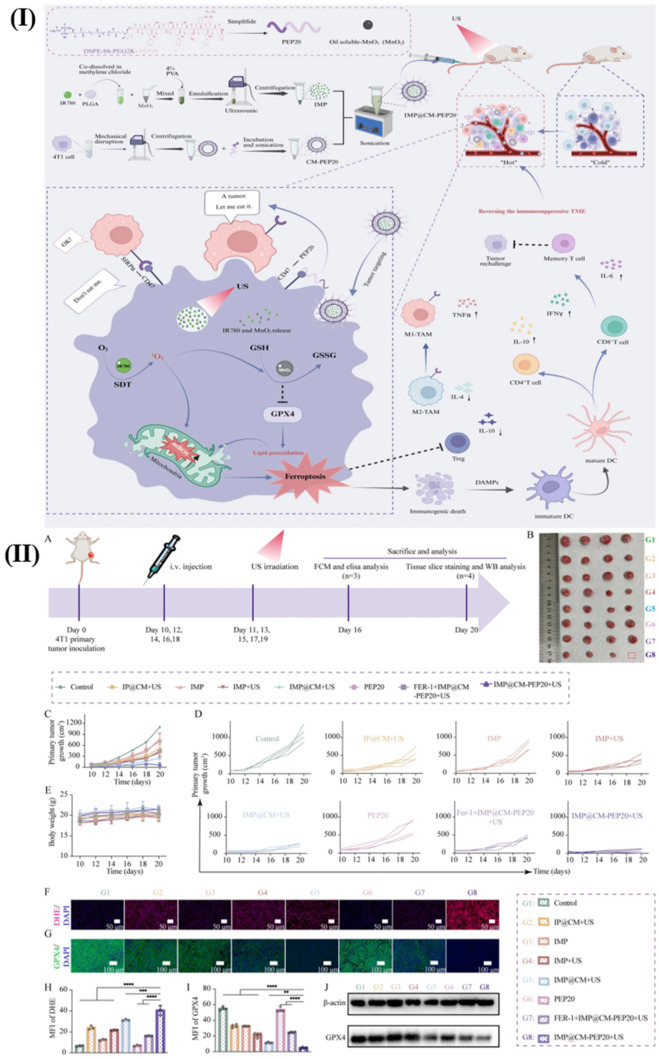
(**I**) Scheme representation of IMP@CM-PEP20 nanoparticles and their antitumor mechanisms. (**II**) The anti-tumor performance was evaluated in a 4T1 primary tumor model following the outlined treatment schedule (**A**). The IMP@CM-PEP20 + ultrasound group demonstrated the most potent efficacy, showing significant tumor regression in photographs of excised tumors (**B**) and growth curves (**C**,**D**), without systemic toxicity as indicated by stable body weight (**E**). Mechanistic analysis confirmed that this treatment induced ferroptosis in vivo, evidenced by elevated reactive oxygen species (DHE staining, (**F**,**H**)), downregulation of the key ferroptosis inhibitor glutathione peroxidase 4 (immunofluorescence, (**G**,**I**); Western blot, (**J**)). Statistical significance was performed by one-way ANOVA with a Tukey post hoc test. ** *p* < 0.01, *** *p* < 0.001, **** *p* < 0.0001. Reproduced from [184].

Liao et al. [184] developed and evaluated a biomimetic nanoplatform (IMP@CM-PEP20) designed to enhance cancer immunotherapy by targeting ferroptosis and CD47 (Figure 10). The research presented several significant findings regarding the nanoplatform’s characteristics, in vitro performance, and in vivo efficacy in a 4T1 breast tumor model.

The nanoformulation showed minimal cytotoxicity in human umbilical vein endothelial cells at 500 µg/mL, while significantly reducing the viability of 4T1 tumor cells to approximately 70% at the same concentration, attributed to higher glutathione levels in tumor cells. At 1000 µg/mL, both cell types showed significant viability reductions. The nanoformulation + ultrasound group exhibited the lowest cell viability in 4T1 cells, indicating potent tumoricidal activity. This effect was confirmed to be ferroptosis-dependent, as pre-incubation with a ferroptosis inhibitor significantly increased cell viability. Live/dead staining corroborated these findings, showing the most intense red fluorescence (dead cells) and weakest green fluorescence (live cells) in the nanoformulation + ultrasound group. The nanoformulation + ultrasound group achieved the most pronounced therapeutic effect in primary tumor models, resulting in the lowest final tumor volume and even complete tumor regression in some cases. Histopathological analysis confirmed extensive tissue damage and minimal tumor cell proliferation. In vivo, the nanoformulation + ultrasound group generated the highest reactive oxygen species levels and the lowest glutathione peroxidase 4 expression, confirming effective ferroptosis induction. The treatment significantly increased the infiltration of T-helper lymphocytes and cytotoxic T lymphocytes in tumors, while reducing regulatory T cells. It also promoted the maturation of dendritic cells in the spleen and reprogrammed tumor-associated macrophages towards an M1-like phenotype. Crucially, the nanoplatform established long-lasting immunological memory, effectively preventing tumor recurrence in a 4T1 recurrence model. Serum cytokine profiling showed elevated pro-inflammatory cytokines (interleukin-6, tumor necrosis factor-alpha) and reduced anti-inflammatory cytokines (interleukin-4), indicating a shift to a pro-inflammatory immune milieu [184].

### 9.3. Magnetic Field

Magnetic nanoparticles can be directed to tumor sites using external magnetic fields, allowing for targeted drug delivery and controlled release [190]. These systems often involve the use of superparamagnetic iron oxide nanoparticles, which can be manipulated externally to concentrate at tumor sites and release drugs in response to magnetic field changes [53,176]. Magnetic field-responsive systems offer the advantage of remote control and the potential for repeated drug release cycles [191].

Seo et al. [185] developed and characterized nanoferrogels as a theranostic platform for cancer, highlighting their synthesis, physicochemical properties, drug release capabilities, and in vivo imaging potential. Six nanoferrogels formulations were developed with magnetic nanoparticle content ranging from 0.3% to 12% (*w*/*w*). The loading capacity of magnetic nanoparticles in paclitaxel-nanoferrogels varied between 0.3% and 11.9% *w*/*w*. Exposure to a super-low frequency alternating current magnetic field (50 Hz, 50 kA/m, 30 min) significantly enhanced paclitaxel release from nanoferrogels, with release percentages of 29.3% at 4 h and 47.6% at 8 h, compared to non-exposed samples. Exposure to a pulsed alternating current magnetic field reduced the viability of LCC-6-WT cancer cells by approximately 40% at magnetic nanoparticle concentrations as low as 10 µg/mL. Fluorescently labeled paclitaxel and Poly(2-oxazoline) components of the nanoferrogels were rapidly internalized by MCF-7 cells and colocalized with lysosomes, with over 75% colocalization observed at 4 h. In vivo magnetic resonance imaging in mice bearing LCC-6-WT tumors showed that nanoferrogel/E led to signal reduction in tumors (2.7%) and lymph nodes (8.5%) 24 h post-administration, outperforming the clinically approved imaging agent ferumoxytol. This indicates prolonged retention of nanoferrogels in tumor and lymph node tissues.

**Figure 11:**
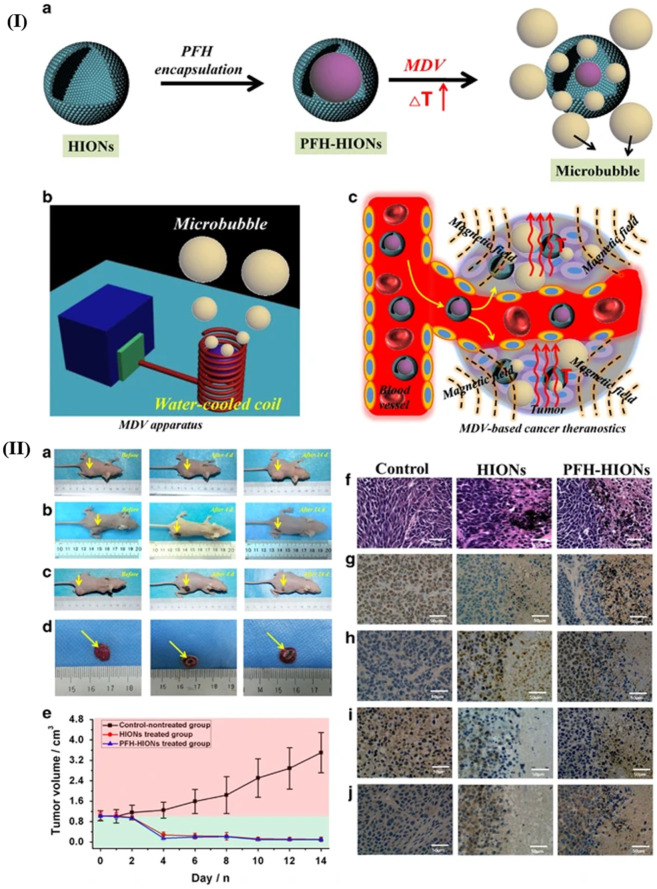
(**I**) Design of PFH-HIONs for intelligent, stimuli-responsive cancer theranostics based on magnetic droplet vaporization. (**II**) The in vivo magnetic hyperthermia efficacy of PFH-HIONs was evaluated. Photographs of tumors before and after treatment showed that the PFH-HIONs group, followed by magnetic hyperthermia, resulted in the most significant tumor ablation over 14 days (**a**–**d**). This visual outcome was corroborated by tumor growth curves, which confirmed potent and sustained suppression of tumor volume in the PFH-HIONs + magnetic field group (**e**). Histological (H&E) and immunohistochemical analysis of tumor sections provided further evidence of extensive tissue damage and reduced expression of proliferation markers in this treatment group (**f**–**j**). Reproduced from [186].

Zhou et al. [186] successfully demonstrated the feasibility and efficiency of magnetic droplet vaporization for cancer theranostics, both in vitro and in vivo, using perfluorohexane encapsulated superparamagnetic hollow iron oxide nanoparticles (PFH-HIONs) (Figure 11).

HIONs dispersed in aqueous solutions rapidly increased in temperature when exposed to an alternating current magnetic field. For instance, concentrations of 20 or 40 mg/mL caused temperature increases to 76.7 ± 6.4 °C or 147.8 ± 13.2 °C within 30 s, demonstrating high magnetic-thermal transfer efficiency. In nude mice bearing MDA-MB-231 breast cancer xenografts, intratumoral administration of PFH-HIONs followed by alternating current magnetic field exposure led to a rapid increase in tumor temperature, reaching 99.7 ± 9.8 °C and 102.3 ± 9.8 °C with HIONs and PFH-HIONs, respectively, within 3 min. The rapid temperature elevation vaporized the encapsulated perfluorohexane, leading to a significant contrast enhancement of ultrasound imaging (both B-mode and CEUS mode) in vivo. Quantitative analysis showed a 1.7-fold increase in ultrasound signal intensity, indicating high in vivo magnetic droplet vaporization efficiency. Magnetic hyperthermia assisted by PFH-HIONs effectively inhibited tumor growth, achieving an 88% inhibition rate within 4 days and 100% within 14 days, with tumors disappearing without recurrence after 2 weeks of treatment. Pathological analysis revealed clear boundaries between necrotic and non-necrotic regions in the PFH-HIONs group, with evidence of coagulated necrosis and disappearance of cell structures. Immunohistochemical staining showed fewer proliferating tumor cells, increased apoptotic cells, and reduced Bcl-2 expression in the PFH-HIONs group, confirming efficient cancer cell apoptosis and necrosis [186].

### 9.4. Heat

Heat can be used as an external stimulus to trigger drug release from thermosensitive nanoparticles. This approach exploits the temperature sensitivity of certain materials to release drugs at elevated temperatures [193]. These formulations are designed to release drugs in response to mild hyperthermia, typically in the range of 37 to 42 °C, which can be achieved through external heating methods. Hyperthermia, induced by external heat sources, can enhance drug delivery by increasing blood flow to the tumor and improving nanoparticle penetration [174]. Heat-responsive systems are particularly useful in combination with other therapies, such as radiation or chemotherapy, to enhance overall treatment efficacy [190].

2,4-Dinitrophenol loaded upper critical solution temperature nanoparticles significantly inhibited tumor growth in a mouse model, with an inhibitory efficacy of 86.5% on day 14 post-injection, and significantly increased the survival rates of mice. The average tumor volume in the 2,4-dinitrophenol loaded upper critical solution temperature nanoparticles group was around 600 mm^3^, with some tumors completely disappearing. 2,4-Dinitrophenol loaded upper critical solution temperature nanoparticles treatment led to a significant increase in CD8+ T cell infiltration in tumors (42.8% of CD8+ T cells) and spleen (30.2% of CD8+ T cells), and elevated the CD8+ T cell/Treg cell ratio by 4.3-fold in tumors. 2,4-Dinitrophenol loaded upper critical solution temperature nanoparticles significantly reduced the number of lung metastatic nodules to 5, compared to 77 in the control group. 2,4-Dinitrophenol loaded upper critical solution temperature nanoparticles did not cause significant changes in body temperature or blood pressure in mice. They also did not adversely affect liver and kidney functions, and did not cause significant damage to major organs or affect body weight. Metabolomics analysis further confirmed minimal metabolic changes in plasma, kidney, and liver compared to FDS treatment [194].

**Figure 12:**
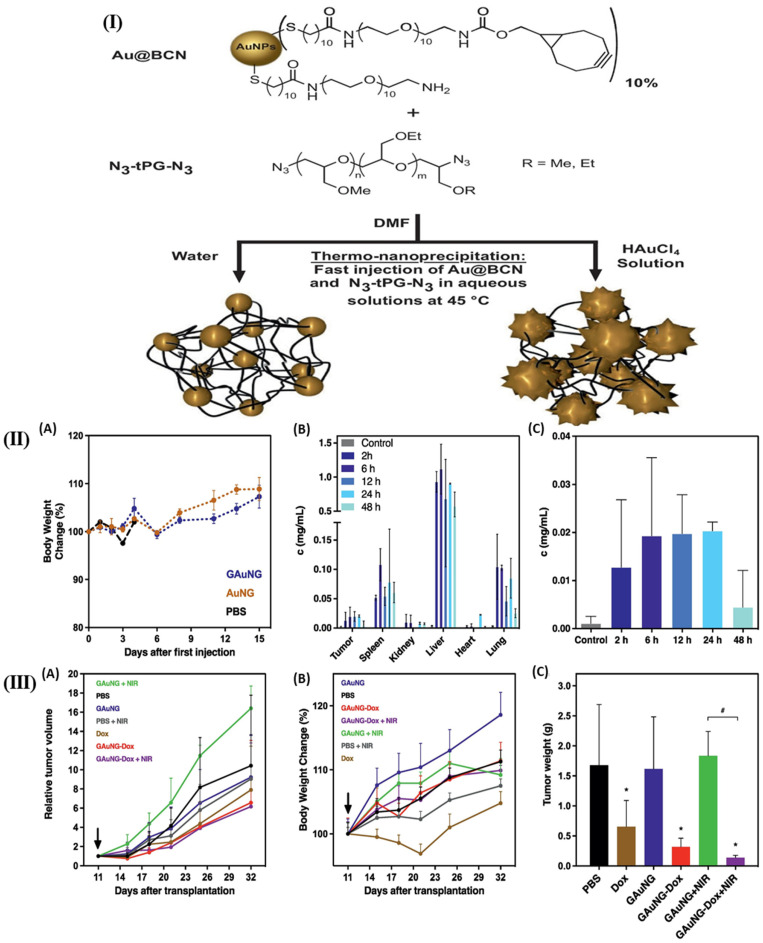
(**I**) Synthetic building blocks and schematic route for the fabrication of gold nanogels and their grown assemblies via thermo-nanoprecipitation. (**II**) (**A**) Body weight changes in mice administered the maximum tolerable dose (5 × 100 mg/kg) of GAuNGs. (**B**) Biodistribution of GAuNGs in major organs and tumors over time, quantified by inductively coupled plasma optical emission spectrometry. (**C**) Tumor accumulation kinetics of GAuNGs, as determined by inductively coupled plasma optical emission spectrometry analysis. (**III**) (**A**) Relative tumor volume over time following intravenous administration of nanogels with or without near-infrared laser irradiation, compared to phosphate-buffered solution and free doxorubicin controls. (**B**) Mean body weight changes during the treatment period; arrows indicate nanogel injections and the triangle denotes near-infrared irradiation. (**C**) Final tumor weights excised at the end of the study for each treatment group. * *p* < 0.1, significant difference compared to PBS group; # *p* < 0.1, significant difference between GAuNG and GAuNG-Dox with NIR irradiation. Reproduced from [187].

Bergueiro et al. [187] successfully developed a hybrid thermoresponsive plasmonic nanogel system that effectively integrates gold nanoparticles for near-infrared absorption and a thermoresponsive polymer for controlled drug release (Figure 12). The study successfully synthesized gold nanogels (AuNGs) by cross-linking azidated thermoresponsive Poly(glycidyl ether) with bicyclononyne-functionalized gold nanoparticles using click chemistry. A novel procedure allowed for the simultaneous growth of AuNGs into GAuNGs during synthesis, resulting in an absorbance shift from the visible to the near-infrared region. GAuNGs were effectively loaded with doxorubicin, achieving a drug loading capacity of 27.0% at an initial doxorubicin concentration of 2 mg/mL. Doxorubicin release from loaded GAuNGs was enhanced in acidic medium (pH 5) compared to neutral conditions, and further increased when the temperature was raised above the nanogel’s volume phase transition temperature. The highest cumulative release of doxorubicin (76.8%) was observed when GAuNGs-doxorubicin at pH 5.0 were irradiated with near-infrared laser for 5 min, due to the rapid transition to a hydrophobic state. In vitro studies on HeLa cells showed that both AuNGs and GAuNGs had similar cytotoxicity profiles, with viabilities decreasing at higher concentrations (10 µg/mL and above). Cells incubated with GAuNGs-doxorubicin and irradiated with near-infrared showed a significant decrease in viability (20 to 40%) compared to non-irradiated cells. At lower concentrations (25 µg/mL) where temperatures remained below 43 °C, GAuNGs-doxorubicin still significantly reduced cell viability, highlighting the importance of drug release even with moderate heating. In vivo studies in mice demonstrated that GAuNGs were highly tolerable, with no signs of acute or chronic inflammation observed even at high doses. GAuNGs-doxorubicin, especially with near-infrared irradiation, significantly inhibited tumor growth, resulting in relative tumor volume decrease by a factor of 0.6 compared to control mice. The study also noted that heat alone from unloaded GAuNGs under near-infrared irradiation could lead to increased tumor growth, emphasizing the necessity of combined chemotherapeutic drug delivery. The nanogel demonstrated excellent photothermal conversion, high drug loading, and pH/temperature-triggered release of doxorubicin. Both in vitro and in vivo experiments confirmed the system’s biocompatibility and its significant efficacy in inhibiting tumor growth through a combination of photothermal effects and chemotherapy, highlighting the critical role of design in temperature-triggered antitumoral systems.

### 9.5. Electric Field

Electric field responsive nanomaterials have emerged as a promising avenue for cancer therapy, offering targeted and controlled treatment options. These nanomaterials can be engineered to respond to external electric fields, enabling precise manipulation of cancer cells while minimizing damage to healthy tissues. The integration of nanotechnology with electric field therapies has led to innovative strategies that enhance the efficacy of cancer treatments, such as inducing cell death, improving drug delivery, and disrupting bioelectrical homeostasis in cancer cells [195].

Liu et al. [195] successfully developed an electric field-controlled nanomedicine that effectively releases drugs, significantly inhibits tumor growth in vivo, and exhibits favorable safety profiles, positioning it as a promising platform for future cancer therapies. The electric field responsive targeting drug delivery nanosystem demonstrated high electric field control. This was evidenced by promoted electric field-responsive drug release and low nonspecific leakage of doxorubicin. When breast cancer xenograft models in nude mice were treated with electric field-stimulated nanomedicine, the tumor-inhibition rate increased significantly. Specifically, the tumor-inhibition rate reached 75%, which is 2.7 times higher than treatments without electric field stimulation. The electric field responsive targeting drug delivery nanosystem was found to be biodegradable and biocompatible. It also proved to be remotely controllable by an external electric field. These results indicate that electric field responsive targeting drug delivery nanosystem represents an excellent inhibiting effect on tumors in vivo. The nanomedicine platform might become a promising candidate for electrodynamic therapy in potential clinical applications.

Xiao et al. [196] introduced a novel wireless cancer therapy approach that disturbs bioelectrical homeostasis in cancer cells using electroactive nanoparticles, leading to effective tumor treatment without invasive procedures. Ferroelectric nanoparticles, specifically K_0.5_Na_0.5_NbO_3_ and potentially BaTiO_3_, are polarized and generate an approximate −60 mV voltage when activated by ultrasound. Once these nanoparticles are endocytosed by cancer cells within tumors, remote ultrasound irradiation (for only 3 min) establishes an intracellular electric field. The generated intracellular electric field within cancer cells leads to several critical bioelectrical disturbances. Depolarization of cell membrane potentials: The intracellular electric field alters the electrical potential across the cell membrane. Decrease in mitochondrial membrane potentials: Mitochondrial function is impacted by the change in membrane potential. Overload of intracellular calcium ions: An increase in intracellular calcium levels occurs. These combined effects effectively disturb the intracellular bioelectrical balance, which is crucial for cell function. The disturbance of bioelectrical balance promotes cancer cell apoptosis (programmed cell death). This mechanism effectively inhibits the growth of different types of tumors, including bone and skin tumors. A significant finding is the specificity of the intracellular electric field: it specifically disrupts the bioelectric balance of tumor cells while having no effect on normal cells. The therapy is achieved wirelessly, without the need for electrode implantation or wire connection in vivo, and without adverse effects. This work represents the first cancer treatment paradigm that remotely interrupts intracellular bioelectrical homeostasis to cause cancer cell dysfunctions. It also provides new tools for biologists to study the role of disturbed bioelectrical homeostasis in cell fates and disease progression.

**Figure 13:**
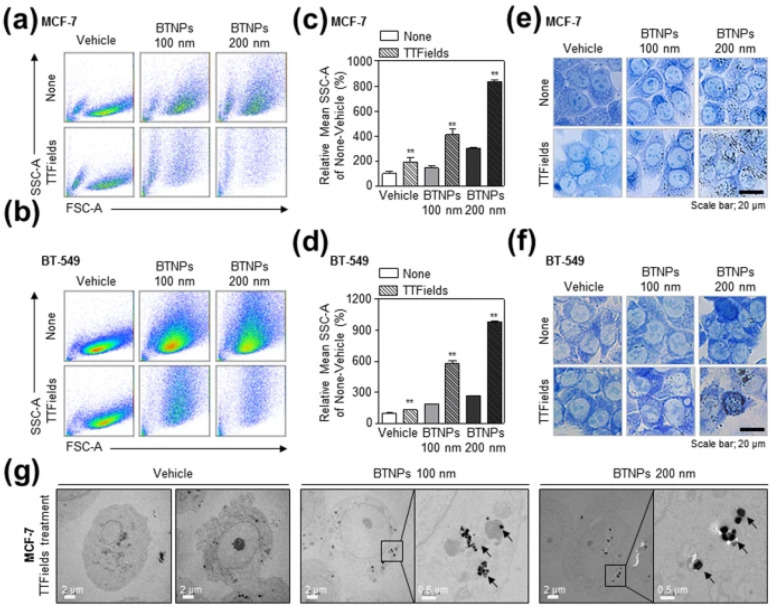
Tumor-treating fields exposure increased cytoplasmic accumulation of barium titanate nanoparticles in both MCF-7 and BT-549 breast cancer cell lines. Flow cytometry histograms (**a**,**b**) showed enhanced cellular uptake, quantified as an increase in relative granularity (**c**,**d**). Representative fluorescence images (**e**,**f**) visually confirmed cytosolic localization, which was further validated by transmission electron microscopy (**g**) in MCF-7 cells. Data represent mean ± standard deviation of five independent experiments; ** *p* < 0.01. Reproduced from [188].

Yoon et al. [188] found that barium titanate nanoparticles sensitize tumor-treating fields resistant breast cancer cells to the antitumor action of tumor-treating fields (Figure 13). Specifically, treatment with both 100 nm and 200 nm barium titanate nanoparticles enhanced the antitumor effects of tumor-treating fields in MCF-7 cells, which were identified as more resistant to tumor-treating fields compared to MDA-MB-231 and BT-549 cells. The 200 nm barium titanate nanoparticles were observed to be more potent in enhancing the antitumor activity of tumor-treating fields than the 100 nm barium titanate nanoparticles, suggesting that particle size is an important factor in this sensitizing effect. This increased potency of 200 nm barium titanate nanoparticles might be linked to their higher accumulation in the cytoplasm and a higher dielectric constant compared to 100 nm barium titanate nanoparticles. Tumor-treating fields stimulation led to the cytosolic accumulation of barium titanate nanoparticles in breast cancer cells. Flow cytometry analysis showed increased cell size and granularity in barium titanate nanoparticles/tumor-treating fields-treated MCF-7 and BT-549 cells compared to control or tumor-treating fields only treated cells. Bright-field images and transmission electron microscopy confirmed the cytosolic localization of barium titanate nanoparticles in tumor-treating fields treated MCF-7 and BT-549 cells. This accumulation is hypothesized to be due to increased membrane permeability induced by tumor-treating fields, rather than clathrin-dependent or macro-pinocytosis pathways. The combination of tumor-treating fields and barium titanate nanoparticles modulated several cancer-related pathways, with a particular focus on cell cycle-apoptosis pathways. Gene expression analysis revealed significant modulation of pathways such as cell cycle-apoptosis, Wnt, transcriptional migration, TGF-β, driver gene, Notch, JAK-STAT, and Ras signaling in barium titanate nanoparticles/tumor-treating fields -treated MCF-7 cells. Specifically, barium titanate nanoparticles combined with tumor-treating fields inhibited cell cycle progression, evidenced by a significant decrease in levels of CDK6 and transcription factor E2F1, and an increase in p21 levels. FACS analysis further indicated that the combination induced cell-cycle arrest at the G1 phase and slightly increased apoptosis.

Gangrade et al. [189] demonstrated the successful development and efficacy of a photo-electro active nanocomposite silk hydrogel for spatiotemporally controlled drug release and solid tumor growth suppression in vivo. The developed drug delivery system was a photoelectro active nanocomposite silk-based hydrogel designed for on-demand drug release in vivo. It incorporated a functionally modified single-walled carbon nanotube loaded with doxorubicin embedded within a cross-linker-free silk hydrogel. The nanocomposite hydrogel exhibited responsiveness to an electrical field and showed a near-infrared laser-induced hyperthermal effect. Remote application of these stimuli, either in tandem or independently, led to increased thermal and electrical conductivity of the hydrogel, effectively triggering intermittent on-demand drug release. In a proof-of-concept in vivo tumor regression study, the nanocomposite hydrogel was administered minimally invasively at the periphery of the tumor, covering most of it. Over a 21-day study period, significant tumor regression was observed upon regular stimulation of the nanocomposite hydrogel with simultaneous or individual external application of an electric field and near-infrared laser. Analysis of tumor cell death markers revealed the induction of apoptosis in tumor cells, leading to tumor shrinkage. Heart ultrasound and histology examinations indicated no cardiotoxicity associated with the localized doxorubicin treatment. The authors highlight that this is the first report demonstrating the simultaneous application of an electric field and near-infrared laser in vivo for localized tumor therapy. The results suggest that this strategy holds high clinical translational potential.

In summary, while externally stimuli-responsive systems hold great promise for achieving spatiotemporal precision in oncology, challenges such as limited penetration of certain stimuli and the need for biocompatibility must be addressed. Continued research and development in this field, including the exploration of novel materials and technologies, are essential to fully realize the potential of these systems in precision cancer therapy. The integration of these systems with emerging technologies could further enhance their effectiveness and applicability in clinical settings.

## 10. Bio-Orthogonal Chemistry in Tumors: Click-to-Release Strategies at the Target Site

The integration of bio-orthogonal chemistry into nano-oncology has emerged as a promising avenue for enhancing targeted drug delivery and therapeutic efficacy. Central to this approach are click-to-release strategies, which leverage bio-orthogonal reactions to activate or release therapeutic agents precisely at the tumor or target site, minimizing systemic toxicity and improving treatment outcomes. Recent advancements have demonstrated the versatility of bio-orthogonal reactions in constructing multifunctional nanoformulations for cancer therapy. For instance, Zuo et al. [197] reported a ‘one-stitch’ bio-orthogonal prodrug activation system based on cross-linked click chemistry, which enabled controlled drug release in vivo. This approach exemplified how stimuli-responsive nanosystems can be engineered to respond to specific bio-orthogonal triggers, facilitating on-demand activation of therapeutics within the TME. Such strategies are particularly advantageous in overcoming the limitations of conventional chemotherapy, such as off-target effects and systemic toxicity.

Building on this foundation, the development of active targeting platforms utilizing bio-orthogonal chemistry has gained significant traction. Niu et al. [198] described a bio-orthogonal copper-free click chemistry strategy for the active targeting of cancer cells using DBCO-lipo nanoparticles. This method allows for the precise attachment of targeting ligands or therapeutic agents to nanoparticle surfaces, enhancing specificity and cellular uptake. Similarly, bio-orthogonal reactions such as strain-promoted azide–alkyne cycloaddition have been employed to modify cell surfaces and facilitate targeted delivery [199]. These reactions are characterized by their biocompatibility and rapid kinetics, making them suitable for in vivo applications.

The application of bio-orthogonal chemistry extends beyond drug delivery to include imaging and immunotherapy. For example, bio-orthogonal click chemistry has been utilized for PD-L1-targeted imaging, enabling visualization of tumor immune checkpoints with high specificity [200]. Such imaging strategies are crucial for patient stratification and monitoring therapeutic responses. Moreover, bio-orthogonal reactions have been integrated into immunotherapeutic approaches, such as designing nanoadaptors for enhanced recognition of natural killer cells to tumor cells [201]. These nanoadaptors employ bio-orthogonal receptors to facilitate immune cell targeting, thereby potentiating anti-tumor immune responses.

In the context of nano-oncology, the concept of click-to-release has been further exemplified by the development of stimuli-responsive nanovesicles. Lu et al. [202] reported reactive oxygen species responsive multifunctional fusion extracellular nanovesicles carrying rapamycin, which address early ischemia–reperfusion injury and inflammatory rejection in heart transplantation. Although their study focused on transplantation, the underlying principle of stimuli-responsive activation via bio-orthogonal reactions is directly applicable to cancer therapy, where the tumor microenvironment provides unique triggers such as reactive oxygen species or enzymatic activity.

The clinical translation of bio-orthogonal click-to-release strategies is an ongoing challenge, but recent reviews highlight promising approaches relying on click-to-release reactions for on-target drug activation [203]. These methods aim to improve therapeutic index and reduce off-target effects by ensuring that active agents are released only within the tumor or target tissue. The development of such systems involves designing bio-orthogonal reactions that are rapid, selective, and biocompatible, with minimal interference from biological molecules. Furthermore, the combination of nanoformulations with bio-orthogonal chemistry has been shown to enhance recognition and targeting of immune cells. For instance, a designable nanoadaptor was developed to improve natural killer cell recognition of tumor cells, employing bio-orthogonal receptors for enhanced specificity [201]. This approach underscores the potential of bio-orthogonal chemistry not only for direct drug delivery but also for modulating immune responses within the TME.

In summary, the integration of bio-orthogonal click-to-release strategies into nano-oncology offers a multifaceted platform for targeted therapy, imaging, and immunomodulation. The ability to engineer stimuli-responsive nanocarriers that activate therapeutics precisely at the tumor site addresses critical challenges in cancer treatment, such as systemic toxicity and drug resistance. As research progresses, the development of biocompatible, rapid, and highly selective bio-orthogonal reactions will be pivotal for translating these innovative strategies into clinical practice, ultimately advancing personalized and effective cancer therapies.

## 11. Multi-Stimuli Responsive Nanoformulations

The development of multi-stimuli responsive nanoformulations has emerged as a promising frontier in oncology, offering innovative strategies for targeted diagnosis and therapy. These nanocarriers are engineered to respond to multiple tumor-specific stimuli, thereby enhancing therapeutic efficacy while minimizing systemic toxicity. The existing research underscores the significance of designing multifunctional nanoparticles that can adapt to the complex TME and deliver therapeutic agents precisely where needed.

One of the foundational aspects of these nanoformulations is their ability to respond to various stimuli such as pH, temperature, enzymes, light or magnetic fields. For instance, a study on chitosan-functionalized magnetite/Poly(ε-caprolactone) nanoparticles highlights their potential in improving cancer diagnosis and treatment through multifunctionality [204]. These nanoparticles can be tailored to respond to specific stimuli, enabling controlled drug release and enhanced imaging capabilities. Similarly, the design of degradable silica nanoparticles that are responsive to multiple stimuli demonstrates their utility in advanced, cancer-specific chemotherapy [205]. Such systems are engineered to degrade selectively within the tumor microenvironment, thereby reducing off-target effects and improving therapeutic outcomes.

Advances in drug delivery systems have further propelled the field, with multiple stimuli-responsive nanoparticles being developed for precise oncotherapy. A comprehensive review discussed how these systems can be engineered to respond sequentially or simultaneously to different stimuli, facilitating controlled and targeted drug release [206]. For example, nanoparticles that respond to both pH and temperature can exploit the acidic and hyperthermic conditions of tumors, ensuring that drugs are released preferentially at the tumor site. This dual-responsiveness enhances the specificity and efficacy of chemotherapeutic agents, reducing adverse effects on healthy tissues [206]. Moreover, the integration of stimuli-responsive features into nanoparticle design extends beyond simple drug delivery. Sequentially responsive therapeutic peptide assembling nanoparticles have been developed for dual-targeted cancer immunotherapy, illustrating the potential for combining multiple therapeutic modalities within a single nanoplatform [206]. Such systems can be programmed to respond to distinct stimuli at different stages of treatment, optimizing therapeutic payload delivery and immune activation. Similarly, metal-phenolic network-based nanoparticles have been constructed to respond to multiple stimuli, enabling stepwise activation and drug release, which is particularly advantageous in overcoming tumor heterogeneity and resistance [207].

The rational design of multifunctional nanocarriers is crucial for achieving precise targeting and controlled release. For example, stimuli-responsive nanocarriers that respond to TME cues such as acidity, hypoxia, or enzymatic activity have been extensively studied. A review emphasized how these nanocarriers can be tailored to exploit the unique biochemical signatures of tumors, thereby enhancing selectivity and reducing systemic toxicity [208]. Such targeted approaches are vital for improving the therapeutic index of anticancer agents. External stimuli such as light, heat, and magnetic fields have also been employed to trigger drug release from nanocarriers. Thermo-, photo-, and multi-stimuli responsive block copolymer systems demonstrate significant responsiveness to external triggers, enabling on-demand drug release with spatial and temporal control [209]. These systems are particularly promising for localized therapy, where external stimuli can be precisely applied to tumor sites, minimizing damage to surrounding healthy tissue.

Metal–organic frameworks (MOFs) have gained attention as versatile platforms for multi-stimuli-responsive drug delivery. Copper-MOFs functionalized with keratin exemplify how MOFs can be engineered to respond to multiple stimuli, facilitating controlled drug release and improving therapeutic efficacy [210]. The high porosity and tunability of MOFs make them suitable for encapsulating various therapeutic agents and responding to tumor-specific cues. Furthermore, the aggregation behavior of nanoparticles driven by multiple stimuli has been explored to enhance targeting and retention within tumors. Multi-stimuli-responsive aggregation of nanoparticles can be triggered by environmental cues, leading to improved accumulation and retention at tumor sites [211]. This approach leverages the tumor microenvironment to activate nanoparticle assembly, thereby enhancing local drug concentration and therapeutic effect.

In summary, the recent literature underscored the multifaceted potential of multi-stimuli responsive nanoformulations in oncology. These systems offer sophisticated control over drug delivery, enabling targeted, efficient, and minimally invasive cancer therapy. The integration of various stimuli-responsive mechanisms—ranging from biochemical cues to external triggers—provides a versatile toolkit for overcoming current limitations in cancer treatment. Continued research and development in this domain are poised to translate these innovative nanotechnologies into clinical applications, ultimately improving patient outcomes.

## 12. Controlled Release Kinetics for Optimized Cancer Pharmacodynamics

The development of nanoformulations for cancer therapy hinges critically on the ability to precisely control drug release kinetics to maximize therapeutic efficacy while minimizing adverse effects. Recent advances in nanotechnology have provided innovative strategies to tailor drug release profiles, thereby optimizing pharmacodynamics in cancer treatment. This section overviews findings from literature studies, emphasizing the importance of controlled release mechanisms in nanoformulations designed for cancer therapy.

One prominent approach involves the use of nanocarrier systems that incorporate specific surface modifications or structural features to modulate drug release. DuRoss et al. [212] demonstrated the potential of inflammation-targeting nanoscale MOFs coated with fucoidan to enhance chemoradiotherapy in colorectal cancer. Their findings suggest that nanocarriers can be engineered to respond to tumor-specific stimuli, such as radiation-induced P-selectin expression, thereby enabling targeted and controlled drug release. This inflammation-responsive mechanism exemplifies how nanocarrier design can be leveraged to achieve site-specific release, which is crucial for improving therapeutic outcomes. Similarly, the use of bioconjugation techniques to influence release kinetics has been explored. Escareño et al. [213] employed microfluidic-assisted conjugation to attach antibodies like Trastuzumab to drug-loaded polymeric nanoparticles. Their study revealed that such conjugation not only enhances targeting but also affects drug release profiles, as analyzed through DDSolver. The smaller, less dispersed nanoparticle–antibody conjugates exhibited differential release kinetics, underscoring the importance of surface engineering in controlling drug release behavior.

Mathematical modeling plays a vital role in understanding and predicting release kinetics. Heredia et al. [214] conducted a statistical comparison of various kinetic models applied to Poly(lactic-co-glycolic acid)-based nanocarriers. Their analysis identified the most suitable models for describing drug release profiles, which is essential for rational nanoformulation design. Such modeling efforts facilitate the optimization of release parameters to achieve sustained and controlled drug delivery, directly impacting pharmacodynamic profiles. The integration of nanocarriers with hydrogels offers another promising avenue for controlled release. Kass and Nguyen [215] reviewed nanocarrier–hydrogel composite systems, emphasizing how the incorporation of nanocarriers into hydrogels allows for high-resolution control over drug dosing and release kinetics. The use of 3D printing techniques to fabricate hydrogels with precise geometries further enhances the ability to tailor release profiles, which is particularly advantageous for localized cancer therapy.

Lipid-based nanocarriers have also been optimized for sustained release. Iqubal et al. [216] formulated lipid–nanosystem gels containing 5-fluorouracil and resveratrol, demonstrating significantly prolonged and slow drug release following non-Fickian Higuchi kinetics. This controlled release profile ensures a sustained therapeutic concentration at the target site, reducing dosing frequency and improving patient compliance. The concept of stimuli-responsive release mechanisms is gaining traction. Xie et al. [217] investigated heat stress-induced ferroptosis, revealing that moderate heat could modulate lipid metabolic pathways and antioxidant expression in cancer cells. Such insights suggest that external stimuli like heat can be harnessed to trigger or accelerate drug release selectively within the TME, providing an additional layer of control over pharmacodynamics.

Furthermore, the design of layered or barrier-modified nanocarriers enables fine-tuning of release kinetics. Howard et al. [218] described self-assembled layer-by-layer films coated with clay layers to modulate the release of growth factors like bone morphogenetic protein 2 from implants. This approach demonstrates how diffusional barriers can be engineered to achieve sustained release over desired timeframes, which is critical for regenerative and therapeutic applications in oncology. The use of liposomes as controlled release vehicles has also been extensively studied. Prathyusha et al. [219] optimized curcumin-loaded liposomes via QbD approaches, achieving smaller vesicle sizes and improved entrapment efficiencies. The release from these liposomes followed non-Fickian Higuchi kinetics, indicating a combination of diffusion and erosion mechanisms that can be tailored for desired release profiles.

Targeted delivery systems further exemplify the importance of controlled release. Chaudhari et al. [220] developed adenosine-conjugated Poly(lactic-co-glycolic acid) nanoparticles for triple-negative breast cancer, achieving specific targeting and sustained drug release. Similarly, Kousar et al. [221] designed hyaluronic acid-coated chitosan nanoparticles for cisplatin delivery, demonstrating how surface modifications influence release behavior and targeting efficiency. Mathematical and computational models are instrumental in optimizing release kinetics. Sumetpipat et al. [222] employed heuristic algorithms to model drug encapsulation within carbon nanotubes, revealing patterns that could be exploited to control release. Such models provide valuable insights into the physical mechanisms governing drug release, enabling the rational design of nanoformulations with predictable pharmacodynamic profiles. In addition to carrier design, the incorporation of nanocarriers into advanced delivery systems like bioresponsive hydrogels or layered films offers further control. Kass and Nguyen [215] and Howard et al. [218] highlighted how these composite systems can be engineered to provide sustained, localized, and stimuli-responsive release, which is particularly advantageous in cancer therapy where spatial and temporal control over drug delivery is paramount.

In summary, the research underscored the critical role of controlled release kinetics in enhancing the pharmacodynamics of nanoformulations for cancer treatment. Strategies such as surface conjugation, barrier engineering, stimuli-responsiveness, and mathematical modeling collectively contribute to the development of sophisticated delivery systems capable of providing sustained, targeted, and tunable drug release. These advancements are poised to significantly improve therapeutic efficacy, reduce systemic toxicity, and pave the way for personalized nanomedicine approaches in oncology.

## 13. Immuno-Nanoformulations: Reprogramming the Tumor Immune Microenvironment

The landscape of cancer immunotherapy has been significantly transformed by the advent of immuno-nanoformulations designed to reprogram the TIME. These innovative strategies aim to convert immunologically ‘cold’ tumors, which are characterized by low immune cell infiltration and immunosuppressive features, into ‘hot’ tumors that are more amenable to immune attack and responsive to therapies such as immune checkpoint blockade. The collective insights from recent studies underscore the multifaceted approaches employed to modulate various components of the TIME, including tumor-associated macrophages, T lymphocytes, extracellular vesicles, and metabolic pathways, through nanotechnology-based interventions.

Beyond modulating the local tumor immune landscape, nanoformulations serve as a foundational platform for developing systemic cancer vaccines [223]. Nanoparticle-based vaccines are designed to prime and activate the host immune system de novo against tumor antigens. Nanocarriers—including lipid nanoparticles, polymeric particles, and inorganic structures—address key limitations of traditional vaccines by protecting antigen and adjuvant cargo, enhancing their co-delivery to antigen-presenting cells in lymphoid tissues, and promoting robust antigen cross-presentation to stimulate cytotoxic T lymphocytes [224]. This platform enables versatile strategies, from delivering defined neoantigens to encapsulating whole tumor lysates for personalized vaccination. Furthermore, the physicochemical properties of nanoparticles (e.g., size, surface charge) and their functionalization with targeting ligands or immunostimulatory molecules can be engineered to control the magnitude and type of the ensuing immune response [225]. As such, nanovaccines represent a powerful prophylactic and therapeutic tool that synergizes with other immuno-nanoformulations by establishing a systemic, tumor-specific immune response capable of targeting both primary and metastatic sites [226].

One prominent approach involves utilizing nanoformulations to induce immunogenic cell death, thereby transforming the tumor microenvironment from cold to hot. Jin et al. [227] demonstrated that corn-like gold/silver nanorods could mediate near-infrared photothermal and photodynamic therapy, which effectively induces immunogenic cell death in tumor cells under 1064 nm light irradiation. This process enhances the presentation of tumor antigens and promotes immune cell infiltration, thereby potentiating the efficacy of immune checkpoint antibodies. Such nanostructures serve as a foundation for combining physical tumor ablation with immunomodulation, highlighting the potential of nanotechnology to reprogram the TIME.

Targeting tumor-associated macrophages has emerged as a central strategy given their prevalence and immunosuppressive role within the TIME. Molgora et al. [228] reviewed current strategies aimed at reprogramming tumor-associated macrophages from an M2-like, immunosuppressive phenotype to an M1-like, immunostimulatory state. Similarly, Wang et al. [229] engineered biomimetic nano-red blood cells to target endogenous tumor-associated macrophages, reprogramming them to support anti-tumor immune responses and enhance chemo-immunotherapy. These approaches underscore the importance of modulating tumor-associated macrophages polarization to shift the TIME from immunosuppressive to immunostimulatory, thereby facilitating T cell infiltration and activation.

The role of T lymphocytes, particularly CD8+ cytotoxic T cells, is critical in mediating tumor eradication. Xie et al. [230] emphasized the importance of understanding the interplay between CD8+ T cells and stromal components within the TIME, which influences their antitumor responses. Strategies to enhance T cell infiltration and function are further exemplified by Wang et al. [231], who employed oncolytic adenoviruses armed with chemokines like CXCL11 to improve CAR-T cell infiltration and reprogram the immunosuppressive TIME in glioblastoma. Additionally, Gao et al. [232] developed injectable hydrogels loaded with lipid nanoparticles carrying immune regulatory factors, which promote M1 macrophage polarization and subsequent T cell activation, illustrating the integration of nanotechnology with immune modulation.

Extracellular vesicles have garnered attention as natural nanocarriers capable of mediating intercellular communication within the TIME. Han et al. [233] reported that ginseng-derived nanoparticles could reprogram macrophages from an M2 to an M1 phenotype, thereby enhancing the efficacy of PD-1 monoclonal antibodies and inducing systemic anti-tumor immunity. Similarly, Bao et al. [234] provided a comprehensive overview of tumor-derived extracellular vesicles, highlighting their role in modulating immune responses and facilitating tumor progression. These vesicles can be engineered or harnessed to deliver immunomodulatory agents directly to tumor sites, offering a promising avenue for reprogramming the TIME.

Metabolic reprogramming within the TIME is another critical aspect addressed by nanotechnology. Yu et al. [235] developed ATP-exhausted nanocomplexes to interfere with tumor energy metabolism, thereby modulating the immune microenvironment to favor anti-tumor responses. Zhang et al. [236] introduced nano-PROTACs capable of photo-metabolic cancer immunotherapy, which utilize protease inhibitors to alter intratumoral metabolic processes and reprogram the TIME. These approaches demonstrate how nanomaterials can target tumor metabolism to alleviate immunosuppression and enhance immune cell activity.

The modulation of immune cell phenotypes and functions through nanotechnology extends to other immune components as well. Wu et al. [237] utilized arginine-loaded iron oxide nanoparticles to induce gaseous immunotherapy, which can influence the TIME’s immunomodulatory landscape. Khosravi et al. [238] emphasized the importance of turning cold tumors hot by employing various immunologic modulators, including nanomaterials, to enhance immune infiltration and response. Furthermore, the use of engineered B cells [239] and dendritic cell reprogramming [240] exemplified how cellular therapies combined with nanotechnology can potentiate antigen presentation and long-term immune memory.

Overall, the integration of nanotechnology into immunotherapy offers a versatile platform for reprogramming the TIME. These nanoformulations can deliver a broad spectrum of immunomodulatory agents—ranging from photothermal agents, cytokines, metabolic inhibitors, to extracellular vesicles—targeting multiple components of the TIME simultaneously. The ability to precisely manipulate immune cell phenotypes, enhance antigen presentation, and modulate metabolic pathways underscores the potential of immuno-nanoformulations to overcome the limitations of current therapies, particularly in cold tumors resistant to conventional immunotherapy [241,242,243].

In summary, recent advances in immuno-nanoformulations have demonstrated promising strategies to reprogram the TIME, thereby improving the efficacy of immunotherapies. By targeting tumor-associated macrophages, T cells, extracellular vesicles, and metabolic pathways, these nano-based approaches aim to convert immunologically ‘cold’ tumors into ‘hot’ ones, facilitating robust anti-tumor immune responses. Continued research in this domain is poised to yield more effective, personalized cancer treatments that leverage the synergy between nanotechnology and immunology.

## 14. Combination Nanomedicine: Rational Co-Delivery of Synergistic Agents

The advancement of nanomedicine has significantly transformed the landscape of cancer therapy, particularly through the development of combination nanomedicine strategies aimed at co-delivering multiple therapeutic agents to achieve synergistic effects. This section highlights recent research studies focused on the rational design and application of nanoformulations for the co-delivery of synergistic agents in oncology, emphasizing targeted delivery, controlled release, and microenvironment-responsive systems.

Gold nanoparticles have garnered attention as versatile platforms for targeted co-delivery due to their biosafety profile and ease of functionalization. Zhang et al. [1] demonstrated the use of gold nanoparticles equipped with affibody–DNA hybrid strands for the targeted co-delivery of 5-fluorodeoxyuridine and doxorubicin, achieving synergistic chemotherapy in HER2-overexpressing breast cancer. Confocal laser scanning microscopy showed significantly higher uptake of doxorubicin@affi-F/gold nanoparticles by HER2-overexpressing BT474 cells compared to HER2-low-expressing MCF-7 cells, demonstrating the effectiveness of affibody-mediated targeting. Affi-F/gold nanoparticles (containing FUdR) exhibited higher cytotoxicity in BT474 cells (IC_50_ = 6.95 μM) than in MCF-7 cells (IC_50_ = 29.98 μM), indicating a selective inhibitory effect on HER2-overexpressing cancer cells. Doxorubicin@affi-F/gold nanoparticles demonstrated a higher inhibitory effect on BT474 cells compared to a simple mixture of free doxorubicin and FUdR. Combination Index analysis confirmed strong synergism for doxorubicin@affi-F/gold nanoparticles in both low and high-inhibition areas for BT474 cells, suggesting a precise drug ratio within the nanoparticles. Doxorubicin@affi-F/gold nanoparticles induced a significantly higher apoptosis rate (44.2%) in BT474 cells compared to the simple mixture of doxorubicin and FUdR (32.1%) and individual drugs. This indicates that the nanocarrier promotes more cells to enter the apoptosis pathway, contributing to its enhanced antitumor activity. This approach highlights the potential of inorganic nanomaterials not only as imaging agents but also as effective drug carriers capable of precise targeting and combination therapy.

Similarly, the integration of nanotechnology with personalized medicine is exemplified by Ianevski et al. [244], who employed machine learning combined with single-cell RNA sequencing to predict patient-specific drug combinations. Their approach enabled the identification of synergistic drug pairs capable of targeting distinct leukemic subpopulations, emphasizing the importance of tailored combination strategies that consider tumor heterogeneity and disease stage [244]. Phototheranostics has also been explored as a means to enhance combination therapy efficacy. Hu et al. [245] designed an all-organic nanoplatform for magnetic resonance/near-infrared-II imaging-guided cancer phototheranostics, facilitating precise tumor localization and treatment. Yasothamani et al. [246] developed hyaluronan-polyaniline-imiquimod nanoparticles that serve as photothermal agents, inducing immunogenic cell death and potentiating immunotherapy in triple-negative breast cancer. These studies underscored the utility of nanoformulations in integrating diagnostic and therapeutic functions, enabling spatiotemporal control over treatment modalities.

Lan et al. [247] developed a biomimetic immunostimulatory nanomodulator, 4T1 membrane@polydopamine-gambogic acid (Tm@PDA-GA), designed to enhance anticancer immunity through a combination of photothermal therapy and vessel normalization. Polydopamine acts as a drug carrier within the nanomodulator. It is capable of inducing photothermal therapy when exposed to near-infrared irradiation. Furthermore, polydopamine contributes to immunogenic cell death, which is crucial for activating dendritic cells. Gambogic acid is released on-demand in the acidic TME. Gambogic acid inhibits the expression of heat shock proteins, leading to synergistic chemo-photothermal anti-tumor activity and increasing the immunogenic cell death of 4T1 cells. Crucially, gambogic acid normalizes tumor vessels by inhibiting hypoxia-inducible factor 1-alpha and vascular endothelial growth factor, which enhances immune cell infiltration and alleviates hypoxia stress within the tumor. Tm@PDA-GA successfully induced immunogenic cell death. It activated dendritic cells and stimulated cytotoxic T cells. The nanomodulator also suppressed regulatory T cells, which are known to inhibit anti-tumor immune responses. The combination of Tm@PDA-GA with anti-PD-L1 therapy further augmented the tumor immune response. This combined approach effectively suppressed tumor growth and lung metastasis. The study concludes that the biomaterial-mediated photothermal therapy combined with vessel normalization, as demonstrated by Tm@PDA-GA, represents a promising strategy for effective immunotherapy of triple-negative breast cancer.

Targeted and stimuli-responsive nanocarriers have been extensively investigated to improve drug accumulation at tumor sites and minimize off-target effects. Sun et al. [248] constructed pH-sensitive nanoparticles for co-delivering doxorubicin and tanshinones in prostate cancer, demonstrating enhanced therapeutic performance. Zhang et al. [249] developed tumor-activated, photothermal-augmented nanocarriers for breast cancer, combining chemotherapy with photothermal therapy and endogenous microenvironment responsiveness to achieve synergistic tumor eradication. Hollow mesoporous organosilica nanoparticles (HMON) showed high biosecurity, revealing no obvious cytotoxicity against 4T1 cancer cells even at 200 µg/mL after 48 h. Disulfiram anticancer bioactivity was significantly enhanced by copper: cell viability dropped from over 90% (without Cu(II)) to 62.3% (0.2 µmol/mL disulfiram), 23.8% (0.5 µmol/mL disulfiram), and 8.2% (1.0 and 2.0 µmol/mL disulfiram) with Cu(II). Copper sulfide nanoparticles alone demonstrated low cytotoxicity, with no significant effect on 4T1 cancer cells at 25 µg/mL in 24 h. The combination of HCu (HMONs-ss-copper sulfide) or disulfiram@HCu with photothermal therapy significantly lowered cell viability to 15.4% or 8.3%, respectively, compared to 85.2% for photothermal therapy alone. The apoptosis rate of 4T1 cancer cells treated with disulfiram or disulfiram@HMONs was 13.4% or 17.3%, respectively, comparable to the control group (11.3%). After photothermal therapy alone, apoptosis slightly increased to 26.4%. The apoptosis rate dramatically increased with combination treatments: 36.4% for disulfiram + copper chloride, 74.7% for disulfiram@HCu, 59.7% for HCu + photothermal therapy, and 86.2% for disulfiram@HCu + photothermal therapy, with the latter showing maximum therapeutic efficacy. HCu administered to mice (0, 5, 10, and 20 mg/kg) showed no evident major organ damage or significant side effects within one month, as validated by body weight, hematology, serum biochemistry, and histological analyses. HCu rapidly accumulated at the tumor site within 6 h, reaching a maximum tumor uptake of 5.8 ± 0.1%. After intravenous administration of HCu or disulfiram@HCu, the tumor-site temperature in 4T1 tumor-bearing mice increased from 35 °C to 61.8 °C or 62.8 °C, respectively, after 10 min of 808 nm laser exposure (1.5 W/cm^2^). The HCu + photothermal therapy group showed tumor regression of 83.3%. The disulfiram@HCu group exhibited tumor regression of 69.9%. With 808 nm laser irradiation, disulfiram@HCu presented effective growth suppression, and the suppression rate was calculated to be 94.9%. This indicated nearly complete tumor destruction without recurrence.

Similarly, Rahmani et al. [250] designed pH-sensitive micelles co-delivering doxorubicin and conferone, inducing apoptosis in breast cancer cells, illustrating the importance of microenvironment-triggered drug release. Blank micelles showed no significant changes in the cell cycle pattern, confirming their low toxicity. However, doxorubicin–conferone-loaded micelles, free doxorubicin–conferone, doxorubicin-loaded micelles, and free doxorubicin all caused G2/M phase arrest in MDA-MB-231 cells. Conferone-loaded micelles induced S phase arrest. Both S and G2/M arrests are indicators of strong inhibition of DNA duplication and can lead to apoptosis. The co-drug loaded β-cyclodextrin grafted poly maleate-co-poly (lactide-co-glycolide) micelles resulted in a higher G2/M arrest (86.4%) at a lower IC_50_ dosage compared to other combinations. Annexin-V/PI double staining flow cytometry revealed that co-drug-loaded micelles induced the highest level of apoptosis (98.7%) with minimal necrosis (1.33%) in MDA-MB-231 cells, surpassing single-drug-loaded micelles or free drugs. This high level of apoptosis is linked to the increased solubility and intracellular uptake of conferone when delivered via micelles. Real-time polymerase chain reaction results indicated that co-drug-loaded micelles caused concurrent upregulation of Bax and downregulation of Bcl-2, followed by upregulation of caspase-9, caspase-3, and caspase-7. This suggests that the nanoformulations, particularly the co-drug-loaded micelles, induce apoptosis via the caspase-dependent and intrinsic mitochondrial pathway. Western blotting confirmed these findings at the protein level. Co-drug-loaded micelles significantly increased the expression of Bax (1.75-fold), cleaved-caspase-9 (5.41-fold), cleaved-caspase-3 (14-fold), cleaved-caspase-7 (22.55-fold), p27 (3.2-fold), and p53 (2.87-fold), while decreasing Bcl-2 (0.67-fold), pro-caspase-9 (0.37-fold), pro-caspase-3 (0.56-fold), and pro-caspase-7 (0.34-fold) compared to the control group. This pattern of protein expression further supports the activation of the intrinsic mitochondrial apoptosis pathway, leading to cell cycle disturbance and apoptotic death.

Photodynamic therapy combined with chemotherapy has been facilitated by nanocarriers that enable controlled co-delivery of photosensitizers and chemotherapeutic agents. Wu et al. [251] reported a pH-sensitive supramolecular nanosystem co-loading chlorin e6 and triptolide, which exhibited acid-responsive drug release and enhanced cellular internalization, leading to synergistic chemo-photodynamic effects. Similarly, Li et al. [252] developed dual-sensitive nanoparticles co-delivering chlorin e6 and doxorubicin, further exemplifying the integration of stimuli-responsive systems for enhanced therapeutic synergy. The TME has been exploited to design smart nanocarriers that respond to specific stimuli such as redox conditions, pH, or enzymes. Tarannum et al. [253] engineered nanoparticles for pancreatic cancer that provided redox-responsive, tumor-activated delivery of gemcitabine and cisplatin, with differential release mechanisms to overcome drug resistance. Zhou et al. [254] introduced a near-infrared-II nanotheranostic platform that utilized TME activation for colon cancer, combining photothermal therapy with chemotherapy to achieve complete tumor eradication with minimal side effects.

Metal-based nanocarriers have been employed to facilitate multi-targeted therapy. Yang et al. [255] developed gallium(III) agents exploiting TME properties and lactoferrin for co-delivering metal-based drugs and proteins, aiming for multi-targeted therapeutic effects. Ismail et al. [256] utilized targeted liposomes for delivering artesunate and temozolomide to resistant glioblastoma, demonstrating the potential of lipid-based nanocarriers in overcoming drug resistance through combination therapy. Emerging supramolecular systems have shown promise in co-delivering multiple agents with controlled release profiles. Duan et al. [257] designed size-controllable, multi-responsive DNA nanogels based on host–guest recognition for dual-drug delivery, offering a versatile platform for combination therapy. Wei et al. [258] fabricated luminescent nanocucurbits capable of co-delivering hydrophilic and hydrophobic chemotherapeutics, emphasizing the importance of nanostructure design in achieving effective co-delivery.

In summary, these studies collectively underscored the importance of rational nanocarrier design in achieving effective combination therapy in oncology. Strategies such as stimuli-responsiveness, targeting ligands, and multifunctionality enable precise delivery and controlled release of synergistic agents, thereby enhancing therapeutic efficacy while minimizing adverse effects. The integration of diagnostic and therapeutic functionalities, as seen in phototheranostic platforms, further exemplifies the trend toward personalized and precision nanomedicine. As the field advances, the continued development of smart, TME responsive nanocarriers and multi-agent co-delivery systems holds promise for overcoming current therapeutic limitations and improving clinical outcomes in cancer treatment.

## 15. Nanoformulations for Overcoming Multidrug Resistance

Nanotechnology-based nanoformulations have emerged as a promising strategy to overcome multidrug resistance in oncology, addressing one of the most significant hurdles in effective cancer treatment (Table 13). The complexity of multidrug resistance involves various mechanisms, including efflux transporter activity, TME barriers, and cellular signaling pathways. The existing literature underscored the potential of nanoformulations to circumvent these resistance pathways, enhance drug delivery, and improve therapeutic outcomes [259,260].

A central mechanism contributing to multidrug resistance is the overexpression of ATP-binding cassette (ABC) transporters such as P-glycoprotein and ABCG2, which actively efflux chemotherapeutic agents out of cancer cells, reducing drug accumulation and efficacy [261]. Goebel et al. [261] highlighted the importance of understanding the structural basis of these transporters to design effective inhibitors, but nanoformulations offer an alternative approach by bypassing transporter-mediated efflux altogether. For instance, nanocarriers can facilitate intracellular delivery of drugs, shielding them from recognition and expulsion by efflux pumps [261]. The controversy surrounding the role of ABC transporters in multidrug resistance is revisited by Sharma et al. [262], who emphasized that while these transporters are significant, multidrug resistance is multifaceted, involving additional cellular and microenvironmental factors. Nanoformulations can address this complexity by enabling targeted delivery and controlled release, thereby reducing off-target effects and overcoming resistance mechanisms that are not solely transporter-dependent.

Nanomedicine applications in pancreatic cancer exemplify how nanocarriers can improve drug penetration and overcome therapy resistance. Greene et al. [265] discussed how nanomedicines can exploit the unique features of pancreatic tumors, such as dense stroma and hypoxia, to enhance drug delivery and efficacy. Similarly, in breast cancer, Cabaud et al. [268] demonstrated that nanoparticle-based antibody–drug conjugates can be engineered to overcome resistance mechanisms, such as long-term treatment-induced resistance, by facilitating sustained drug release and targeted delivery. Natural compounds like hesperetin have also been integrated into nanoformulations to enhance their anticancer potential and overcome multidrug resistance. Sohel et al. [269] reviewed how hesperetin-loaded nanocarriers can sensitize cancer cells to chemotherapeutics by targeting multiple pathways, including cell cycle regulation and apoptosis, thus overcoming resistance patterns. This approach exemplified how nanoformulations can improve the bioavailability and therapeutic index of phytochemicals, which often face bioavailability challenges in their free form.

Precision medicine approaches further complement nanotechnology strategies by targeting molecular alterations associated with multidrug resistance. Musyuni et al. [263] emphasized that understanding the molecular basis of resistance allows for the design of nanocarriers that deliver drugs specifically to resistant tumor cells, thereby enhancing efficacy and reducing toxicity. Such targeted delivery systems are crucial in tackling the heterogeneity of resistance mechanisms across different tumor types. Innovative nanoplatforms are also being developed to modulate the TME, which plays a pivotal role in multidrug resistance. Xiang et al. [264] overviewed nitric oxide-based nanomedicines that can modulate the TME, overcoming physical and biochemical barriers that hinder drug penetration. These nanomedicines can induce tumor vasodilation and normalize abnormal vasculature, facilitating better drug delivery and overcoming microenvironment-induced resistance.

Biomimetics and theranostics represent another frontier in nanoformulation research. Anitha et al. [270] reviewed how biomimetic nanoparticles can enhance targeting and immune evasion, thereby improving drug accumulation in resistant tumors. Similarly, Dhanabalan and Shanmugam [271] highlighted the success of nanotherapeutic products in preclinical and clinical settings, especially in lung and breast cancers, where they demonstrate improved efficacy over conventional therapies. The integration of phytochemicals into nanoformulations is also gaining traction. Qutub et al. [266] discussed epigallocatechin gallate delivery systems that improve bioavailability and therapeutic efficacy, which is critical for overcoming resistance. Likewise, natural compounds like niclosamide [272] and isorhamnetin [273] are being formulated into nanoparticles to target multiple resistance pathways, including oxidative stress and signaling cascades.

Mesoporous silica nanoparticles have been extensively studied for their ability to deliver drugs directly to the nucleus, bypassing efflux mechanisms and enhancing combination therapies [274]. Similarly, nanoemulsions improve targeted delivery and bioavailability, especially in breast cancer, where conventional treatments face significant resistance issues [275]. Co-encapsulation strategies further enhance synergistic effects and help bypass multidrug resistance by delivering multiple agents simultaneously [276]. Emerging nanoplatforms such as MOFs, metal–phenolic networks, and 2D metal nanosheets are designed for precision delivery and bioresponsive release, addressing hypoxia and immunosuppression that contribute to resistance [277]. Copper-based nanomedicines targeting cuproptosis—a form of cell death—are also being explored to reverse resistance mechanisms, adding another layer of therapeutic intervention [267]. Finally, biodegradable polymers like Poly(ortho esters) are being developed for controlled drug release, which can modulate drug exposure and reduce resistance development [278]. The development of such versatile nanocarriers underscores the importance of tailoring nanomedicine strategies to specific resistance mechanisms, thereby enhancing the overall efficacy of cancer therapies.

In summary, the current literature demonstrated that nanoformulations hold significant promise in overcoming multidrug resistance in oncology. By facilitating targeted delivery, bypassing efflux pumps, modulating the tumor microenvironment, and enabling combination therapies, nanomedicines can address the multifaceted nature of multidrug resistance. Continued research into nanocarrier design, functionalization, and integration with molecular targeting strategies is essential to translate these promising approaches into clinical success.

## 16. Translational and Clinical Perspectives

The successful translation of smart nanoformulations from promising preclinical results to clinical reality hinges on addressing critical translational and clinical perspectives. This section highlights the importance of long-term safety (biopersistence), the emerging paradigm of integrated diagnosis and therapy (theranostics), and the critical role of advanced, human-relevant disease models. By confronting these practical challenges, the path toward clinically viable and effective nano-oncological treatments can be paved.

### 16.1. Biopersistence and Safety: Designing Nanoformulations for Clinical Translation

The advancement of nanotechnology has significantly impacted oncology, offering innovative strategies for targeted drug delivery, imaging, and therapeutic interventions. Central to these developments is the challenge of ensuring biopersistence and safety of nanoformulations to facilitate their successful clinical translation. The literature underscored the importance of understanding nanoparticle behavior within biological systems, optimizing design parameters, and addressing safety concerns to harness the full potential of nanomedicine in cancer therapy.

One of the pioneering efforts in this domain is the development of polymeric nanoparticles for anti-cancer treatment, exemplified by Lv et al. [279], who designed micellar D-melittin nanoparticles. Their work marks the first application of micellar D-melittin in cancer therapy, highlighting the potential of peptide-based nanoformulations. The study emphasized that the design of such nanoparticles must consider biocompatibility and stability to prevent premature degradation and ensure effective delivery. The authors suggested that the structural integrity of these micelles is crucial for maintaining therapeutic efficacy while minimizing toxicity, which is a recurring theme in nanomedicine safety considerations [279]. The broader implications of nanoformulation safety is addressed by Ghosh et al. [280], who advocated for a deeper understanding of peptide-nanoparticle interactions. They highlighted that elucidating the interplay between peptides and nanocarriers is vital for predicting biopersistence and minimizing adverse effects. The authors called for the development of advanced spectroscopic and computational methodologies to characterize these interactions comprehensively. Such approaches can inform the design of nanoformulations with optimized stability and reduced toxicity, thereby enhancing their safety profile for clinical applications [280].

Nanomedicine’s transformative potential is further discussed in the context of clinical translation, with emphasis on safety and biopersistence. Kurul et al. [281] reviewed how nanomaterials are revolutionizing drug delivery, underscoring that their clinical success hinges on understanding their biodistribution, clearance, and long-term tissue retention. They noted that biopersistence, particularly of inorganic nanoparticles like silver, can pose safety risks due to accumulation in tissues, as demonstrated in studies involving Sprague-Dawley rats [282]. This highlighted the necessity of designing nanoparticles with controlled biopersistence, either through biodegradable materials or surface modifications that facilitate clearance.

The classification and therapeutic application of nanoparticles are explored by Pandey et al. [283], who focused on targeting inflammatory pathways such as the NLRP3 inflammasome with curcumin-loaded nanocarriers. They underscored that the safety and efficacy of such formulations depend on their ability to deliver bioactive compounds effectively while avoiding unintended immune activation or toxicity. The authors further emphasized that understanding the mechanisms governing nanoparticle stability and degradation is essential for ensuring safe clinical translation [283]. Addressing the challenges associated with metal-based nanoparticles, Zhang et al. [282] discussed toxicity concerns, particularly related to surface characteristics that influence biopersistence and immune responses. Metal nanoparticles, while promising for their therapeutic properties, can accumulate in tissues, leading to potential toxicity. They advocated for surface engineering strategies to mitigate these risks, such as coating or functionalization, which can modulate biopersistence and improve biocompatibility.

Advanced formulation strategies are highlighted by the work of Herdiana [284], which presents targeted nanoparticles like BIND-014. Despite demonstrating favorable safety profiles, these formulations face translational hurdles, often related to stability and biopersistence. The author suggested that optimizing nanoparticle design to balance stability with biodegradability is critical for safe and effective clinical use [284]. The design and functionalization of nanobiomaterials are crucial for controlling biopersistence. As detailed by the Ramburrun et al. [285], nanobiomaterials such as liposomes, micelles, and dendrimers are tailored to improve drug delivery while minimizing long-term tissue retention. Surface modifications, such as PEGylation, are commonly employed to reduce recognition by the immune system and facilitate clearance, thereby addressing safety concerns related to accumulation [285]. Photothermal nanomaterials, discussed by Cui et al. [286], exemplify another class of nanomaterials with clinical potential. Their ability to convert light into heat for tumor ablation has been tested in clinical pilot trials, demonstrating safety and efficacy. However, the long-term biopersistence of these materials remains a concern, emphasizing the need for designing biodegradable photothermal agents that can be safely eliminated post-treatment. Finally, the integration of nanotechnology in immune modulation and vaccine development is reviewed by Azharuddin et al. [287], where nanovaccines are employed for cancer immunotherapy. The safety of such formulations depends on their ability to induce immune responses without causing adverse inflammation or persistent tissue retention. Preclinical validation is essential before clinical translation, with a focus on understanding nanoparticle biodistribution and clearance mechanisms.

In summary, the literature underscored that the safe clinical translation of nanoformulations in oncology hinges on meticulous design strategies that address biopersistence and toxicity. Advances in characterization techniques, surface engineering, and biodegradable materials are pivotal in developing nanomedicines that are both effective and safe. As the field progresses, a comprehensive understanding of nanoparticle behavior within biological systems will be instrumental in overcoming translational challenges and realizing the full therapeutic potential of nanotechnology in cancer care.

### 16.2. Theranostic Nanoformulations: Integrating Diagnosis and Treatment

The field of theranostic nanoformulations has emerged as a transformative approach in oncology, aiming to seamlessly integrate diagnostic and therapeutic functionalities within a single nanoplatform. This section overviews precise tumor detection, targeted therapy, and real-time monitoring, thereby enhancing treatment efficacy and minimizing adverse effects. The recent literature underscored various nanomaterials and strategies that have been developed to realize this vision, highlighting their potential and current challenges.

One prominent class of nanomaterials explored for theranostics is stimuli-responsive hydrogels, which are engineered to respond to specific TME cues. Zhang et al. [288] reviewed the utility of these hydrogels in cervical cancer, where they are tailored to exploit the unique features of the TME for targeted therapy and diagnosis. These hydrogels can undergo structural changes in response to stimuli such as pH, enzymes, or temperature, enabling controlled drug release and enhanced imaging contrast precisely at tumor sites. Such stimuli-responsive systems exemplify the precision that nanotechnology can bring to cancer theranostics [288]. In addition to hydrogels, mesoporous silica nanoparticles, particularly MCM-41-based structures, have gained attention for their versatile theranostic capabilities. Pires et al. [289] reported on the integration of magnetic resonance imaging and chemotherapy agents within MCM-41 nanoparticles, demonstrating their potential for simultaneous tumor detection and treatment. These silica-based nanocarriers can be functionalized with various imaging agents and therapeutic molecules, offering a platform for multimodal imaging and targeted drug delivery. Their high surface area and tunable pore size make them suitable for loading diverse therapeutic agents, thus enabling personalized treatment regimens [289].

Polymer-based nanoparticles also constitute a significant segment of theranostic systems. As discussed by Shanahan et al. [290], these nanoformulations can be engineered to carry both diagnostic markers and therapeutic agents, facilitating combined imaging and treatment. Their biocompatibility and ease of functionalization make them attractive candidates for clinical translation. Moreover, polymeric systems can be designed to respond to specific stimuli, such as pH or enzymatic activity, further enhancing their targeting precision [290]. Carbon-based nanomaterials, including multifunctional carbon nanoparticles, have demonstrated promising theranostic applications. Hosseini et al. [291] highlighted their ability to integrate treatment and diagnosis through their intrinsic optical properties and capacity for functionalization. These nanoparticles can serve as contrast agents for imaging modalities like fluorescence or photoacoustic imaging while simultaneously delivering therapeutic payloads. Their multifunctionality allows for real-time monitoring of therapeutic responses, which is crucial for personalized oncology [291].

Nanomedicine’s role in transforming drug delivery is also evident in the development of nanoplatforms that combine diagnostic imaging with targeted therapy. Kurul et al. [281] discussed how nanoparticles can be engineered to enhance cancer treatment outcomes by merging targeted delivery with advanced imaging techniques. This integration enables clinicians to visualize tumor margins accurately, monitor drug accumulation, and adjust treatment protocols dynamically, thereby improving overall efficacy [281]. Liposome-based nanocarriers have been extensively studied for their theranostic potential. A recent review by Azimizonuzi et al. [292] underscored the development of theranostic liposome hybrids that combine therapeutic agents with imaging contrast materials. These systems can be tailored to deliver chemotherapeutic drugs while simultaneously providing diagnostic signals, facilitating real-time assessment of treatment response. Their biocompatibility and ability to encapsulate diverse agents make them versatile platforms in cancer management [292].

Gold nanoparticles are another prominent class of theranostic agents, owing to their unique optical properties and ease of functionalization. Zhuang et al. [293] elaborated on their application in bridging detection and therapy, especially when conjugated with targeting ligands such as prostate-specific membrane antigen for prostate cancer. Gold nanoparticles can serve as contrast agents for imaging modalities like computed tomography and photoacoustic imaging, while also enabling photothermal therapy. Their multifunctionality exemplifies the potential of inorganic nanomaterials in integrated cancer theranostics [293]. The integration of biological ligands with nanocarriers further enhances targeting specificity. As discussed by Anitha et al. [270], biomimetic approaches involve conjugating nanocarriers with ligands that recognize tumor-specific markers, thereby creating single doses capable of simultaneous diagnosis and therapy. This strategy not only improved targeting accuracy but also reduces off-target effects, which is critical for clinical success. Recent advances also highlighted the importance of multifunctionality in nanoplatforms. Several studies [294,295] emphasized the design of nanoformulations that combine multiple diagnostic and therapeutic functions within a single system. Such multifunctional nanoformulations can provide comprehensive cancer management by enabling multimodal imaging, controlled drug release, and real-time monitoring of therapeutic responses.

In summary, the literature demonstrated that nanotechnology offers versatile and sophisticated platforms for cancer theranostics. Stimuli-responsive hydrogels, mesoporous silica nanoparticles, polymeric systems, carbon-based nanomaterials, liposomes, and gold nanoparticles each contribute unique advantages toward the goal of integrated diagnosis and treatment. The ongoing development of biomimetic and multifunctional nanoformulations further enhances the potential for personalized, precise oncology care. Despite these promising advances, challenges such as biocompatibility, targeted delivery efficiency, and clinical translation remain to be addressed. Nonetheless, the convergence of nanotechnology and oncology continues to pave the way for innovative theranostic solutions that could revolutionize cancer management in the near future.

### 16.3. Advanced Pre-Clinical Models: From 2D to Tumor Organoids and Humanized Mice

The evolution of pre-clinical models in oncology has been pivotal in advancing nanomedicine, enabling more accurate prediction of therapeutic efficacy and safety. Traditional 2D cell cultures have historically served as foundational platforms; however, their limitations in recapitulating the complex TME have prompted the development of more sophisticated models such as 3D cultures, tumor organoids, and humanized mouse models. This section highlights current insights into these models, emphasizing their roles, advantages, and challenges in nanomedicine research within oncology.

Initially, 2D cell cultures provided a straightforward and cost-effective means to study cancer biology and evaluate nanocarrier efficacy. Nonetheless, these models lack the architectural complexity and cellular heterogeneity of in vivo tumors, often leading to discrepancies between preclinical outcomes and clinical responses [296]. Recognizing these limitations, researchers have shifted focus toward 3D culture systems, including tumor spheroids and organoids, which better mimic the tumor architecture, cell–cell interactions, and extracellular matrix components. Such models have been endorsed as promising tools for preclinical studies, offering a more physiologically relevant environment for testing nanomedicines [297]. Tumor organoids, derived from patient tissues, have gained prominence due to their ability to preserve the genetic and phenotypic characteristics of original tumors. They serve as valuable platforms for personalized medicine, allowing for the assessment of nanomedicine responses in a patient-specific context. For instance, patient-derived tumoroids have been utilized to evaluate nanocarrier delivery and therapeutic efficacy, providing insights into individual tumor sensitivities [298]. These models facilitate high-throughput screening and can incorporate tumor heterogeneity, which is critical for understanding nanomedicine distribution and penetration within complex tumor tissues.

Complementing in vitro models, humanized mouse models have emerged as essential in vivo platforms that incorporate human immune components, thereby enabling the study of nanomedicine interactions within a human-like immune environment. Humanized mice, generated through engraftment of human hematopoietic stem cells, allow for the investigation of nanomedicine-induced immune responses and immunotherapy combinations. Studies have demonstrated that humanized mouse models can more accurately predict clinical immunogenicity and therapeutic outcomes, especially in the context of cancer immunotherapy [299]. Moreover, these models facilitate the evaluation of nanomedicine biodistribution, pharmacokinetics, and toxicity in a setting that closely resembles human physiology.

Recent advances have also integrated organ-on-chip technologies, which combine microfluidic systems with 3D cultures to simulate TME and vascularization. These platforms enable dynamic studies of nanocarrier transport, cellular interactions, and drug release under physiologically relevant flow conditions [296]. Such systems are particularly valuable for assessing nanomedicine penetration and efficacy in a controlled yet complex environment, bridging the gap between static cultures and animal models. The application of gene editing tools like CRISPR further enhances the utility of these models by allowing precise manipulation of tumor genetics, thereby enabling the study of nanomedicine targeting specific mutations. For example, Cas9-mediated editing of KRAS mutations in lung cancer models has provided insights into nanocarrier delivery and gene therapy strategies [300]. These genetically engineered models contribute to understanding resistance mechanisms and optimizing nanomedicine design. Despite these advancements, challenges remain. The complexity and cost of developing and maintaining organoids and humanized mice can be prohibitive. Additionally, standardization and reproducibility issues hinder widespread adoption. Nonetheless, the integration of AI-driven platforms and bioprinting technologies promises to address some of these limitations by enabling high-throughput, reproducible, and scalable model production [296].

In summary, the progression from 2D cultures to sophisticated 3D models, tumor organoids, and humanized mice has significantly enhanced the translational relevance of preclinical nanomedicine studies in oncology. These models offer a more accurate representation of tumor biology, immune interactions, and therapeutic responses, thereby facilitating the development of more effective and personalized nanotherapeutics. Continued innovation and integration of these advanced platforms are essential for overcoming current limitations and accelerating the translation of nanomedicine from bench to bedside.

## 17. Limitations and Future Prospects

Smart nanoformulations in oncology represent a promising frontier in cancer treatment, offering targeted drug delivery, reduced systemic toxicity, and improved therapeutic outcomes. However, their clinical application is hindered by several limitations, including challenges in clinical translation, biosafety concerns, and the complexity of the TME. Despite these challenges, the future prospects of smart nanoformulations are promising, with ongoing research focusing on enhancing their efficacy and safety.

- Tumor heterogeneity impact on targeting: Variability in receptor expression and TME heterogeneity limits the efficacy and consistency of active targeting ligands in nanoformulations. Develop adaptive or multi-ligand targeting systems that can dynamically respond to tumor heterogeneity; conduct comprehensive profiling of tumor receptor variability to guide ligand selection. Tumor heterogeneity causes inconsistent ligand binding and nanoparticle uptake, reducing therapeutic efficacy and clinical translation success [4,107,301].
- Stability and premature drug release of stimuli-responsive nanocarriers: Many stimuli-responsive nanocarriers suffer from premature drug release during circulation, compromising targeting and therapeutic outcomes. Engineer nanocarriers with enhanced stability in physiological conditions and precise stimuli-triggered release; integrate protective coatings or responsive gating mechanisms to prevent early leakage. Premature release reduces drug bioavailability at tumor sites and increases systemic toxicity, hindering clinical efficacy [179,302,303].
- Scalability and manufacturing reproducibility: Complex synthesis of multifunctional and multi-stimuli responsive nanocarriers challenges large-scale production and batch-to-batch consistency. Develop simplified, modular synthesis protocols amenable to scale-up; standardize characterization methods to ensure reproducibility and regulatory compliance. Manufacturing challenges delay clinical translation and regulatory approval of promising nanoformulations [107,302,304].
- Integration of multi-stimuli responsiveness without compromising biocompatibility: Designing nanocarriers responsive to multiple stimuli often increases complexity and may affect biocompatibility and safety profiles. Investigate biocompatible materials and architectures that allow multi-stimuli responsiveness; perform systematic toxicity and immunogenicity assessments in relevant models. Balancing responsiveness and safety is critical to avoid adverse effects and ensure clinical applicability [107,179,302].
- Overcoming biological barriers beyond EPR effect: EPR effect alone is insufficient due to tumor vasculature abnormalities, dense ECM, and high interstitial pressure limiting nanoparticle penetration. Explore active modulation of the TME (e.g., ECM degradation, vascular normalization) combined with size/charge-switchable nanoparticles to improve penetration and retention. Biological barriers cause heterogeneous nanoparticle distribution and suboptimal therapeutic outcomes [12,305,306].
- Limited clinical translation and long-term safety data: Despite promising preclinical results, few smart nanoformulations have advanced to clinical use due to insufficient long-term safety, immunogenicity, and regulatory data. Conduct longitudinal in vivo studies assessing chronic toxicity and immune responses; develop standardized preclinical models predictive of human outcomes; engage regulatory bodies early in development. Bridging preclinical–clinical gap is essential for successful adoption of smart nanoformulations [107,307].
- Optimization of external stimuli application in clinical settings: External stimuli (e.g., ultrasound, light, magnetic fields) require specialized equipment and protocols, limiting their practical clinical use. Design nanocarriers responsive to clinically feasible stimuli; develop portable, standardized devices for stimulus application; integrate imaging-guided stimulus delivery for precision. Practical challenges restrict widespread adoption of externally triggered drug release systems [4,308,309].
- Personalized nanomedicine and artificial intelligence integration: Current nanoformulations lack personalization to patient-specific TME and biology, limiting therapeutic precision. Incorporate artificial intelligence driven design and predictive modeling to tailor nanocarrier properties; develop patient-specific organ-on-chip models to test and optimize formulations preclinically. Personalized approaches can improve targeting accuracy and therapeutic outcomes, addressing tumor heterogeneity and patient variability [310].
- Pharmacokinetics and biodistribution understanding of complex nanocarriers: Insufficient knowledge of in vivo behavior, including circulation, clearance, and tumor accumulation of multifunctional nanocarriers. Perform detailed pharmacokinetic and biodistribution studies using advanced imaging and quantitative methods; correlate physicochemical properties with in vivo fate. Understanding nano–bio interactions is critical for rational design and clinical success [175,303].
- Controlled sequential and programmable drug release: Limited development of nanocarriers capable of programmable, sequential release of multiple drugs to maximize synergistic therapeutic effects. Design stimuli-free or multi-stimuli programmable systems enabling temporal control of drug release; validate efficacy in combination therapies targeting multidrug resistance. Sequential release can enhance combination therapy efficacy and reduce systemic toxicity [311,312].

In summary, the potential of smart nanoformulations in oncology is significant, it is essential to address the existing limitations to fully realize their benefits. The integration of advanced technologies, such as artificial intelligence, and the fostering of interdisciplinary collaborations are key to overcoming these challenges. Additionally, a balanced approach that considers both the therapeutic potential and safety concerns will be crucial in advancing the field of nano-oncology.

## 18. Conclusive Remarks

The literature on smart nanoformulations for oncology underscores significant progress in designing intelligent nanocarriers that effectively overcome biological barriers inherent to the TME. Collectively, research demonstrated that active targeting using ligands such as antibodies, peptides, aptamers, and immune-recognition elements markedly enhances tumor specificity and cellular uptake, facilitating higher nanoparticle accumulation and retention within tumors. The integration of passive targeting via the enhanced permeability and retention effect further synergizes with active targeting strategies, optimizing localization to malignant tissues. However, consistent challenges persist, including ligand stability, immunogenicity, and the heterogeneous nature of tumor receptor expression, which may impede universal applicability and clinical translation.

Stimuli-responsive mechanisms emerge as pivotal for achieving precise, controlled drug release at tumor sites while minimizing systemic toxicity. The literature indicated utilization of endogenous stimuli—such as acidic pH, elevated glutathione levels, enzymatic overexpression, hypoxia, and reactive oxygen species—as reliable triggers for site-specific payload release. Additionally, exogenous stimuli like light, ultrasound, magnetic fields, and temperature contribute spatiotemporal control, enhancing therapeutic precision. Increasingly, multi- and dual-stimuli responsive nanocarriers are explored to address tumor complexity and improve synergistic treatment outcomes. Despite these advances, challenges remain regarding the stability of stimuli-responsive systems in circulation, premature drug release, and the need for specialized equipment for exogenous stimuli application, limiting their widespread clinical adoption.

Overcoming the multifaceted biological barriers—such as abnormal tumor vasculature, dense extracellular matrix, high interstitial fluid pressure, and intracellular trafficking constraints—is critical for enhancing drug bioavailability and therapeutic efficacy. Innovative approaches including charge-reversal nanoparticles, size-switchable carriers, and transcytosis-based delivery have demonstrated improved tumor penetration and cellular internalization. Nonetheless, tumor heterogeneity and dynamic microenvironmental factors continue to cause uneven distribution and suboptimal therapeutic effects. Immune clearance and off-target accumulation remain significant concerns that must be addressed to realize effective clinical translation.

While numerous studies report promising safety profiles, enhanced efficacy, and reduced systemic toxicity, the transition to clinical success is hindered by challenges including long-term safety data paucity, regulatory complexities, manufacturing scalability, and reproducibility of complex nanocarrier systems. Emerging trends such as artificial intelligence-driven personalization, theranostic integration, and organ-on-a-chip models offer promising avenues to overcome these translational barriers. Future research must focus on developing standardized evaluation protocols, improving biocompatibility, and establishing scalable manufacturing processes to facilitate regulatory approvals and clinical adoption. Overall, the literature provides a robust foundation of knowledge and innovative strategies, yet underscores the critical need for continued multidisciplinary efforts to bridge preclinical advancements with clinical realities, ultimately enabling the full therapeutic potential of smart nanoformulations in cancer treatment.

## Figures and Tables

**Table 13:** Nanoformulation strategies to overcome key mechanisms of multidrug resistance in cancer.

Mechanism	Challenge	Nanoformulation Strategy	Example(s)	Outcome(s)	Ref.
ABC transporter efflux	Overexpression of P-gp/ABCB1, MRP1/ABCC1, BCRP/ABCG2 pumps drugs out of cells	Co-delivery of P-gp inhibitors Bypass efflux via nanocarrier uptake Stimuli-triggered intracellular release	Doxorubicin + Elacridar in liposomes Polymeric nanoparticles encapsulating chemotherapeutics Redox/pH-sensitive nanoparticles for rapid cytosolic release	Increased intracellular drug concentration, reversing transporter-mediated resistance	[261,262]
Altered drug targets and signaling	Mutations in drug targets; activation of pro-survival pathways (e.g., PI3K/Akt)	Co-delivery of targeted inhibitors Nano-PROTACs for protein degradation	Nanoparticles co-loaded with chemo + kinase inhibitor (e.g., Gefitinib) Nucleic acid-based nanoparticles targeting oncogenic transcription factors	Synergistic cell death Degradation of resistant protein targets	[125,263]
Enhanced DNA repair and anti-apoptosis	Upregulation of DNA repair mechanisms (e.g., ERCC1); evasion of apoptosis (e.g., Bcl-2)	Delivery of DNA repair inhibitors Co-delivery of pro-apoptotic agents	Nanoparticles carrying PARP inhibitors Nanoparticles with cisplatin + Bcl-2 siRNA	Increased sensitivity to DNA-damaging agents Restoration of apoptotic pathways	[259,260]
TME-mediated resistance	Hypoxia, acidic pH, and dense stroma limit drug penetration and efficacy	TME-modulating nanocarriers Nitric oxide releasing nanomedicines	Hypoxia-responsive nanoparticles releasing stroma-degrading enzymes Metal–organic frameworks that generate nitric oxide	Improved drug perfusion and penetration Reversal of TME-driven resistance	[264,265]
Dysregulated metabolism	Metabolic adaptations (e.g., glycolysis, oxidative stress) promote cell survival	Metabolic pathway interference Cuproptosis inducers	Nanoparticles loaded with glycolytic inhibitors (e.g., 2-DG) Copper-based nanomaterials	Disruption of energy production Induction of novel metal-induced cell death	[266,267]
Inhibition of cell death pathways	Resistance to apoptosis, ferroptosis, and other forms of programmed cell death	Inducers of alternative cell death Ferroptosis-promoting nanoparticles	Nanoparticles delivering ferroptosis inducers (e.g., RSL3, Erastin) Artesunate + temozolomide liposomes	Bypassing apoptotic resistance Effective killing of resistant cell populations	[252,256]

## Data Availability

No new data were created or analyzed in this study. Data sharing is not applicable to this article.

## Abbreviations

The following abbreviations are used in this manuscript:

ABC	ATP-binding cassette
AuNGs	Gold nanogels
BBB	Blood–brain barrier
BBTB	Blood–brain tumor barrier
CAFs	Cancer-associated fibroblasts
ECM	Extracellular matrix
EGFR	Epidermal growth factor receptor
EPR	Enhanced permeability and retention
HMONs	Hollow mesoporous organosilica nanoparticles
IMDO-N_3_	Azide modified imidazoquinolines
MOFs	Metal–organic frameworks
NED	Neuroendocrine differentiation
PD-L1	Programmed death-ligand 1
PEGylation	Functionalization with Poly(ethylene glycol)
PEH-HIONs	Perfluorohexane encapsulated superparamagnetic hollow iron oxide nanoparticles
PEPCA	Poly(ethylene glycol)-poly(cinnamyl aldehyde)
PRMT5	Protein arginine methyltransferase 5
RBCM	Red blood cell membrane
Tm@PDA-GA	4T1 membrane@polydopamine-gambogic acid
TME	Tumor microenvironment
TIME	Tumor immune microenvironment
